# Antennae-enriched expression of candidate odorant degrading enzyme genes in the turnip aphid, *Lipaphis erysimi*

**DOI:** 10.3389/fphys.2023.1228570

**Published:** 2023-07-05

**Authors:** Chaozhi Shangguan, Yinhui Kuang, Liwei Gao, Bo Zhu, Xue Dong Chen, Xiudao Yu

**Affiliations:** ^1^ Ganzhou Key Laboratory of Nanling Insect Biology/National Navel Orange Engineering Research Center, College of Life Sciences, Gannan Normal University, Ganzhou, Jiangxi, China; ^2^ Entomology and Nematology Department, University of Florida, Gainesville, FL, United States

**Keywords:** turnip aphid, comparative transcriptome analysis, odorant degrading enzyme, antennae-enriched, gene expression

## Abstract

Aphids heavily rely on their olfactory system for foraging behavior. Odorant-degrading enzymes (ODEs) are essential in preserving the olfactory acuity of aphids by removing redundant odorants in the antennae. Certain enzymes within this group stand out as being enriched and/or biased expressed in the antennae, such as carboxylesterases (CXEs), cytochrome P450 (CYPs), glutathione S-transferases (GSTs), and UDP-glycosyltransferases (UGTs). Here, we performed a comparative transcriptome analysis of antennae and body tissue to isolate the antennal ODE genes of turnip aphid *Lipaphis erysimi*. A dataset of one CXE, seven CYPs, two GSTs, and five UGTs enriched in the antennae was identified and subjected to sequence analysis. Furthermore, qRT-PCR analyses showed that 13 ODE genes (*LeCXE6*, *LeCYP4c1*, *LeCYP6a2*, *LeCYP6a13*, *LeCYP6a14.2*, *LeCYP6k1*, *LeCYP18a1*, *LeGST1*, *LeUGT1-7*, *LeUGT2B7*, *LeUGT2B13*, *LeUGT2C1.1*, and *LeUGT2C1.2*) were specifically or significantly elevated in antennal tissues. Among these antennae-enriched ODEs, *LeCYP4c1*, *LeCYP6a2*, *LeCYP6a13*, *LeCYP6a14.2*, *LeCYP18a1*, *LeUGT2B7*, and *LeUGT2B13* were found to exhibit significantly higher expression levels in alate aphids compared to apterous and nymph aphids, suggesting their putative role in detecting new host plant location. The results presented in this study highlight the identification and expression of ODE genes in *L. erysimi*, paving the path to investigate their functional role in odorant degradation during the olfactory processes.

## 1 Introduction

Insect antennae are intricate sensory organs essential in detecting a variety of lipophilic volatiles from the environment, helping insects secure food, find mates, lay eggs, and steer clear of potential predators (Leal, 2013; Cheema et al., 2021). During these biologic processes, the exogenous odor molecules are initially bound with insect odorant-binding proteins (OBPs) and chemosensory proteins (CSPs); they then move through the sensillum lymph and interact with olfactory receptors (ORs) situated on the membrane surface of olfactory sensory neurons. The ORs convert the chemical signals from the odor molecules into electrophysiological signals, which can be deciphered by the brain (Leal, 2013; Pelosi et al., 2018; Cheema et al., 2021; Zhou and Jander, 2022). Subsequently, antennal enzymes called odorant-degrading enzymes (ODEs) present in the vicinity of ORs become critical for the rapid degradation of odorant molecules, allowing for the insect’s olfactory system to recover and maintain its sensitivity (Younus et al., 2014; Blomquist et al., 2021; Chertemps and Maïbèche, 2021).

Insect ODEs are primarily recognized for their crucial role in metabolizing endogenous hormones and exogenous compounds like xenobiotics and allelochemicals. They include a few antennae-biased or antennae-enriched carboxylesterases (CXEs), cytochrome P450 (CYPs), glutathione S-transferases (GSTs), UDP-glycosyltransferases (UGTs), aldehyde oxidases (AOXs) (Younus et al., 2014; Blomquist et al., 2021). Among these ODEs, insect CXEs could degrade ester, amide, and carbamate bonds found in a range of plant volatiles, insect pheromones, hormones, and many pesticides (Leal, 2013; Ding et al., 2022). The first ODE identified was *ApolSE*, a CXE gene that was highly prevalent within the antennae of male silkmoths *Antheraea polyphemus* (Vogt and Riddiford, 1981); subsequent functional analyses determined that *ApolSE* functioned as a pheromone degrading enzyme, effectively breaking down the sex pheromone components [(6E, 11Z)-hexadecadienyl acetate, Z11-16:Ac] (Vogt et al., 1985; Ishida and Leal, 2005). Since then, several additional antennal CXEs have been functionally identified in various insects, such as the cotton leafworm *Spodoptera littoralis* (Durand et al., 2010), beet armyworm *Spodoptera exigua* (He et al., 2015), German cockroach *Blattella germanica* (Ma et al., 2023), and oriental fruit moth *Grapholita molesta* (Wei et al., 2021).

Insect CYPs are another well-studied group of antennal ODEs (Blomquist et al., 2021; Wu et al., 2022). While their primary function is to detoxify chemical insecticides within the body, studies in the pine beetle *Dendroctonus ponderosae* documented that several CYPs (e.g., CYP345E2, CYP6DE1, CYP6DJ1, CYP6BW1, and CYP6BW3) were capable of removing terpenoids from antennae and detoxifying host terpenoids to overcome plant defenses (Chiu et al., 2019a; Chiu et al., 2019b; Keeling et al., 2013). Additionally, certain enzymes within GST, UGT and AOX groups were reported to be linked to odorant and xenobiotic degradation (Rogers et al., 1999; Bozzolan et al., 2014; Li et al., 2018; Fraichard et al., 2020; Wang et al., 2021b; Liu et al., 2021). For example, a GST called *GST-msolf1* restricted to pheromone sensilla could inactivate the sex pheromone blend in the tobacco hornworm, *Manduca sexta* (Rogers et al., 1999); *UGT36E1*, a UGT enzyme gene abundant in *Drosophila* antennal olfactory sensory neurons was involved in the clearance of pheromones (Fraichard et al., 2020), and the antennal aldehyde oxidase gene from the diamondback moth *Plutella xylostella*, *PxylAOX3*, oxidized both sex pheromone compounds and plant-derived aldehydes (Wang et al., 2021a).

The turnip aphid, *Lipaphis erysimi* Kaltenbach, poses a significant threat to the cultivation of *Brassica* vegetables and oilseed crops due to its direct feeding and/or transmission of harmful plant viruses. RNA interference (RNAi) has emerged as a promising strategy for controlling aphids, and antennal ODEs hold great promise as the optimal target genes for disrupting foraging behaviors (Yu et al., 2014; Yu et al., 2016; Wei et al., 2021; Wu et al., 2022; Ma et al., 2023). In this study, we aimed to identify antennae-enriched ODE genes of this aphid species by conducting as follows: 1) performing comparative analysis on the antennal and body transcriptomes of *L. erysimi*; 2) isolating and *in silico* analysis of candidate ODE genes; 3) identifying the expression profile of the ODE genes among different tissues and developmental stages.

## 2 Materials and methods

### 2.1 Insect rearing

The colony of *L. erysimi* utilized in this study was established in 2020, based on the field populations from Xinfeng in the Jiangxi province of China (Kuang et al., 2023). The population was continuously maintained on Chinese cabbage Shanghaiqing (*Brassica rapa* var. *chinensis*) without exposure to any insecticides under controlled conditions (27°C–28°C, 60%–65% RH, 14:10 L:D photoperiod).

### 2.2 Sample preparation, RNA extraction, and cDNA synthesis

Samples of *L. erysimi* were collected at five different stages, which include 1st instar nymph, 2nd instar nymph, 3rd instar nymph, 4th instar nymph, 1-day apterous and alate adults. The apterous aphids anesthetized on ice were dissected under a stereomicroscope to collect various tissues of *L. erysimi*, such as antenna, head, leg, and cuticle. Total RNA extraction was performed according to the manufacturer’s protocol of Trizol reagent (Sigma, St. Louis, MO, United States). The purity and concentration of RNAs were determined using a NanoDrop One^C^ spectrophotometer from Thermo Fisher Scientific (Waltham, MA, United States). The first-strand cDNA synthesis was synthesized with 500 ng of purified RNA, utilizing the highly effective EasyScript^®^ One-Step gDNA Removal and cDNA Synthesis SuperMix Kit (TransGen Biotech, Beijing, China). The resulting cDNA was properly stored at −20°C until ready to be used.

### 2.3 Comparative transcriptome analysis

To isolate the antennal-biased genes in *L. erysimi*, the high-throughput transcriptome data sets were retrieved from our former study (GenBank accession number PRJNA947784), which included conducting Illumina sequencing on the antennae and bodies (excluding antennae) of adult apterous aphids, transcriptome *de novo* assembly, as well as functional annotation of the unigenes (Kuang et al., 2023). The differential gene expression analysis was carried out using the antennal and body transcriptome data as described by Yu et al. (2023). Briefly, the transcript abundances were determined by RSEM (version 1.2.12); DESeq2 (version 1.4.5) was utilized to identify differentially expressed genes (DEGs) between samples, and a gene was considered differentially expressed if the corrected *p*-value was ≤ 0.05.

### 2.4 Identification of candidate ODE genes

The antennae-biased ODE genes with FPKM ≥10 were selected from the gene repertories obtained through comparative transcriptome analysis. Candidate ODEs were confirmed using the BLASTX algorithm, and their open reading frames (ORFs) were predicted using the ORF finder tool (http://www.ncbi.nlm.nih.gov/gorf/gorf.html). The amino acid components, theoretical isoelectric points (pIs), and molecular weights (MWs) of ODE genes were calculated using ExPASy (http://web.expasy.org/protparam/). The deduced protein sequences were submitted to the SignalP 5.0 server (https://services.healthtech.dtu.dk/services/SignalP-5.0/) for the prediction of the signal peptide sequences and their corresponding cleavage sites. Conserved domains were predicted with CDD-BLAST (https://www.ncbi.nlm.nih.gov/Structure/cdd/wrpsb.cgi) and InterProScan (https://www.ebi.ac.uk/interpro/) servers.

### 2.5 Phylogenetic analysis

The dataset submitted for phylogenetic analysis comprised the candidate CXE, CYP, GST, and UGT genes of *L. erysimi* in this study, their homologs from *Acyrthosiphon pisum* (Ramsey et al., 2010), *Aphis craccivora* (Yang et al., 2021), *Aphis gossypii* (Pan et al., 2018), *Myzus persicae* (Pan et al., 2019), *Nilaparvata lugens* (Vontas et al., 2002; Zhou et al., 2013), *Diaphorina citri* (Yu and Killiny, 2018; Tian et al., 2019; Wu et al., 2020; Kuang et al., 2022), *Anopheles gambiae* (Ding et al., 2003), and *Bombyx mori* (Yu et al., 2008); in addition to some well-identified ODE genes, including the studied antennal CXEs (Ishida and Leal, 2005; 2008; Durand et al., 2010; Durand et al., 2011; He et al., 2015; Wei et al., 2021), CYPs (Keeling et al., 2013; Chiu et al., 2019a), GSTs (Rogers et al., 1999; Li et al., 2018; Liu et al., 2021; Xia et al., 2022), and UGTs (Wang et al., 1999; Bozzolan et al., 2014). The protein sequences were first aligned using CLUSTAL_X version 1.83. The joint unrooted phylogenetic tree was constructed with MEGA11 using the neighbor-joining method (Tamura et al., 2021). Branch support was evaluated through the bootstrap method which consisted of 1,000 replicates. The phylogenetic tree was visualized using iTOL web tool (https://itol.embl.de/).

### 2.6 Quantitative real-time PCR analysis

The quantitative real-time PCR (qRT-PCR) reactions were conducted in a 20 μL volume that comprised of 4 μL of diluted cDNA, 0.4 μM of each primer, and 10 μL of PerfectStart^®^ Green qPCR SuperMix (TransGen Biotech, Beijing, China). Reactions were performed on a Roche LightCycler 96^®^ system (Roche Diagnostics, Mannheim, Germany) with the following thermal program: initial denaturation for 10 min at 95°C, followed by a 40-cycle two-step amplification profile of 95°C for 5 s and 60°C for 30 s. Two reference genes, actin (GenBank accession number OQ626608) and GAPDH (GenBank accession number OQ626609), were employed to standardize the quantity of cDNA added to the PCR reactions. The relative expression of each ODE gene was analyzed using the 2^−ΔΔCt^ method (Livak and Schmittgen, 2001). Reactions were performed in triplicate, and the gene-specific primers are listed in Table 1.

**TABLE 1:** Oligonucleotide primer pairs used in this study.

Gene	Primer name	Sequences of primers (5′→3′)	Application
*LeCXE6*	LeCXE6 F	AAG​GAG​GCA​CAG​CCA​ATA​AA	qRT-PCR
	LeCXE6 R	CCT​CGG​CTC​CTT​CAA​TCA​AAT​A	
*LeCYP6a13*	LeCYP6a13 F	TCA​AAG​AGT​GCG​GTG​ACT​TAT​T	qRT-PCR
	LeCYP6a13 R	ACT​TTC​CCA​TGA​TGT​CCC​TTA​TC	
*LeCYP18a1*	LeCYP18a1 F	ACA​TCA​TCG​AGG​AAC​ACA​AGA​G	qRT-PCR
	LeCYP18a1 R	GGC​TTC​TTG​GGA​GCG​ATT​TA	
*LeCYP6a2*	LeCYP6a2 F	GAC​GGA​CCT​AGA​TTG​TGC​ATA​G	qRT-PCR
	LeCYP6a2 R	CGC​ACG​GTA​TGA​CTT​CGT​ATT	
*LeCYP4C1*	LeCYP4C1 F	CTG​GGA​CTA​TAT​CGC​ACC​ATT​T	qRT-PCR
	LeCYP4C1 R	TGC​TTC​GCC​AAA​TTC​ACA​TTC	
*LeCYP6k1*	LeCYP6k1 F	CAG​ACC​GAA​TCG​ACG​TGA​AA	qRT-PCR
	LeCYP6k1 R	TCA​GAG​TCG​TCG​TTC​TTG​ATT​G	
*LeCYP6a14.1*	LeCYP6a14.1 F	TGA​GTT​TGA​CCG​CCG​TTA​TC	qRT-PCR
	LeCYP6a14.1 R	GTA​CCG​GTG​GTA​TAT​GTG​GTA​TG	
*LeCYP6a14.2*	LeCYP6a14.2 F	GAT​GAA​GTA​CAG​GGA​GGA​ACA​C	qRT-PCR
	LeCYP6a14.2 R	GGC​CAC​GAT​ATC​CGT​TTC​TAA	
*LeGST1*	LeGST1 F	GCA​AAG​GAG​GTG​GAG​AAG​TTA​G	qRT-PCR
	LeGST1 R	TGC​CAT​CAT​TTC​TGG​AGG​TTT​A	
*LeGST*	LeGST F	GCT​GCA​AAG​TAT​GTC​ACG​TTA​G	qRT-PCR
	LeGST R	GCC​CAA​GAT​AAC​TTT​CCG​TTT​AC	
*LeUGT2B7*	LeUGT2B7 F	CGA​GGG​TGA​AAT​GAA​GGA​CAA	qRT-PCR
	LeUGT2B7 R	GAC​ATA​CCT​CCG​TGA​CTG​ATA​AAG	
*LeUGT2B13*	LeUGT2B13 F	ACC​GTG​GTC​TGC​TGT​TTA​TC	qRT-PCR
	LeUGT2B13 R	CTC​TTA​CCC​GCT​ATC​GTT​TCC	
*LeUGT1-7*	LeUGT1-7 F	GCG​TGA​GCG​GAG​TAT​TCA​TTA​T	qRT-PCR
	LeUGT1-7 R	CTG​TAC​TTC​TGG​GTC​GGA​TAG​A	
*LeUGT2C1.1*	LeUGT2C1.1 F	GCT​CGA​GCA​AAT​GCT​GAA​TAA​C	qRT-PCR
	LeUGT2C1.1 R	GCA​TTC​CTC​CTA​CTT​CGA​TGA​C	
*LeUGT2C1.2*	LeUGT2C1.2 F	TAC​ATC​GAA​CCC​AGG​GAG​TA	qRT-PCR
	LeUGT2C1.2 F	GTG​GAT​GAT​GGA​TGG​CAG​AA	
*LeryActin*	LeryActin F	GCT​CTA​TTC​CAA​CCT​TCC​TTC​T	qRT-PCR
	LeryActin R	GGC​GTA​CAA​GTC​CTT​ACG​AAT​A	
*LeryGAPDH*	LeryGAPDH F	GGA​TCT​GCT​GGT​GCT​GAT​TA	qRT-PCR
	LeryGAPDH R	ACT​TTC​TTG​GCT​CCA​CCT​TC	

### 2.7 Statistical analysis

The statistical differences of ODE gene expression levels among different developmental stages and tissues were analyzed using analysis of variance (ANOVA), followed by Tukey multiple comparison test. The statistical analysis was conducted using GRAPHPAD PRISM software (version 6.0; GraphPad Software Inc., La Jolla, CA, United States) and a *p*-value of ≤ 0.05 was set as the threshold for statistical significance.

## 3 Results

### 3.1 Differentially expressed genes analysis

**FIGURE 1:**
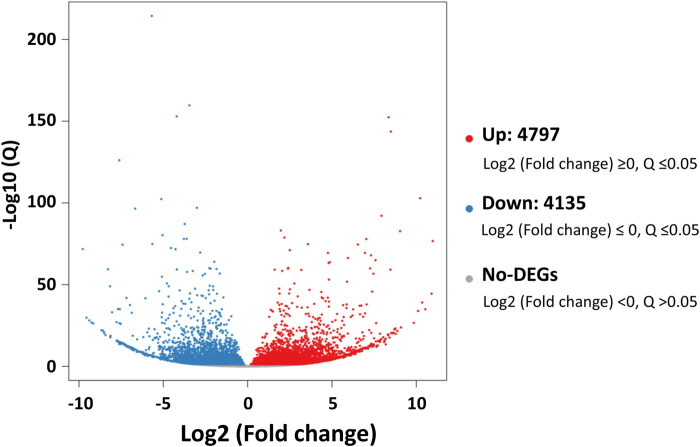
The differentially expressed genes (DEGs) between *Lipaphis erysimi* antennal and body transcriptomes. Red dots indicated the unigenes upregulated in antennae; blue dots indicated the downregulated unigenes in antennae by comparing with body samples.

Comparative analyses of the antennal and body transcriptomes in this study provide useful information to identify the antennae-abundant and/or antennae-biased genes. A total of 8,932 differentially expressed genes (DEGs) with a Q value ≤ 0.05 were identified, and 4,797 DEGs were significantly upregulated in the antennae (Figure 1). Among the antennae-abundant DEGs, one CXE, seven CYPs, two GSTs, and five UGTs were identified by blasting against the Nr database. The candidate ODEs were designated according to gene names of the top blast hits in NCBI. Detailed information on these ODE enzymes is shown in Table 2.

**TABLE 2:** Sequence information of antennae-enriched ODE genes in *Lipaphis erysimi*.

Designation	Transcriptomic data (Mean FPKM)		ORF (aa)	Mw (kDa)	pI	SP	Blastx best hit (Reference/Name/Species)
	Antenna	Body					
LeCYP6a13	1,220.17 ± 725.87	32.84 ± 5.18	459	53.63	6.57	N	|XP_001948581.2| probable cytochrome P450 6a13 [*Acyrthosiphon pisum*]
LeCYP18a1	266.75 ± 161.10	22.80 ± 4.03	512	58.32	6.47	N	|XP_015366692.1| cytochrome P450 18a1 [*Diuraphis noxia*]
LeCYP6a2	241.20 ± 117.82	11.95 ± 2.54	432	49.62	6.97	N	|XP_001947920.1| cytochrome P450 6a2 [*Acyrthosiphon pisum*]
LeCYP4c1	151.39 ± 91.57	10.55 ± 0.75	457	53.15	8.61	N	|XP_008181889.1| cytochrome P450 4C1-like [*Acyrthosiphon pisum*]
LeCYP6k1	62.67 ± 30.83	2.15 ± 0.21	514	59.29	6.73	N	|XP_015379337.1| cytochrome P450 6k1-like [*Diuraphis noxia*]
LeCYP6a14.1	45.49 ± 8.60	1.88 ± 0.64	512	59.20	7.56	N	|NP_001352523.1| probable cytochrome P450 6a14 [*Myzus persicae*]
LeCYP6a14.2	29.83 ± 11.88	0.01 ± 0.02	519	59.04	7.23	N	|XP_001945100.2| probable cytochrome P450 6a14 [*Acyrthosiphon pisum*]
LeGST1	93.81 ± 25.59	5.06 ± 0.55	157	17.89	9.00	N	|XP_026815723.1| microsomal glutathione S-transferase 1-like [*Rhopalosiphum maidis*]
LeGST	27.88 ± 8.72	3.97 ± 2.96	198	23.11	5.16	N	|XP_022171305.1| glutathione S-transferase-like [*Myzus persicae*]
LeUGT2B7	404.61 ± 171.22	17.19 ± 1.05	513	58.07	8.98	1–28	|XP_022162082.1| UDP-glucuronosyltransferase 2B7-like isoform X6 [*Myzus persicae*]
LeUGT2B13	40.55 ± 8.31	0.12 ± 0.04	542	60.85	8.31	N	|XP_015366788.1| UDP-glucuronosyltransferase 2B13-like [*Diuraphis noxia*]
LeUGT1-7	38.46 ± 21.69	3.82 ± 0.56	520	61.03	8.31	1–20	|XP_015368469.1| UDP-glucuronosyltransferase 1-7-like [*Diuraphis noxia*]
LeUGT2C1.1	38.09 ± 12.32	7.02 ± 1.11	515	58.12	6.90	1–26	|XP_015370078.1| UDP-glucuronosyltransferase 2C1-like isoform X1 [*Diuraphis noxia*]
LeUGT2C1.2	21.06 ± 10.63	3.99 ± 0.18	521	58.75	9.03	1–24	|XP_001949466.2| UDP-glucuronosyltransferase 2C1-like [*Acyrthosiphon pisum*]
LeCXE6	224.04 ± 137.60	19.95 ± 1.56	564	62.73	5.57	1–18	|XP_015373999.1| venom carboxylesterase-6-like isoform X1 [*Diuraphis noxia*]

Note: aa, amino acids; Mw, molecular weight; pI, isoelectric points; SP, signal peptide.

### 3.2 Identification of putative CXE genes

**FIGURE 2:**
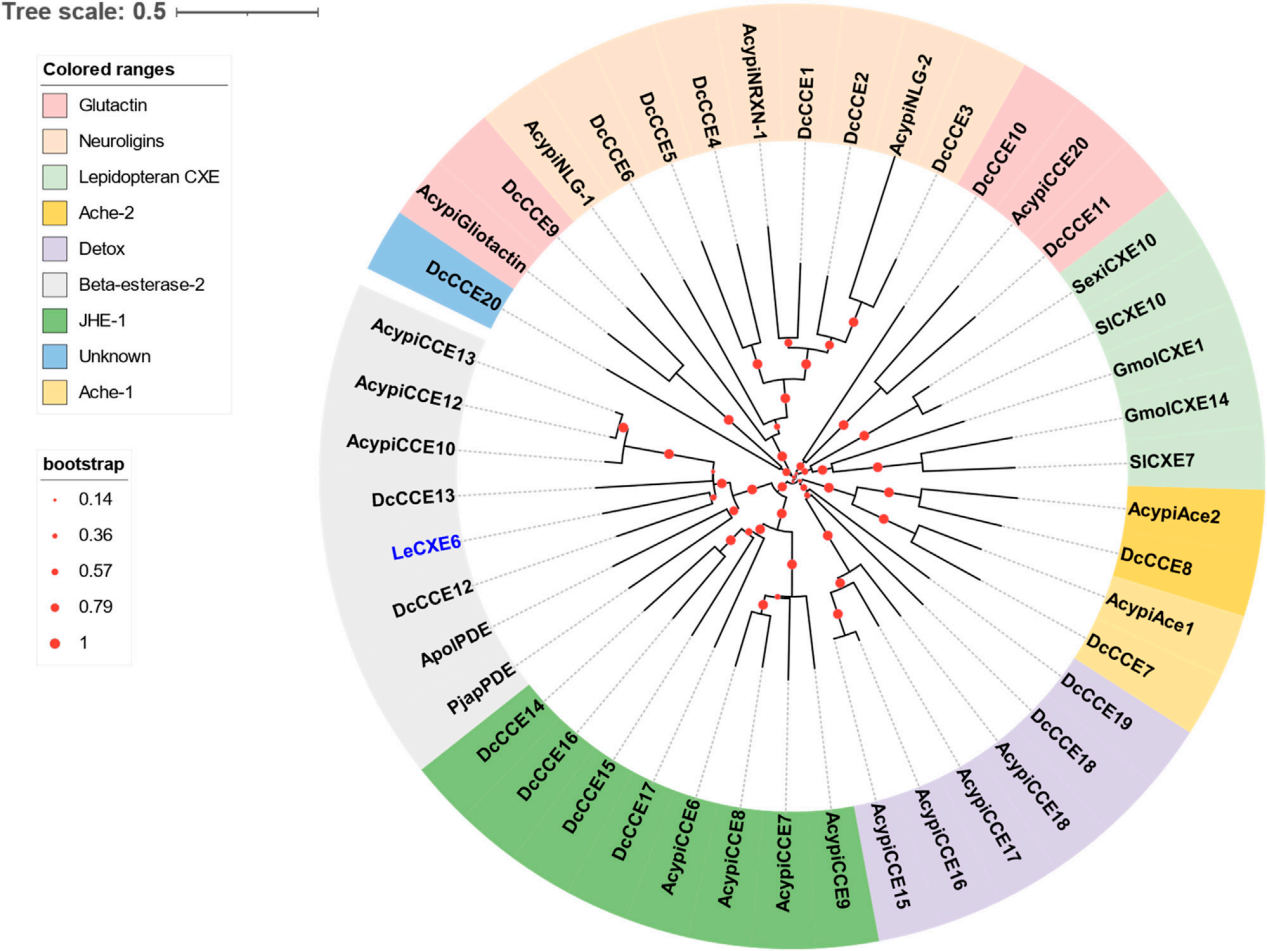
The Phylogenetic relationship of 46 CXE/CCE proteins from the aphid species *L. erysimi* (Le, 1), *Acyrthosiphon pisum* (Acypi, 18), and the hemipteran insect *Diaphorina citri* (Dc, 20); as well as the well-studied antennal CXEs from *Grapholita molesta* (GmolCXE1 and GmolCXE14), *Spodoptera littoralis* (SlCXE7 and SlCXE10), *Spodoptera exigua* (SexiCXE10), *Antheraea polyphemus* (ApolPDE), and *Popillia japonica* (PjapPDE). The LeCXE isolated in this study is highlighted in blue. The neighbor-joining (NJ) tree was constructed using MEGA 11 with 1,000 bootstrap replicates. The CCE protein sequences used in phylogenetic analysis are listed in Supplementary Table S1.

One putative CXE gene, *LeCXE6*, showed a high level of expression in the antennae, with FPKM values over 10 times higher than in the rest of the body. *LeCXE6* encodes a 564 amino-acid protein, with a signal sequence cleavage site predicted between Gly-18 and Phe-19 at the N-terminus. The predicted protein has a theoretical molecular mass of 62.73 kDa and an isoelectric point of 5.57, as determined using ProtParam tool in Expasy server (Table 2). Conserved domain and sequence alignment analysis revealed that LeCXE6 contained the typical motif of carboxylesterase family, including a conserved pentapeptide (Gly-X-Ser-X-Gly) and an oxyanion hole (Gly-Gly-Ala; Supplementary Figure S1). Phylogenetic analysis showed that LeCXE6 fell into the beta-esterase clade and was closely clustered with the well-studied odorant-degrading enzymes, PjapPDE and ApolPDE (Figure 2).

### 3.3 Identification of putative CYP genes

**FIGURE 3:**
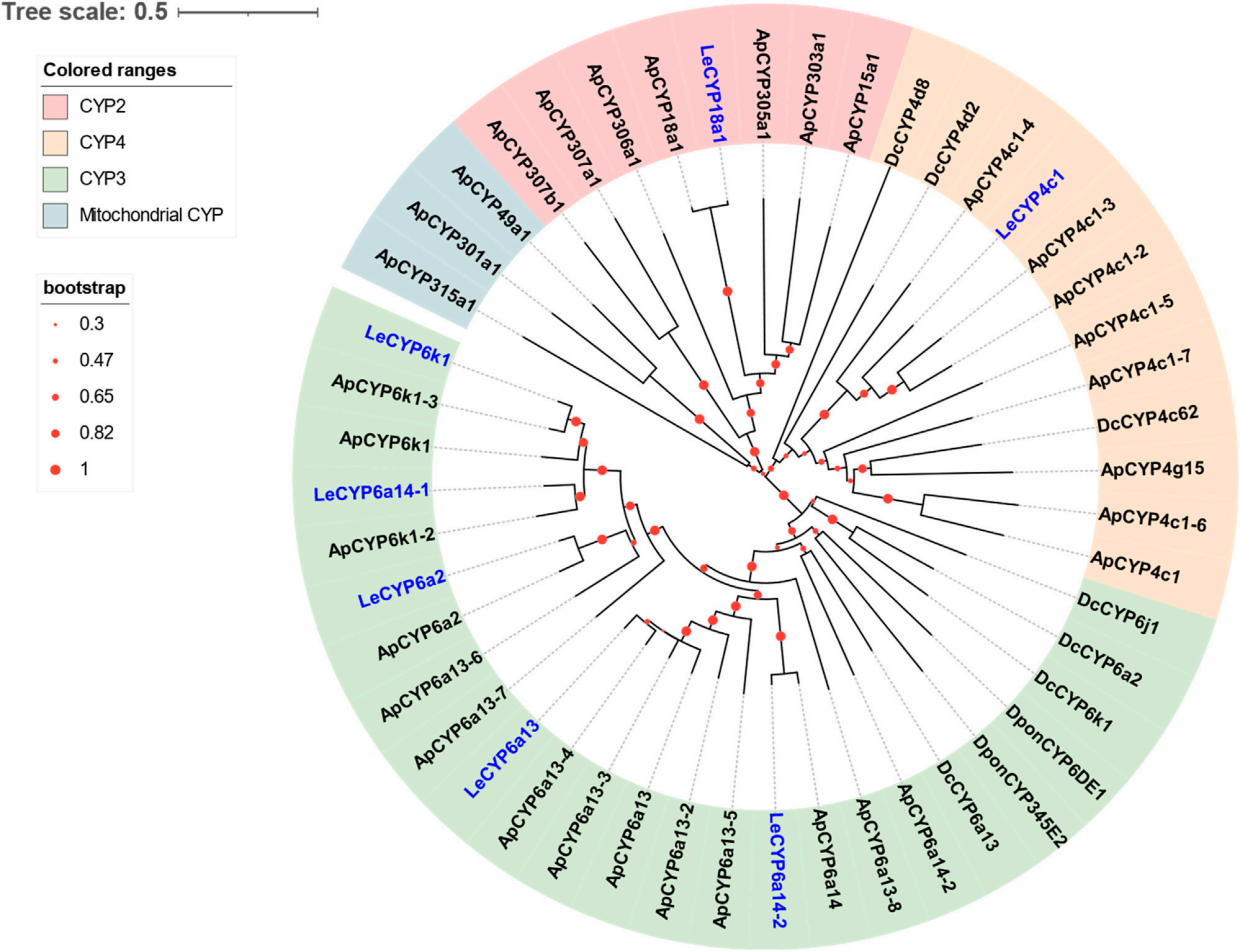
Phylogenetic tree of 48 CYPs from the aphid species *L. erysimi* (Le, 7), *A. pisum* (Ap, 32), and the hemipteran insect *D. citri* (Dc, 7); as well as two well-studied antennal CYPs, DponCYP345E2 and DponCYP6DE1, from *Dendroctonus ponderosae*. The neighbor-joining (NJ) tree was constructed using MEGA 11 with 1,000 bootstrap replicates. Seven *L. erysimi* CYPs are highlighted in blue. The CYP sequences used in this phylogenetic tree are provided in Supplementary Table S2.

Seven DEGs abundant in *L. erysimi* antennae were identified to be CYPs by blasting against the Nr database. All candidate LeCYPs were found to contain full-length ORFs without any predicted signal peptide sequences. Their antennal RPKM values ranged from 29.83 to 1,220.17, representing more than a ten-fold increase in comparison to the body group. Among them, *LeCYP6a13* was the most abundant CYP gene in antennae with a value exceeding 1,200, followed by *LeCYP18a1* and *LeCYP6a2* (Table 2). Phylogenetic analysis showed that the selected CYPs were well categorized into four subclasses, namely, CYP2, CYP3, CYP4, and mitochondrial CYP. The CYP3 class comprised of five antennal LeCYPs, including LeCYP6a13, LeCYP6a2, LeCYP6k1, LeCYP6a14.1, and LeCYP6a14.2. LeCYP18a1 was classified as a member of the CYP2 clan, while LeCYP4c1 was categorized into the CYP4 clade (Figure 3).

### 3.4 Identification of putative GST genes

**FIGURE 4:**
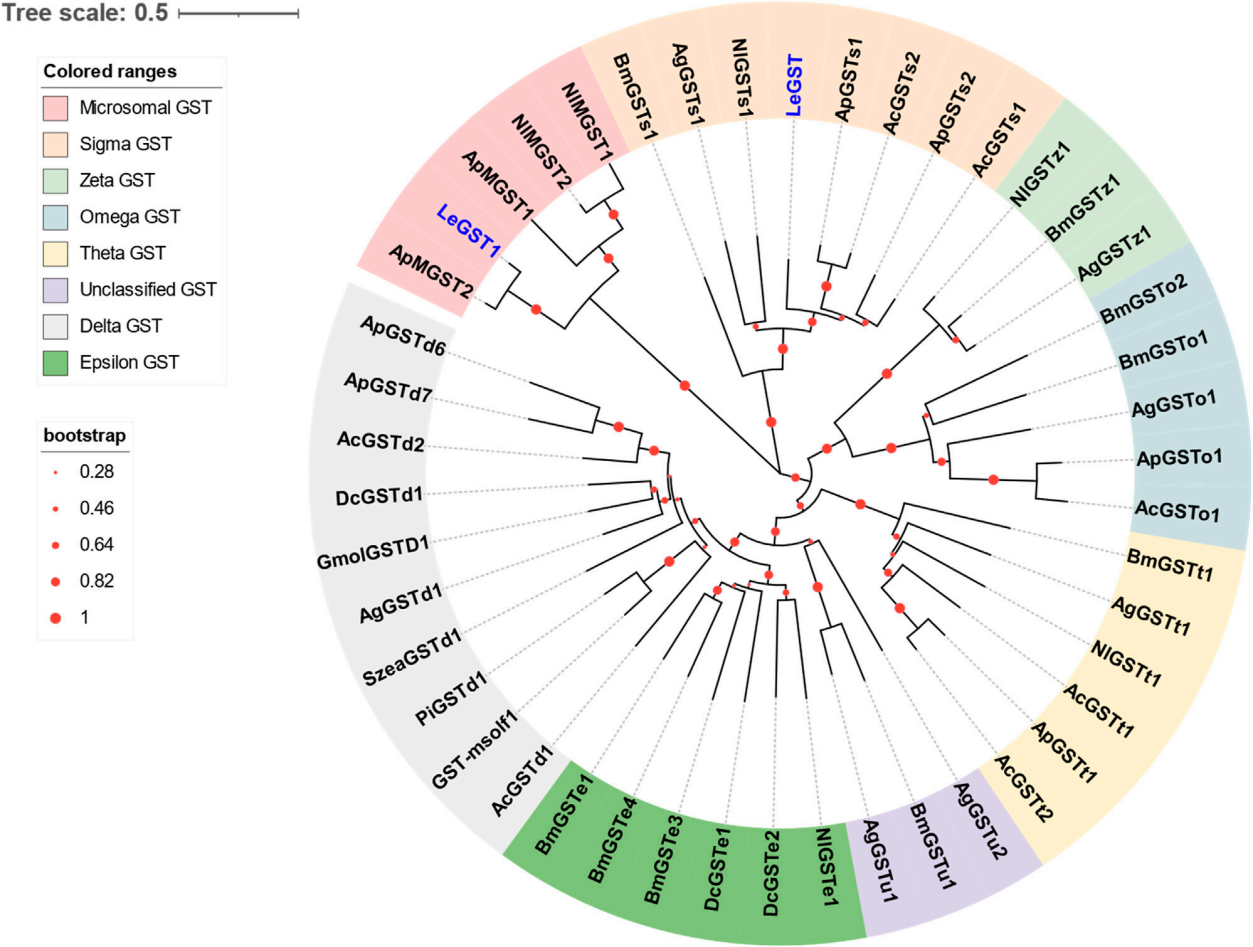
Phylogenetic relationship of 46 GSTs from hemipteran insect *L. erysimi* (Le, 2), *Aphis craccivora* (Ac, 7), *A. pisum* (Ap, 8), *D. citri* (Dc, 3), and *Nilaparvata lugens* (Nl, 6); and 16 reported GSTs from *Anopheles gambiae* (Ag, 7) and *Bombyx mori* (Bm, 9), as well as the antennal GmolGSTD1 from *Grapholita molesta*, SzeaGSTd1 from *Sitophilus zeamais*, PiGSTd1 from *Plodia interpunctella*, and GST-msolf1 from *Manduca sexta*. The neighbor-joining (NJ) tree was constructed using MEGA 11 with 1,000 bootstrap replicates. Two *L. erysimi* GSTs are highlighted in blue. The sequences used in this tree are provided in Supplementary Table S3.

Two DEGs abundant in antennae were identified to be GSTs. Both *LeGST1* and *LeGST* transcripts had full-length ORFs, encoding proteins that are 157 and 198 amino acids, respectively. It is noteworthy that *LeGST1* showed an expression pattern that was particularly abundant in antennae, with an FPKM value of 93.81 that exceeded 18-fold higher than in the body (Table 2). Conserved domain analysis showed LeGST1 had a MAPEG (membrane-associated proteins in eicosanoid and glutathione metabolism) domain and was similar to the microsomal GST1, while LeGST had one GSH binding site (G-site) in the N-terminus and one hydrophobic substrate binding pocket (H-site) in the C-terminal region (Supplementary Figure S2). The phylogenetic analysis revealed that eight subclasses, namely, Microsomal-, Delta-, Epsilon-, Omega-, Sigma-, Theta-, Zeta-, and the unclassified-GST, were well clustered in their respective phylogenetic branches. LeGST1 was classified under the Microsomal-GST subclass, and LeGST was classified as a member of Sigma-GST (Figure 4).

### 3.5 Identification of putative UGT genes

**FIGURE 5:**
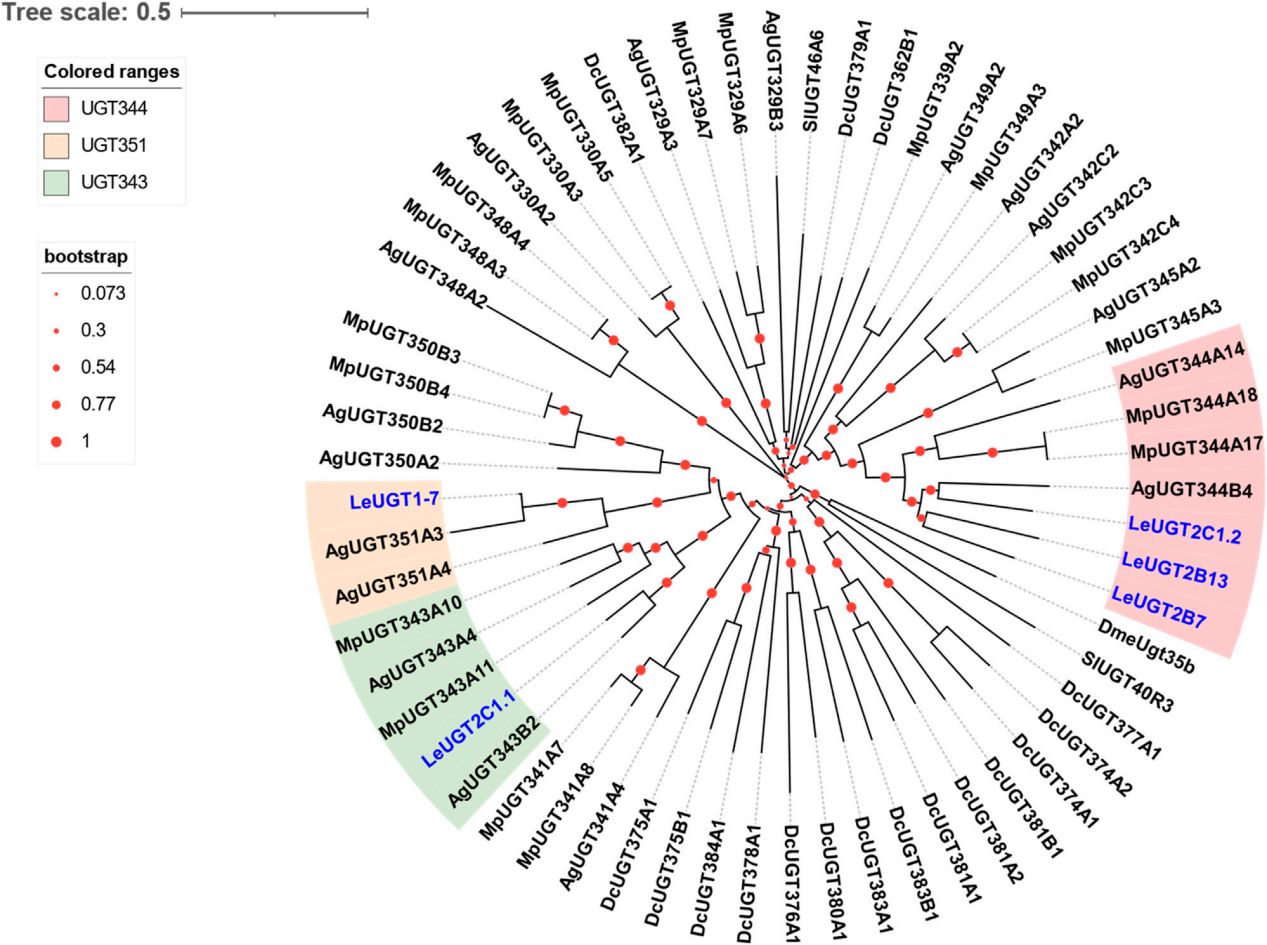
Phylogenetic relationship of 61 UGTs from the aphid species *L. erysimi* (Le, 5), *M. persicae* (Mp, 19), *Aphis gossypii* (Ag, 17), and the hemipteran insect *D. citri* (Dc, 17); as well as the reported antennal SlUGT40R3 and SlUGT46A6 from *Spodoptera littoralis*, and DmeUgt35b from *Drosophila melanogaster*. The neighbor-joining (NJ) tree was constructed using MEGA 11 with 1,000 bootstrap replicates. Five *L. erysimi* UGTs identified in this study are highlighted in blue. The sequences used in this tree are provided in Supplementary Table S4.

A total of five antennal *LeUGT* genes were identified in the utilized transcript set. All *LeUGT* transcripts had full-length ORFs, encoding proteins ranging from 513 to 542 amino acids. Signal peptides were predicted in all candidate LeUGTs, with the exception of *LeUGT2B13*. FPKM analysis showed that *LeUGT2B7* was the most antennae-abundant UGT with a value of 404.61, which was >20-fold higher than in the body (Table 1). Multiple alignments revealed the UGT motif signature sequence, (FVA)-(LIVMF)-(TS)-(HQ)-(SGAC)-G-X (2)-(STG)-X (2)-(DE)-X (6)-P-(LIVMFA)-(LIVMFA)-X (2)-P-(LMVFIQ)-X (2)-(DE)-Q, was situated at the C-terminus of LeUGTs (Supplementary Figure S3). Phylogenetic analysis showed that the candidate LeUGTs were grouped into three distinct subclades, with each subclade including several homologs from other aphid species. Specifically, LeUGT2B7, LeUGT2B13, and LeUGT2C1.2 were categorized into the UGT344 clade; LeUGT2C1.1 was clustered in the UGT343 subclade, and LeUGT1-7 was found to be a member of the UGT351 subclade (Figure 5).

### 3.6 Developmental and tissue expression analysis for candidate ODE genes

**FIGURE 6:**
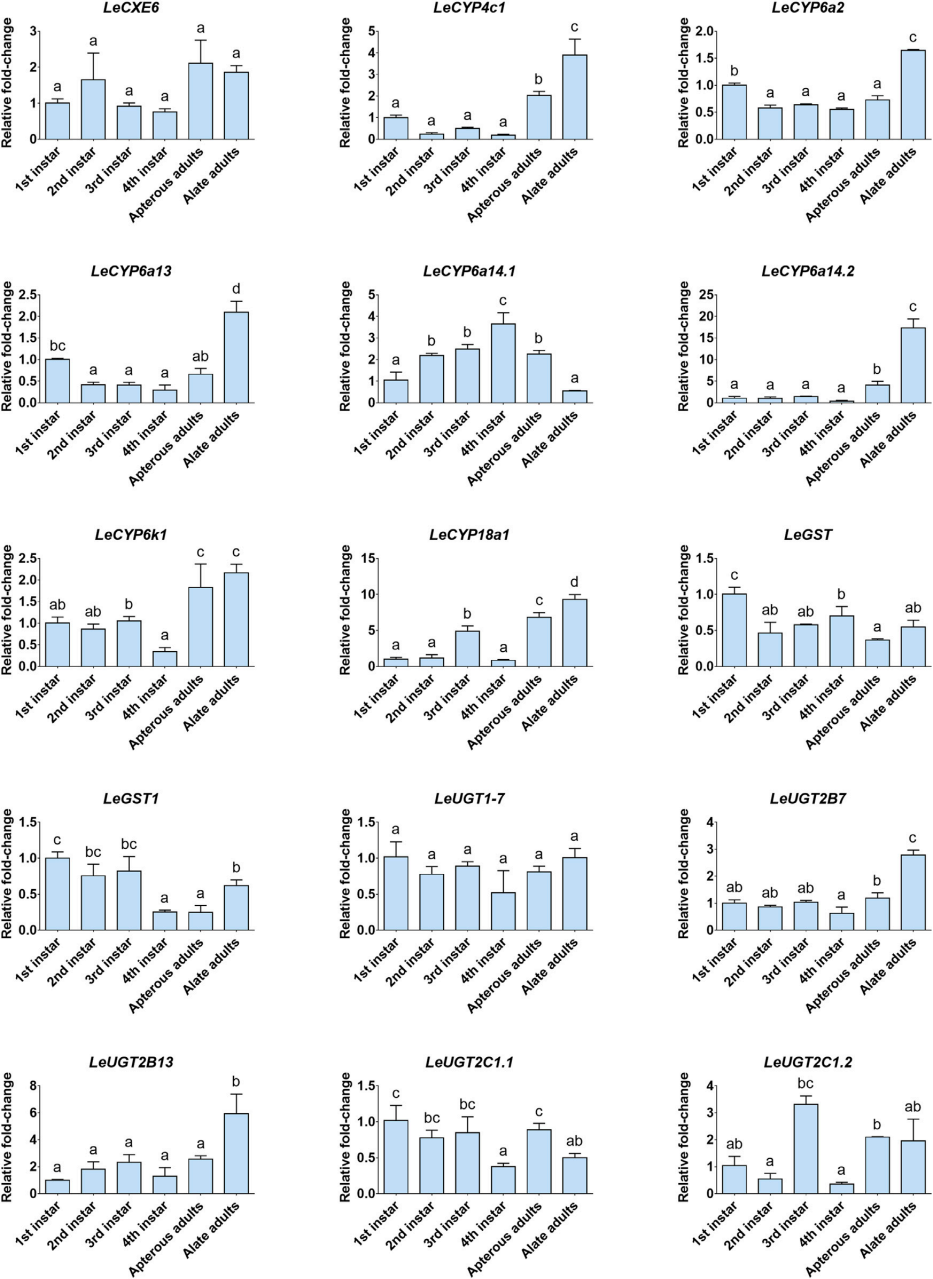
The relative expression levels of candidate odorant degrading enzyme (ODE) genes among different developmental stages of *L. erysimi*. The expression level of the first instar nymph was arbitrarily assigned a value of 1. Different lowercase letters above the error bar indicate statistically significant differences among aphid developmental stages (*p* < 0.05; one-way ANOVA, Tukey’s multiple comparisons test).

**FIGURE 7:**
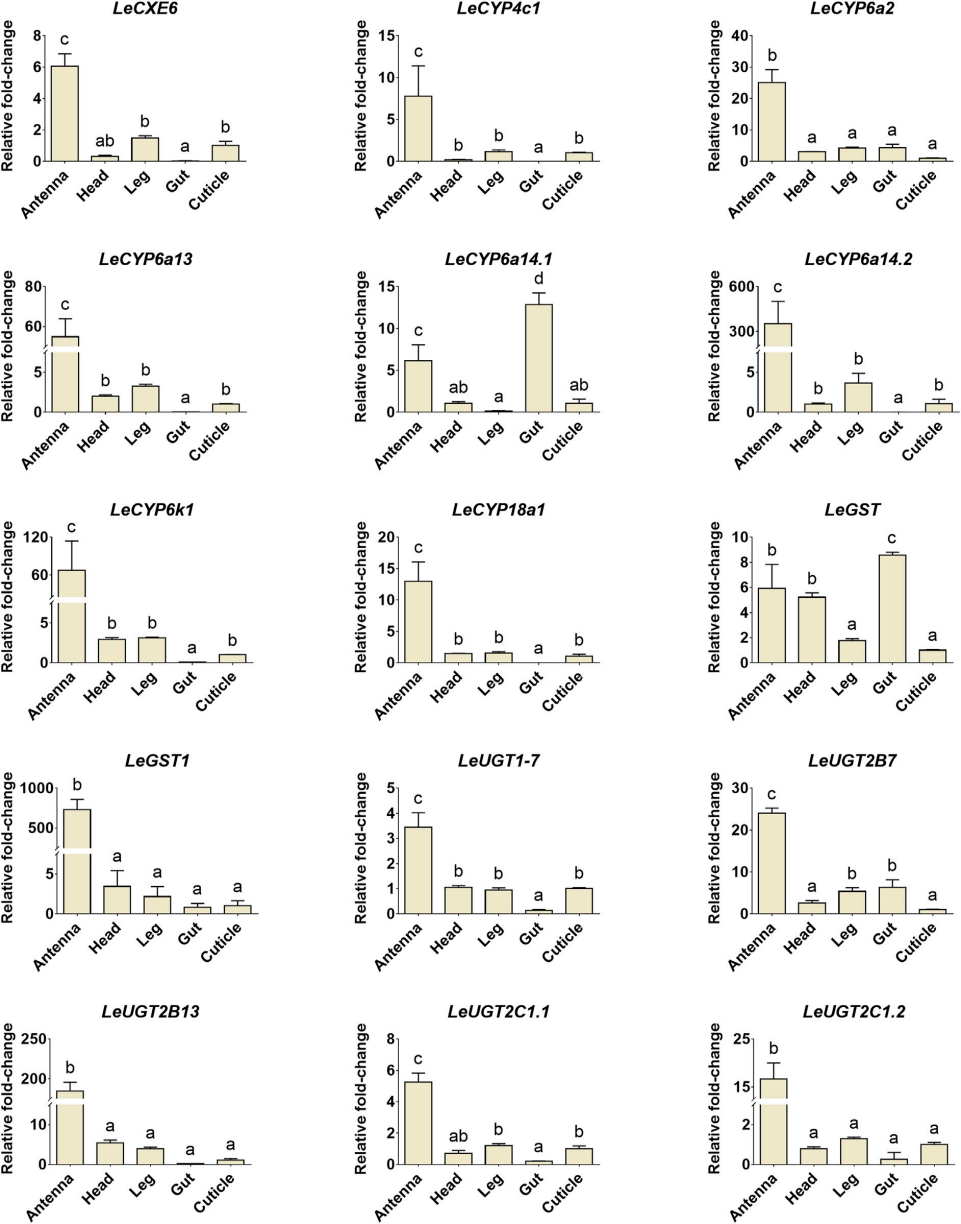
The relative expression levels of candidate ODE genes in different tissues of *L. erysimi*. The expression level in cuticle was arbitrarily given a value of 1, and the expression levels in other tissues were presented relative to the average cuticle. Significant differences of the relative abundance among aphid tissues were indicated by different letters above the error bar (*p* < 0.05; one-way ANOVA, Tukey’s multiple comparisons test).

The developmental and tissue expression profiles of *LeCXE*, *LeCYP*, *LeGST*, and *LeUGT* genes were analyzed using qRT-PCR. Developmental expression data showed that the candidate ODE genes were consistently detected throughout the various developmental stages of *L. erysimi*, spanning from the first instar nymph to the adult stage. Notably, *LeCYP4c1*, *LeCYP6a2*, *LeCYP6a13*, *LeCYP6a14.2*, *LeCYP18a1*, *LeUGT2B7*, and *LeUGT2B13* exhibited significantly higher expression levels in alate aphids compared to apterous and nymph aphids (Figure 6). Tissue expression analysis revealed that *LeCYP6a14.1* and *LeGST* were highly expressed in both antenna and gut tissue, while the remaining 13 ODE genes displayed antennae-enriched expression profiles. In particular, the antennal expression levels of *LeCYP6a13*, *LeCYP6k1*, *LeCYP6a14.2*, *LeGST1*, *LeUGT2B13*, and *LeUGT2C1.2* were >10-fold higher than in other tissues; *LeCXE6*, *LeCYP4c1*, *LeCYP6a2*, *LeCYP18a1*, *LeUGT2B7*, and *LeUGT2C1.1* exhibited more than four times higher expression in antennae compared to non-olfactory tissues such as the head, leg, gut, and cuticle (Figure 7).

## 4 Discussion

Insects depend on their antennae to detect and process hydrophobic odorant molecules (Krieger and Breer, 1999; Leal, 2013). Discovering the ODEs within the antennae would provide crucial insights into the odorant recognition mechanism of *L. erysimi*, which may help us control this destructive agricultural pest more effectively. In this study, comparing the transcriptome data of the antennal and body tissues identified one CXE, seven CYPs, two GSTs, and five UGTs. The developmental and tissue expression profiles of these ODE genes were determined to reveal their implications in odorant degradation during the process of olfactory perception. To our knowledge, this is the first report documenting the identification of ODE genes in this aphid species.

The widespread occurrence of CXEs enables a tremendous decrease in the concentration of ester compounds in insects. This leads to improved sensitivity of the olfactory system and minimizes the possible toxic impact of these compounds. Several antennae abundant CXEs have been functionally studied and confirmed as ODEs, and employed for the purpose of eliminating odorants in the antennae (Ishida and Leal, 2005; 2008; Durand et al., 2010; Durand et al., 2011; He et al., 2015; Wei et al., 2021). For example, two CXEs, *SlCXE7* and *SlCXE10*, are predominantly expressed in the antennal sensilla, and play a key role in the degradation of pheromones and plant volatile components in the cotton leafworm, *S. littoralis* (Durand et al., 2010; Durand et al., 2011). A similar study in *G. molesta* has uncovered four antenna-enriched CXEs play a crucial role in regulating the insect’s foraging and mating behaviors. Specifically, GmolCXE1 and GmolCXE5 are responsible for hydrolyzing the acetate sex pheromone (Z/E)-8-dodecenyl, while GmolCXE14 and GmolCXE21 are involved in metabolizing the ester host plant volatiles ethyl butanoate and ethyl hexanoate (Wei et al., 2021). In our study, combined transcriptome and qRT-PCR analysis revealed that LeCXE6 was highly enriched in the antennae. Further phylogenetic analysis indicated that LeCXE6 was grouped into the “beta esterases” clade along with two well-characterized pheromone-degrading enzymes, ApolPDE of *A. polyphemus* and PjapPDE of *P. japonica* (Ishida and Leal, 2005; 2008). These findings suggest that LeCXE6 may play a significant role in clearing redundant odorants during chemosensory processing.

CYPs represent an essential family of detoxification enzymes that widely occur in both vertebrates and invertebrates. Accumulating studies have shown that insect CYPs, especially those found abundantly in antennae, play a significant role as ODEs in the metabolism of host plant volatiles and sex pheromones (Chiu et al., 2019a; Chiu et al., 2019b; Chiu et al., 2019c; Wu et al., 2022). In this study, a total of seven antennae enriched *LeCYP* genes were identified. Our number of antennal CYP genes in *L. erysimi* is comparable to those found in other insect species, such as seven antennae-abundant CYPs were documented in *D. citri* (Kuang et al., 2022), as well as four CYPs (CYP4L4, CYP4S4, CYP9A13, and CYP4G20) of *Mamestra brassicae* and four CYPs (CYP6DE1, CYP6DJ1, CYP6BW1, and CYP6BW3) of *D. ponderosae* have been found to be highly expressed in the antennae (Maïbèche-Coisne et al., 2002; Maïbèche-Coisne et al., 2005; Chiu et al., 2019a; Chiu et al., 2019b; Chiu et al., 2019c). Insect P450 genes are commonly divided into four clades, which include CYP2, CYP3, CYP4, and the mitochondrial CYP. Herein, we found five LeCYPs (i.e., LeCYP6a13, LeCYP6a2, LeCYP6k1, LeCYP6a14.1, and LeCYP6a14.2) were grouped into the CYP3 clan. Recent research has demonstrated that many members of the CYP3 clan played an important role in facilitating herbivore adaptation to their host plants. For instance, two CYP3 genes (*DponCYP345E2* and *DponCYP6DE1*) of *D. ponderosae*, were reported to catalyze the oxidation of monoterpene pine host volatiles such as α-pinene (Keeling et al., 2013; Chiu et al., 2019a), and the enhanced expression of CYP3 P450 genes has been observed in *Dendroctonus armandi* in response to host terpenoids such as pinene, 3-carene, and limonene (Dai et al., 2016).

Several GSTs, UGTs, and AOX enzymes expressed in insect antennae have been suggested to play crucial roles in the decomposition of odorous compounds. For instance, *GST-msolf1* of *M. sexta* (Rogers et al., 1999), *GmolGSTD1* of *G. molesta* (Li et al., 2018), *PiGSTd1* of *Plodia interpunctella* (Liu et al., 2021), *UGT36E1* of *Drosophila melanogaster* (Fraichard et al., 2020), and *PxylAOX3* of *P. xylostella* (Wang et al., 2021a) have been implicated in this process. In our investigation, *LeGST1* along with four UGTs (*LeUGT2B7*, *LeUGT2B13*, *LeUGT2C1.1*, and *LeUGT2C1.2*) displayed antennae-enriched expression profiles. However, no enrichment of any AOX genes was observed in the antennae of *L. erysimi*. Meanwhile, developmental expression analysis showed that *LeCYP4c1*, *LeCYP6a2*, *LeCYP6a13*, *LeCYP6a14.2*, *LeCYP18a1*, *LeUGT2B7*, and *LeUGT2B13* exhibited significantly higher expression levels in alate aphids when compared to apterous and nymph aphids. Given that alate aphids often encountered complex surroundings consisting of a variety of odorants while navigating in search of new host plants, elevated levels of these ODEs may aid in maintaining their olfactory sensitivity.

In summary, this study has identified a dataset of CXE, CYP, GST, and UGT genes from *L. erysimi*, which might be involved in the processes of pheromone and/or plant volatile degradation. Previous studies have found that antennae-enriched ODEs offer great promise in the development of behavioral interference control strategies, in which ODE-silenced insects are expected to exhibit decreased or disordered foraging behaviors (Yu et al., 2016; Wei et al., 2021; Wu et al., 2022; Ma et al., 2023). Examples include RNAi of *LmCYP6FD5*, an antennae-specific P450 gene of *Locusta migratoria*, the EAG responses of locusts to the main volatiles of gramineous plants, including trans-2-Hexen-1-al, cis-3-Hexenyl acetate, and decanal, were significantly diminished (Wu et al., 2022). Therefore, future research on the physiological role of these ODE genes will pave the way toward understanding the olfactory mechanism of *L. erysimi*, and provide new targets for developing behavioral interference control strategies (e.g., RNAi) against this insect pest.

## Data Availability

The datasets presented in this study can be found in online repositories. The names of the repository/repositories and accession number(s) can be found in the article/Supplementary Material.

## References

[B1] Blomquist G. J. Tittiger C. MacLean M. Keeling C. I. (2021). Cytochromes P450: Terpene detoxification and pheromone production in bark beetles. Curr. Opin. Insect Sci. 43, 97–102. 10.1016/j.cois.2020.11.010 33359166

[B2] Bozzolan F. Siaussat D. Maria A. Durand N. Pottier M. A. Chertemps T. (2014). Antennal uridine diphosphate (UDP)‐glycosyltransferases in a pest insect: Diversity and putative function in odorant and xenobiotics clearance. Insect Mol. Biol. 23, 539–549. 10.1111/imb.12100 24698447

[B3] Cheema J. A. Carraher C. Plank N. O. Travas-Sejdic J. Kralicek A. (2021). Insect odorant receptor-based biosensors: Current status and prospects. Biotechnol. Adv. 53, 107840. 10.1016/j.biotechadv.2021.107840 34606949

[B4] Chertemps T. Meïbèche M. (2021). “19 - odor degrading enzymes and signal termination,” in Insect pheromone biochemistry and molecular biology. Editors Gary J. Blomquist Richard G. Vogt. 2nd Edn (Cambridge, MA: Academic Press), 619–644.

[B5] Chiu C. C. Keeling C. I. Bohlmann J. (2019b). Cytochromes P450 preferentially expressed in antennae of the mountain pine beetle. J. Chem. Ecol. 45, 178–186. 10.1007/s10886-018-0999-0 30043088

[B6] Chiu C. C. Keeling C. I. Bohlmann J. (2019a). The cytochrome P450 CYP6DE1 catalyzes the conversion of α-pinene into the mountain pine beetle aggregation pheromone trans-verbenol. Sci. Rep. 9, 1477–1510. 10.1038/s41598-018-38047-8 30728428 PMC6365528

[B7] Chiu C. C. Keeling C. I. Henderson H. M. Bohlmann J. (2019c). Functions of mountain pine beetle cytochromes P450 CYP6DJ1, CYP6BW1 and CYP6BW3 in the oxidation of pine monoterpenes and diterpene resin acids. PLoS One 14, e0216753. 10.1371/journal.pone.0216753 31071168 PMC6508646

[B8] Dai L. Ma M. Gao G. Chen H. (2016). *Dendroctonus armandi* (Curculionidae: Scolytinae) cytochrome P450s display tissue specificity and responses to host terpenoids. Comp. Biochem. Physiol. B Biochem. Mol. Biol. 201, 1–11. 10.1016/j.cbpb.2016.06.006 27344973

[B9] Ding Q. Xu X. Sang Z. Wang R. Ullah F. Gao X. (2022). Characterization of the insecticide detoxification carboxylesterase Boest1 from *Bradysia odoriphaga* Yang et Zhang (Diptera: Sciaridae). Pest Manag. Sci. 78, 591–602. 10.1002/ps.6667 34596943

[B10] Ding Y. Ortelli F. Rossiter L. C. Hemingway J. Ranson H. (2003). The *Anopheles gambiae* glutathione transferase supergene family: Annotation, phylogeny and expression profiles. BMC Genom 4, 35–16. 10.1186/1471-2164-4-35 PMC194574 12914673

[B11] Durand N. Carot-Sans G. Bozzolan F. Rosell G. Siaussat D. Debernard S. (2011). Degradation of pheromone and plant volatile components by a same odorant-degrading enzyme in the cotton leafworm, *Spodoptera littoralis* . PLoS One 6, e29147. 10.1371/journal.pone.0029147 22216190 PMC3246455

[B12] Durand N. Carot-Sans G. Chertemps T. Bozzolan F. Party V. Renou M. (2010). Characterization of an antennal carboxylesterase from the pest moth *Spodoptera littoralis* degrading a host plant odorant. PLoS One 5, e15026. 10.1371/journal.pone.0015026 21124773 PMC2993938

[B13] Fraichard S. Legendre A. Lucas P. Chauvel I. Faure P. Neiers F. (2020). Modulation of sex pheromone discrimination by a UDP-glycosyltransferase in *Drosophila melanogaster* . Genes 11, 237. 10.3390/genes11030237 32106439 PMC7140800

[B14] He P. Zhang Y. N. Yang K. Li Z. Q. Dong S. L. (2015). An antenna-biased carboxylesterase is specifically active to plant volatiles in *Spodoptera exigua* . Pestic. Biochem. Physiol. 123, 93–100. 10.1016/j.pestbp.2015.03.009 26267057

[B15] Ishida Y. Leal W. S. (2008). Chiral discrimination of the Japanese beetle sex pheromone and a behavioral antagonist by a pheromone-degrading enzyme. Proc. Natl. Acad. Sci. U. S. A. 105, 9076–9080. 10.1073/pnas.0802610105 18579770 PMC2440356

[B16] Ishida Y. Leal W. S. (2005). Rapid inactivation of a moth pheromone. Proc. Natl. Acad. Sci. U. S. A. 102, 14075–14079. 10.1073/pnas.0505340102 16172410 PMC1216831

[B17] Keeling C. I. Henderson H. Li M. Dullat H. K. Ohnishi T. Bohlmann J. (2013). CYP345E2, an antenna-specific cytochrome P450 from the mountain pine beetle, *Dendroctonus ponderosae* Hopkins, catalyses the oxidation of pine host monoterpene volatiles. Insect biochem. Mol. Biol. 43, 1142–1151. 10.1016/j.ibmb.2013.10.001 24139909

[B18] Krieger J. Breer H. (1999). Olfactory reception in invertebrates. Science 286, 720–723. 10.1126/science.286.5440.720 10531050

[B19] Kuang Y. Shangguan C. Yuan S. Zhang Q. Qiu Z. Gao L. (2023). Candidate odorant-binding protein and chemosensory protein genes in the turnip aphid *Lipaphis erysimi* . Arch. Insect Biochem. Physiol., e22022. 10.1002/arch.22022 37154128

[B20] Kuang Y. Xiong Y. Chen X. D. Yu X. (2022). Antennae-abundant expression of candidate cytochrome P450 genes associated with odorant degradation in the Asian citrus psyllid, *Diaphorina citri* . Front. Physiol. 13, 1004192. 10.3389/fphys.2022.1004192 36176776 PMC9513247

[B21] Leal W. S. (2013). Odorant reception in insects: Roles of receptors, binding proteins, and degrading enzymes. Annu. Rev. Entomol. 58, 373–391. 10.1146/annurev-ento-120811-153635 23020622

[B22] Li G. W. Chen X. L. Xu X. L. Wu J. X. (2018). Degradation of sex pheromone and plant volatile components by an antennal glutathione S‐transferase in the oriental fruit moth, Grapholita molesta Busck (Lepidoptera: Tortricidae). Arch. Insect Biochem. Physiol. 99, e21512. 10.1002/arch.21512 30387866

[B23] Liu H. Tang Y. Wang Q. Shi H. Yin J. Li C. (2021). Identification and characterization of an antennae-specific glutathione S-transferase from the Indian meal moth. Front. Physiol. 12, 727619. 10.3389/fphys.2021.727619 34512396 PMC8427598

[B24] Livak K. J. Schmittgen T. D. (2001). Analysis of relative gene expression data using real-time quantitative PCR and the 2^−ΔΔCT^ method. Methods 25, 402–408. 10.1006/meth.2001.1262 11846609

[B25] Ma Y. F. Gong L. L. Zhang M. Q. Liu X. Z. Guo H. Hull J. J. (2023). Two antenna-enriched carboxylesterases mediate olfactory responses and degradation of ester volatiles in the German cockroach *Blattella germanica* . J. Agric. Food Chem. 71, 4789–4801. 10.1021/acs.jafc.2c08488 36920281

[B26] Maïbèche-Coisne M. Merlin C. François M. C. Porcheron P. Jacquin-Joly E. (2005). P450 and P450 reductase cDNAs from the moth *Mamestra brassicae*: Cloning and expression patterns in male antennae. Gene 346, 195–203. 10.1016/j.gene.2004.11.010 15716002

[B27] Maïbèche‐Coisne M. Jacquin‐Joly E. François M. C. Nagnan‐Le Meillour P. (2002). cDNA cloning of biotransformation enzymes belonging to the cytochrome P450 family in the antennae of the noctuid moth *Mamestra brassicae* . Insect Mol. Biol. 11, 273–281. 10.1046/j.1365-2583.2002.00335.x 12000647

[B28] Pan Y. Tian F. Wei X. Wu Y. Gao X. Xi J. (2018). Thiamethoxam resistance in *Aphis gossypii* glover relies on multiple UDP-glucuronosyltransferases. Front. Physiol. 9, 322. 10.3389/fphys.2018.00322 29670540 PMC5893893

[B29] Pan Y. Xu P. Zeng X. Liu X. Shang Q. (2019). Characterization of UDP-glucuronosyltransferases and the potential contribution to nicotine tolerance in *Myzus persicae* . Int. J. Mol. Sci. 20, 3637. 10.3390/ijms20153637 31349586 PMC6695686

[B30] Pelosi P. Iovinella I. Zhu J. Wang G. Dani F. R. (2018). Beyond chemoreception: Diverse tasks of soluble olfactory proteins in insects. Biol. Rev. 93, 184–200. 10.1111/brv.12339 28480618

[B31] Ramsey J. S. Rider D. S. Walsh T. K. De Vos M. Gordon K. Ponnala L. (2010). Comparative analysis of detoxification enzymes in *Acyrthosiphon pisum* and *Myzus persicae* . Insect Mol. Biol. 19, 155–164. 10.1111/j.1365-2583.2009.00973.x 20482647

[B32] Rogers M. E. Jani M. K. Vogt R. G. (1999). An olfactory-specific glutathione-S-transferase in the sphinx moth *Manduca sexta* . J. Exp. Biol. 202, 1625–1637. 10.1242/jeb.202.12.1625 10333508

[B33] Tamura K. Stecher G. Kumar S. (2021). MEGA11: Molecular evolutionary genetics analysis version 11. Mol. Biol. Evol. 38, 3022–3027. 10.1093/molbev/msab120 33892491 PMC8233496

[B34] Tian F. Wang Z. Li C. Liu J. Zeng X. (2019). UDP-Glycosyltransferases are involved in imidacloprid resistance in the Asian citrus psyllid, *Diaphorina citri* (Hemiptera: Lividae). Pestic. Biochem. Physiol. 154, 23–31. 10.1016/j.pestbp.2018.12.010 30765053

[B35] Vogt R. G. Riddiford L. M. (1981). Pheromone binding and inactivation by moth antennae. Nature 293, 161–163. 10.1038/293161a0 18074618

[B36] Vogt R. G. Riddiford L. M. Prestwich G. D. (1985). Kinetic properties of a sex pheromone-degrading enzyme: The sensillar esterase of *Antheraea polyphemus* . Proc. Natl. Acad. Sci. U. S. A. 82, 8827–8831. 10.1073/pnas.82.24.8827 3001718 PMC391531

[B37] Vontas J. G. Small G. J. Nikou D. C. Ranson H. Hemingway J. (2002). Purification, molecular cloning and heterologous expression of a glutathione S-transferase involved in insecticide resistance from the rice Brown planthopper, *Nilaparvata lugens* . Biochem. J. 362, 329–337. 10.1042/0264-6021:3620329 11853540 PMC1222392

[B38] Wang M. M. He M. Wang H. Ma Y. F. Dewer Y. Zhang F. (2021a). A candidate aldehyde oxidase in the antennae of the diamondback moth, *Plutella xylostella* (L), is potentially involved in the degradation of pheromones, plant-derived volatiles and the detoxification of xenobiotics. Pestic. Biochem. Physiol. 171, 104726. 10.1016/j.pestbp.2020.104726 33357547

[B39] Wang M. M. Long G. J. Guo H. Liu X. Z. Wang H. Dewer Y. (2021b). Two carboxylesterase genes in *Plutella xylostella* associated with sex pheromones and plant volatiles degradation. Pest Manag. Sci. 77, 2737–2746. 10.1002/ps.6302 33527628

[B40] Wang Q. Hasan G. Pikielny C. W. (1999). Preferential expression of biotransformation enzymes in the olfactory organs of *Drosophila melanogaster*, the antennae. J. Biol. Chem. 274, 10309–10315. 10.1074/jbc.274.15.10309 10187818

[B41] Wei H. Tan S. Li Z. Li J. Moural T. W. Zhu F. (2021). Odorant degrading carboxylesterases modulate foraging and mating behaviors of *Grapholita molesta* . Chemosphere 270, 128647. 10.1016/j.chemosphere.2020.128647 33757271

[B42] Wu H. Liu J. Liu Y. Abbas M. Zhang Y. Kong W. (2022). CYP6FD5, an antenna-specific P450 gene, is potentially involved in the host plant recognition in *Locusta migratoria* . Pestic. Biochem. Physiol. 188, 105255. 10.1016/j.pestbp.2022.105255 36464360

[B43] Wu Z. Pu X. Shu B. Bin S. Lin J. (2020). Transcriptome analysis of putative detoxification genes in the Asian citrus psyllid, *Diaphorina citri* . Pest Manag. Sci. 76, 3857–3870. 10.1002/ps.5937 32483911

[B44] Xia D. Zheng R. Huang J. Lu S. Tang Q. (2022). Identification and functional analysis of glutathione S-transferases from *Sitophilus zeamais* in olfactory organ. Insects 13, 259. 10.3390/insects13030259 35323557 PMC8950995

[B45] Yang Y. X. Lin R. H. Li Z. Wang A. Y. Xue C. Duan A. L. (2021). Function analysis of P450 and GST genes to imidacloprid in *Aphis craccivora* (Koch). Front. Physiol. 11, 624287. 10.3389/fphys.2020.624287 33551847 PMC7854575

[B46] Younus F. Chertemps T. Pearce S. L. Pandey G. Bozzolan F. Coppin C. W. (2014). Identification of candidate odorant degrading gene/enzyme systems in the antennal transcriptome of *Drosophila melanogaster* . Insect biochem. Mol. Biol. 53, 30–43. 10.1016/j.ibmb.2014.07.003 25038463

[B47] Yu Q. Lu C. Li B. Fang S. Zuo W. Dai F. (2008). Identification, genomic organization and expression pattern of glutathione S-transferase in the silkworm, *Bombyx mori* . Insect biochem. Mol. Biol. 38, 1158–1164. 10.1016/j.ibmb.2008.08.002 19280710

[B48] Yu X. D. Liu Z. C. Huang S. L. Chen Z. Q. Sun Y. W. Duan P. F. (2016). RNAi‐mediated plant protection against aphids. Pest Manag. Sci. 72, 1090–1098. 10.1002/ps.4258 26888776

[B49] Yu X. Killiny N. (2018). RNA interference of two glutathione S‐transferase genes, *Diaphorina citri* DcGSTe2 and DcGSTd1, increases the susceptibility of Asian citrus psyllid (Hemiptera: Liviidae) to the pesticides fenpropathrin and thiamethoxam. Pest Manag. Sci. 74, 638–647. 10.1002/ps.4747 28971568

[B50] Yu X. Marshall H. Liu Y. Xiong Y. Zeng X. Yu H. (2023). Sex-specific transcription and DNA methylation landscapes of the Asian citrus psyllid, a vector of huanglongbing pathogens. Evolution 77, 1203–1215. 10.1093/evolut/qpad036 36869727

[B51] Yu X. Wang G. Huang S. Ma Y. Xia L. (2014). Engineering plants for aphid resistance: Current status and future perspectives. Theor. Appl. Genet. 127, 2065–2083. 10.1007/s00122-014-2371-2 25151153

[B52] Zhou S. Jander G. (2022). Molecular ecology of plant volatiles in interactions with insect herbivores. J. Exp. Bot. 73, 449–462. 10.1093/jxb/erab413 34581787

[B53] Zhou W. W. Liang Q. M. Xu Y. Gurr G. M. Bao Y. Y. Zhou X. P. (2013). Genomic insights into the glutathione S-transferase gene family of two rice planthoppers, *Nilaparvata lugens* (stål) and *Sogatella furcifera* (horváth) (Hemiptera: Delphacidae). PLoS One 8, e56604. 10.1371/journal.pone.0056604 23457591 PMC3572974

