# Supplementary material for: Antennae-enriched expression of candidate odorant degrading enzyme genes in the turnip aphid, Lipaphis erysimi
Source: Front Physiol. 2023 Jul 5;14:1228570. doi: 10.3389/fphys.2023.1228570 (PMC10354451; doi:10.3389/fphys.2023.1228570)
Supplement: Supplementary file 1 [file DataSheet1.docx]

Supplementary Material


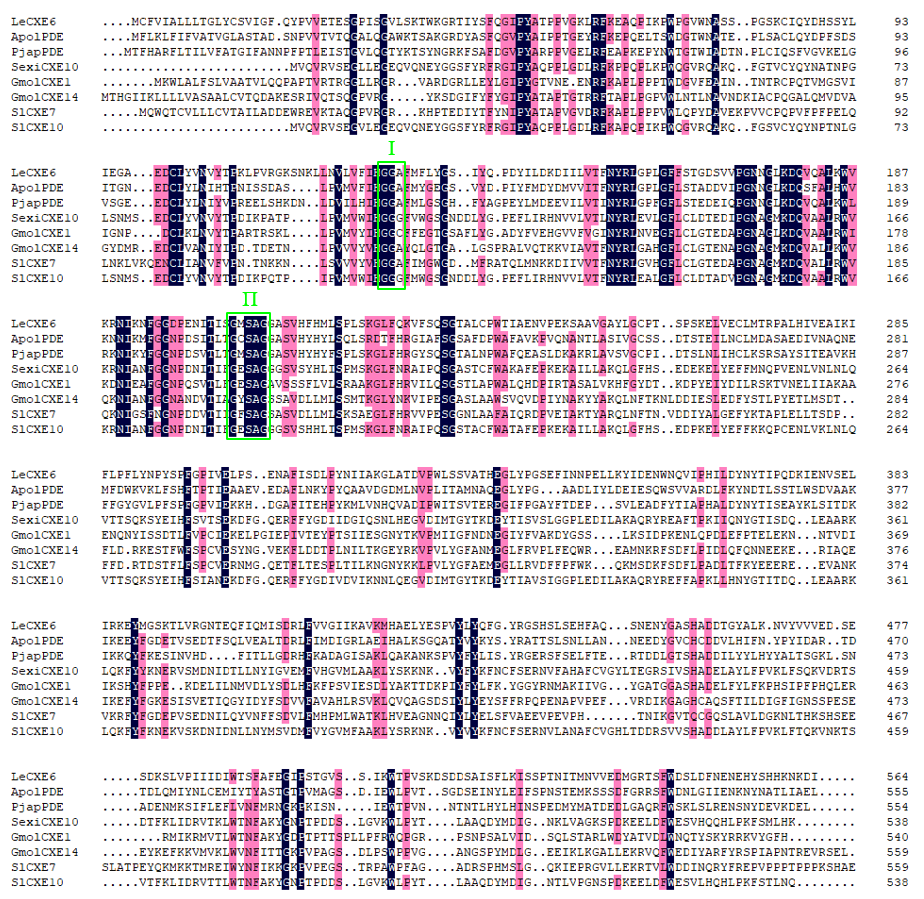


**Figure S1.** Comparison of the deduced amino acid sequences of LeCXE6 with the representative CXEs from *Grapholita molesta* (GmolCXE1 and GmolCXE14), *Spodoptera littoralis* (SlCXE7 and SlCXE10), *Spodoptera exigua* (SexiCXE10), *Antheraea polyphemus* (ApolPDE), and *Popillia japonica* (PjapPDE). Letters shaded in dark blue represent the consensus residues, and the residues of ≥ 75% identities among CXEs are shaded in pink. The conserved oxyanion hole is marked with box І, the pentapeptide Gly-X-Ser-X-Gly motif is marked with box П.


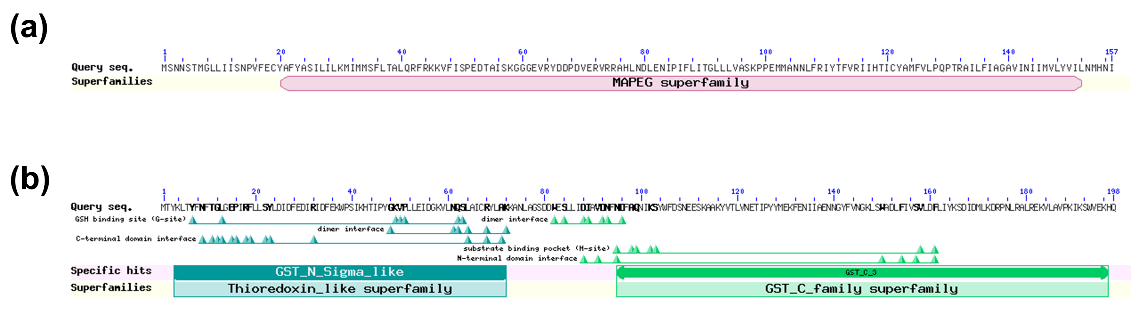


**Figure S2.** Conserved domain analysis of antennal LeGST. (a) The conserved domains of LeGST1 predicted by CDD-BLAST. (b) The conserved domains of LeGST predicted by CDD-BLAST.


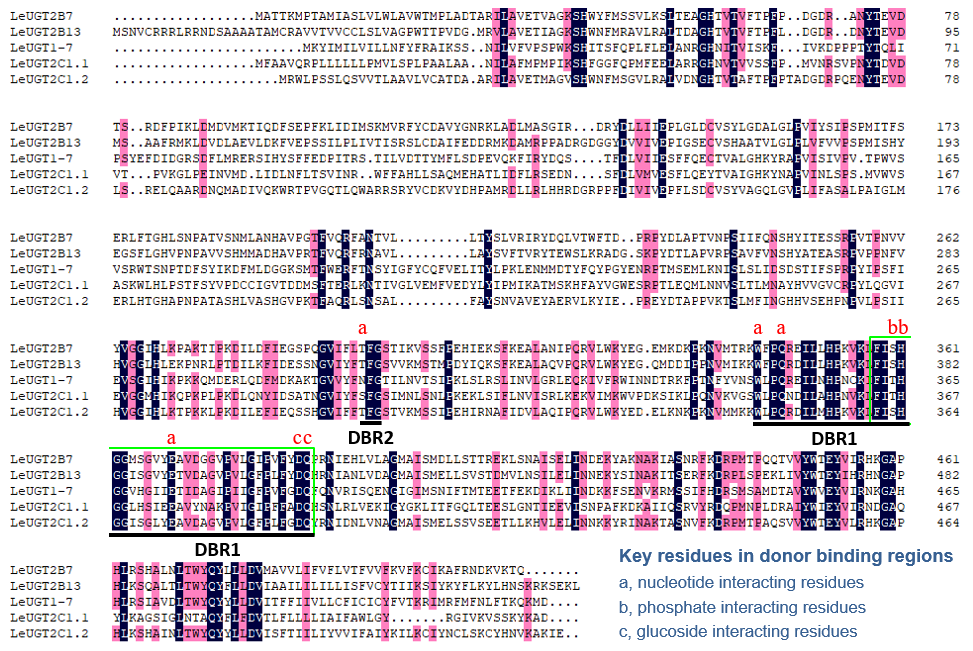


**Figure S3.** Multiple alignment of candidate UGTs in *L. erysimi*. The UGT signature motif has been highlighted with a green box. The donor binding regions (DBR) are represented with black bars located below the sequences. The key residues that interact with the sugar donor in DBR are marked with the letters (a, b, c) above the alignment.

**Table S1.** The amino acid sequences of 46 CXEs used in Figure 2

| **Protein name** | **Protein sequence** |
| --- | --- |
| LeCXE6 | MCFVIALLLTGLYCSVIGFQYPVVETESGPISGVLSKTWKGRTIYSFQGIPYATPPVGKLRFKEAQPIKPWPGVWNASSPGSKCIQYDHSSYLIEGAEDCLYVNVYTPKLPVRGKSNKLLNVLVFIHGGAFMFLYGSIYQPDYILDKDIILVTFNYRLGPLGFFSTGDSVVPGNNGLKDQVQALKWVKRNIKNFGGDPENITISGMSAGGASVHFHMLSPLSKGLFQKVFSQSGTALCPWTIAENVPEKSAAVGAYLGCPTSPSKELVECLMTRPALHIVEAIKIFLPFLYNPYSPFGPIVELPSENAFISDLPYNIIAKGLATDVPWLSSVATHEGLYPGSEFINNPELLKYIDENWNQVIPHILDYNYTIPQDKIENVSELIRKEYMGSKTLVRGNTEQFIQMISDRLFVVGIIKAVKMHAELYESPVYLYQFGYRGSHSLSEHFAQSNENYGASHADDTGYALKNVYVVVEDSESDKSLVPIIIDIWTSFAFEGIPSTGVSSIKWTPVSKDSDDSAISFLKISSPTNITMNVVEDMGRTSFWDSLDFNENEHYSHHKNKDI |
| SlCXE10 | MVQVRVSEGVLEGEQVQNEYGGSFYRFRGIPYAQPPLGDLRFKAPQPIKPWQGVRQAKQFGSVCYQYNPTNLGLSNMSEDCLYVNVYTPDIKPQTPIPVMVWIHGGGFMWGSGNDDLYGPEFLIRHNVVLVTFNYRLEALGFLCLDTADVPGNAGMKDQVAALRWVKRNIANFGGNPDNITIFGESAGGGSVSHHLISPMSKGLFNRAIPQSGSTACFWATAFEPKEKAILLAKQLGFHSEDPKELYEFFKKQPCENLVKLNLQVTTSQKSYEIHFSIANEKDFGQERFFYGDIVDVIKNNLQEGVDIMTGYTKDEYTIAVSIGGPLEDILAKAQRYREFFAPKLLHNYGTITDQLEAARKLQKFYFKNEKVSKDNIDNLLNYMSVDMFVYGVMFAAKLYSRKNKVYVYKFNCFSERNVLANAFCVGHLTDDRSVVSHADDLAYLFPVKLFTQKVNKTSVTFKLIDRVTTLWTNFAKYGNPTPDDSLGVKWLPYTLAAQDYMDIGNTLVPGNSPDKEELDFWESVLHQHLPKFSTLNQ |
| SexiCXE10 | MVQVRVSEGLLEGEQVQNEYGGSFYRFRGIPYAQPPLGDLRFKPPQPLKPWQGVRQAKQFGTVCYQYNATNPGLSNMSEDCLYVNVYTPDIKPATPLPVMVWIHGGGFVWGSGNDDLYGPEFLIRHNVVLVTLNYRLEVLGFLCLDTEDIPGNAGMKDQVAALRWVKRNIANFGGNPDNITIFGESAGGGSVSYHLISPMSKGLFNRAIPQSGASTCFWAKAFEPKEKAILLAKQLGFHSEDEKELYEFFMNQPVENLVNLNLQVTTSQKSYEIHFSVTSEKDFGQERFFYGDIIDGIQSNLHEGVDIMTGYTKDEYTISVSLGGPLEDILAKAQRYREAFTPKIIQNYGTISDQLEAARKLQKFYYKNERVSMDNIDTLLNYIGVEMFVHGVMLAAKLYSKKNKVYFYKFNCFSERNVFAHAFCVGYLTEGRSIVSHADELAYLFPVKLFSQKVDRTSDTFKLIDRVTKLWTNFAKYGNPTPDDSLGVKWLPYTLAAQDYMDIGNKLVAGKSPDKEELDFWESVHQQHLPKFSMLHK |
| SlCXE7 | MQWQTCVLLLCVTAILADDEWREVKTAQGPVRGRKHPTEDIYTFYNIPYATAPVGVDRFKAPLPPPVWLQPYDAVEKPVVCPQPVFPFPELQLNKLVKQENCLIANVFVPNTNKKNLSVVVYVHGGAFIMGWGDMFRATQLMNKKDIIVVTFNYRLGVHGFLCLGTEDAPGNAGMKDQVALLRWVQKNIGSFNGNPDDVTIIGFSAGSASVDLLMLSKSAEGLFHRVVPESGGNLAAFAIQRDPVEIAKTYARQLNFTNVDDIYALGEFYKTAPLELLTSDPFFDRTDSTFLFSPCVERNMGQETFLTESPLTILKNGNYKKLPVLYGFAEMEGLLRVDFFPFWKQKMSDKFSDFLPADLTFKYEEEREEVANKVKRFYFGDEPVSEDNILQYVNFFSDVLFMHPMLWATKLHVEAGNNQIYLYELSFVAEEVPEVPHTNIKGVTQCGQSLAVLDGKNLTHKSHSEESLATPEYQKMKKTMREIWYNFIKKGKPVPEGSTRPAWPFAGADRSPHMSLGQKIEPRGVLLEKRTVLWDDINQRYFREPVPPPTPPPKSHAEK |
| ApolPDE | MFLKLFIFVATVGLASTADSNPVVTVTQGALQGAWKTSAKGRDYASFQGVPYAIPPTGEYRFKEPQELTSWDGTWNATEPLSACLQYDPFSDSITGNEDCLYLNIHTPNISSDASLPVMVFIHGGAFMYGEGSVYDPIYFMDYDMVVITFNYRLGPLGFLSTADDVIPGNNGLKDQSFALHWVKNNIKMFGGNPDSITLTGCSAGGASVHYHYLSQLSRDTFHRGIAFSGSAFDPWAFAVKPVQNANTLASIVGCSSDTSTEILNCLMDASAEDIVNAQNEMFDWKVKLFSHFTPTIEAAEVEDAFLNKYPYQAAVDGDMLNVPLITAMNAQEGLYPGAADLIYLDEIESQWSVVARDLFKYNDTLSSTLWSDVAAKIKEEYFGDETVSEDTFSQLVEALTDRLFIMDIGRLAEIHALKSGQATYVYKYSYRATTSLSNLLANNEEDYGVCHCDDVLHIFNYPYIDARTDTDLQMIYNLCEMIYTYASTGTPVMAGSDIEWLPVTSGDSEINYLEIFSPNSTEMKSSSDFGRRSFWDNLGIIENKNYNATLIAEL |
| PjapPDE | MTFHARFLTILVFATGIFANNPFPTLEISTGVLQGTYKTSYNGRKFSAFDGVPYARPPVGELRFEAPKEPYNWTGTWIADTNPLCIQSFVGVKELGVSGEEDCLYLNIYVPREELSHKDNLDVILHIHGGAFMLGSGHFYAGPEYLMDEEVILVTINYRLGPFGFLSTEDEIQPGNNGLKDQVQALKWLRKNIKYFGGNPDSVTLTGMSAGGASVHYHYFSPLSKGLFHRGYSQSGTALNPWAFQEASLDKAKRLAVSVGCPIDTSLNLIHCLKSRSAYSITEAVKHFFGYGVLPFSPFGPVIEKKHDGAFITEHPYKMLVNHQVADIPWITSVTEREGIFPGAYFTDEPSVLEADFYTIAPHALDYNYTISEAYKLSITDKIKKQYFKESINVHDFITLLGDRHFKADAGISAKLQAKANKSPVYFYLISYRGERSFSELFTERTDDLGTSHADDILYYLHYYALTSGKLSNADENMKSIFLEFLVNFMRNGKPKISNIEWTPVNNTNTLHYLHINSPEDMYMATDEDLGAQRFWSKLSLRENSNYDEVKDEL |
| GmolCXE1 | MKWLALFSLVAATVLQQPAPTVRTRGGLLRGRVARDGRLLEYLGIPYGTVNEENRFKAPLPPPTWDGVFEAINTNTRCPQTVMGSVIIGNPDCLKLNVYTPARTRSKLLPVMVYIHGGCFFEGTGSAFLYGADYFVEHGVVFVGINYRLNVEGFLCLGTEDAPGNAGLKDQVAALRWIKDNIEAFGGNPQSVTLFGESAGAVSSSFLVLSRAAKGLFHRVILQSGSTLAPWALQHDPIRTASALVKHFGYDTKDPYEIYDILRSKTVNELIIAKAAENQNYISSDTLFVPCIEKELPGIEPIVTEYPTSIIESGNYTKVPMIIGFNDNEGIYFVAKDYGSSLKSIDPKENLQPDLEFPTELEKNNTVDIIKSHYFPPEKDELILNMVDLYSDLHFKFPSVIESDLYAKTTDKPIYFYLFKYGGYRNMAKIIVGYGATGGASHADELFYLFKPHSIPFPHQLERRMIKRMVTLWTNFAKYGDPTPTTSPLLPFRWQPGRPSNPSALVIDSQLSTARLWDYATVDLWNQTYSKYRRKVYGFH |
| GmolCXE14 | MTHGIIKLLLLVASAALCVTQDAKESRIVQTSQGPVRGYKSDGIFYFYGIPYATAPTGTRRFTAPLPGPVWLNTLNAVNDKIACPQGALQMVDVAGYDMREDCLVANIYIPDTDETNLPVVVYVHGGAYQLGTGALGSPRALVQTKKVIAVTFNYRLGAHGFLCLGTENAPGNAGMKDQVALLKWVQKNIANFGGNANDVTIAGYSAGSSAVDLLMLSSMTKGLYNKVIPESGASLAAWSVQVDPIYNAKYYAKQLNFTKNLDDIESLEDFYSTLPYETLMSDTFLDRKESTFWFSPCVESYNGVEKFLDDTPLNILTKGEYRKVPVLYGFANMEGLFRVPLFEQWREAMNKRFSDFLPIDLQFQNNEEKERIAQEIKEFYFGKESISVETIQGYIDYFSDVVFAVAHLRSVKLQVQAGSDSIYLYEYSFFRPQPENAPVPEFVRDIKGAGHCAQSFTILDIGFIGNSSPESEEYKEFKKVMVKLWVNFITTGKPVPAGSDLPSWPPVGANGSPYMDLGEEIKLKGALLEKRVQFWEDIYARFYRSPIAPNTREVRSEL |
| DcCCE1 | MNYINIIIKHIFLLVIIFNAKSSLVFASLTSSRIVRTKYGELSGLVSTPSDRLDAVEIFRGVPYALPPVGHLRFMPPVSGALWSGVKVADRFSPVCPQNLPSPVRMRANNTNAHPRGRLEYLHRLIPYLTNQSEDCLYLNIYAPAQGGSRETGPRKYPVMVFIHGESYEWNSGNAYDGSVLASYGGVVVVTVNYRLGILGFLNTNTDVHMRLPSNYGLMDQIAALHWIQENIGYFNGDPSNVTLVGHGTGAACVNFLMISPAVPDGLLFHRAILMSGSALSPWALVRDPARYARQVAKHAACPRDLSHTELLKCLREKPLELLLSTPVVAPEFTTAFGPSIDGVIIDTGLSETRPANNKDSHSYDYHTQNSPGRPPPPPSASENINEAIAAGGYGNAVLSNAIAKKAMVSKLTRYDLLLGVVKAEAFFTFTGDDIQYGIEPDRRSKILRTFVKNTYRYHLSEILATIINEYTDWERPVQHPVNIRDETLEALSDAMVVAPSVNTADLHSSARRNSYLYVFDYQTKNGDYQQRQGCIHGEELPYLFGAPLVGGVNHFPRNFTKSEILLSEATMIYFSNFARTGNPNDAQDGGDMVHNTVRAERTKFKNIEWTAYESVHKKYLSLDMKPKLKNHYRAHRLSFWLNLVPDLHKPGSDDVPRSHHMLGDDDEINEIPHSTSSPGKHQAPPSTYQNYGPSASHNLVNLTSNDIIHQQASAAVVKVAPSSGRDLQEDLPDGSAAASGQEDGFAAYSTALSVTVAIGCSLLILNVLIFAGVYYQRDKSRQGGSGHQDSAGSIHSNTSSNGSQNGAGMIKQRRQENGGPMSNISADYLYVSGAPELLQIVETSRSSPRSGGGTGPSILKKPPLENSSILVLQPPHRYPPIEFQDVKISCSATLPRAPPPPRLPESQPLLSGGGTLGRYKPQHKTIDELKV |
| DcCCE2 | MRIRLINQYQLLIYIILYLDYIAPRIVRTKYGDVSGVIINADNRYLDAVEVFRGIPYASAPVGNLRFMPPTTPETWKDVKPAHKFAPVCPQDFAAGATGGAVGAAAETPGAEKMRQPRLEMLRGYLRNQSEDCLYLNIYSPIRKDESKTSDKTPVLVYIHGESFSWNSGSVYDGTVLSSFASMVVVTVNYRLGILGFLNPSKDYRARIPANFGLLDQIAALHWIKENIGEFGGDPSNISLMGHGTGAACINFLMTSPAVPDGLFQRAILMSGSALSSWALVRDPAQYADQVAAQLNCSSLEAAEPLLQCLRNLPLEALMTVRLEVPDYTTGFGPSIDGVVIDPEIQDDAGRVEYIPGKLSASASFLASFLSDGYYTSLLGHSAAIAALIAKLGRFDILAGVVKSEAFFAFSDEDIQYGIENTRRYEVLKKYVSNTFRFHQSEVLSTLINEYTDWERPVQHPINIRDETLDALSDAMYTAPLWTLADLQSKGRNSCYLYVFDYQTKYGVYPQRQGCVQGEELPYMFGAPLVGGMGYFPKNYTKPEIQLSEMLMTYLSNFVRTG |
| DcCCE3 | MCVFVDNLTLLTLLILFHLTVSNPPGGIGGPSFTRIITTRYGKLQGVIKKINENGISKHLKPIEMYLGVPYATSPTGANRFSPTRTPEPWDGIKFVNRVSPVCPQRLPDIADETEALGRMPRGRYEHLKRLLPHLRNQSEDCLYLNIYTPLQEVGQPLQKYPVLIYIHGESFEWNSGNPYDGRILASYADFVVITLNYRLGVLGFLNANAAPHMKARVANYGLMDQIAALHWVQQNIALFGGDPGNVTMMGHGTGAACINFLMISPTVMPGLFHKAVLLSGSALSSWALVEDPVHFAVQLASQLNCTVPDIHADHESLVDCLRDVPLSRLMAADTGAPSFLSAFGPSVDGVVIKSNFQQELLLNILPELQGYSAGSIALGTPINIKKNSEILLGGTNKYDLLFGVVTSEALSGFSNGDIEAGFEGERRDKIIRTYIRNTYVYHLSEIFFTVINEYTDWERTVLHPINTRDATIAAISDAQYVAPLVQIGDLLSRPQLLQSGGGAGNGTSGAPNPPSSPGHTKSYFYVFDYQTKDGDYPQRMGTAHGEELPYIFGAPLIDGFSHFPRNFTKSEVALSEAFILYLSNFARTGNPNEFQHRAEMTLAGSKEKNRFRSITWDEYDPVHQKYLEISMKPRMKNHYRAHQLSVWLRLIPELHRAGMEGEGGASARHNVFRAVEGAELYEGVVRSDPLTSGRRHVMTSQNGTVAEIIQQPIITSSIPSHQLTSCLNITQTSQHQQNLHPNYHELSNLDLSNFASYNTALTITITIGCSLLVLNILIFAGVYYQKDRNNSNKSSGKKDRSSRKNSMSNSNSSCYLDAATSKNLLNMDTSSIIVDIERDMMLTEDKQQHCDLMLSHPQTSNAASMKTLSHLATLPRNKHIMSDTQCVTSLNTQYNAMTSQPCCNNVATLPRNITFNPVMTYDCKMAQSTGADCNFRSSNVHESFRQSPNVLECSRSSYQGNPCDGGGAKNLIKLNSSTSSSTGSGGSNPPGGNLSEMRV |
| DcCCE4 | MRPCLIAALLLLALVSAAPRYSSRIVEISSGAIRGIILEPNSRHLEPVEAFLGVPYAAPPVGQLRYAPANPPLNWTGTRLADALGPVCPQKFPDLSNKTAALQHMPVGRYQQLLRMEPYLGNFSEDCLHLNIYVPGSGSRGVDAPYAVIVFIHGESYEWNAGNHYDGSVLASSGHIIFVSINYRLGILGFLKTQTGHTQSGNWAVSDVIAALEWIKINIASFGGDPTRITLFGHDTGAALVNIVLLTPSVKGLFHRVTLLSGSILSPWSFVHDPDSIRSNVADQLGCTLSDNLAPCLRTHTLASLLQVNLTPPRFLAGFGPHLFTDPNVALEKAGDNFVTTPLMAGVVTTESYLNFNANDIQYGFEEDQRNRVLRTYVRNAYVYHLNEIFSTIRNEYTDWDKPILHPINIRDSIMEALSDGHTVAPVIRVVYLHARRGAKTYMFHFAYQSKDSEYPQRLGSVRGEDLPYILGLPLVQGLPFFPQNYSKQDIAISESMVTFFTNYAKTGDPNKHDHRGVIKDRSKYWDPYETGTQEYLSIGVKSKMKSHYRGHKMAVWLNLIPQLHQPGDEDVSMRHHHFHERANHYYAGVVKPETFTKLPPLLTTTTKLNIECEPNSTLPNENTLLDKSHDHSNNGVNEDEDSEESLLEKIARKHYSYTTALGVTVGIGCLLLILNIVIFTAIYYQRRRKKKSRERDGKSDEINSDINHSDNFDNFSLTQLSIAVGDKSPYSNSIQLNNTNAILTKSPSIPEPPPPPKNMPPLVVVPPPKLPNRLNSCLSNSSTNVNSVKKRVQIQEISV |
| DcCCE5 | MRYLLLCLYFVWLTSAHKYSTRVARTKYGPLRGILIQNPPVEAYLGVPYATPPLGSLRYMPPVTPSTWRAPRFADTYSAVCPQRLPDIGNRTEALLQLPRGRLVFLEKLLPLLSNQSEDCLYLNLYVPRPENSMDDIGSPMPALILIHGESYEWNSGNPYDGSILASYGHLIVVTVNFRLGVLGFLKTGARGSAQGNFGLMDIVAALHWLRENLHAFGGDPDRITLMGHGTGASLANILAVSPVAKELLHRVILLSGSGLSPWAIQRDPLAVKRKVAEQTGCHGDLLEDDIAPCLRGKSLQELLDVRLEPLRFLPGFAPFVDGAVIASSVMSSVSLSDVGILSGNKEGPGYELADFPDRDLLFCLTSTESYLDLSAQDLEFGFNETRRDRILRTFVRNAYYFHLNEIFSTLKNEYTDWERPVLSALNYRDATLEVLSDGHTVAPLIRVGHLHALRGGRSYFLHFRHQTSERDYYPQRTGSVRGEDVPYVLGLPLVDGGPFFPHNYSDQDAAISKQLIHYIANFARKGDPNGPTPPASLDPNHQVPFWDTYDSINQLYLELGSKTEIRNHYRGHKMSLWLNLIPQLHRPGVEDLSMRHHNFLEDGVQYYDGSVRPQTLYRPNLLMPEPTTTSRPKATSTTQPTSATAQPDITTTECVNTSLGPAVDMVRPTKDINTNILKKYANNQYQHYATAFIVTITVGLLLLLLNILIMARICRQRENRSREKKKKPKKRKEDIHAGGPDANSISSSSIEEFKHALSESMNPSVSTMVDLPLQDFKCSPPSGTRRPSSGTLGGPDICPPGYESTDVTSPSIPEPPPPPKNQPPPGCPGNPSILRQQNCPQTPPNLMKKRVQIQEISV |
| DcCCE6 | MASCGALFTGLLLLLLLQVTMSSKEYTNIIELKQGRLRGVVRSPVHNGNLNNIHMYLGIPYAAPPVGQLRFMAPQSPQPWPGLMIADSFSPVCPQKLPNLDDDDFSKKMSKGRLQYYQALMPYLKNQSEDCLYLNIYTPLQDSSRTYRRHSVLVIIHGESYSFGSGNIYDGFVLASYANMVVVTFNFRLGILGFLRPGVGSSTVTNFGIMDQVAALQWIKDNIEHFGGDPTSVTLMGHGTGAASINFLMLSPLLSPSYDLFKRAILLSGSALSLWSLVTHPLQYTLQVAQHFNCPETDPEMSICLRNKRLSDITAVDIESPRFKTAFGPIIDGNVIPNDPEQCMTVYRDIFSRYELMFGLTQSESYHQLDGISLLFGMKFEQTETLLRDFVYSKYEAHPDVALAKILQEYNNWNKSLAGMTPKSEAEQNRDNVLSILNDALYNAPVIQMGEYHAKANRETYFYVFGHKTDNGLFPYTDQSVSGEEVPYFLGVPLDGDLSYYKSKYTTREKLHSEVILTWVSNFARSGNPNMGMRDSFQFLSYSDWAPYLHLHWPPFDPNNQTYLQMNIPPRVSKHYKADKVRYWNVNLPHTLSQCLTPSGGIDKYASRYPSILREFPPNPEPDRDYAEPKYGRRPEPEPKPRDFDPRPWIEQPLPKPNEAREDFNPYDNFGTRPQFGRTEKSDPSDDENVKSSSISGILITLGVIFLLLNLFAFVFIFYQKNRLNMREHLFNNARMRHFQCTSRNTDGDDEYEFTDDEVESQIYMKKTSSAVTTPPDVKSILKHNNSDYEAVSTNKQNYERKNSSSTVDANMKVRQWIVEKCNGETQFEMDGTSDDGGKAPKAGTARDFNQKENIYVDRKDRADVRKEGNYDVIKTRKTSNDSSSTKKQGVKKVSVAVDATPATRTASVLRQIPIEITKSSKSLNEISKDESEIPLVLRKNKSFDYPDDEFIDEKSRTLPSNFNKKRSMSTTSIHELKQLIDSNKTFQEVVTAKTKTLKKPKTVHKNEANILDDGKLSGKLNADINVTSRDEKEILPLTHQQTMNGIMRRNFPKVL |
| DcCCE7 | MGPPVLLLVWLSVATSATVLRHTRHQMSPPSPDLSEDFSSTDAIFHHKNLLDLRMDADATSERIIDDEISITESDEEDPDDLEDHSMRMQADQPPLPGDPGSDPLVVKTTKGRIRGITLTAPTGKQVDAWLGIPYAQKPIGNLRFRHPQPVDRWDKDNPNLILNTTRQPNSCVQIVDTLFVNFSGAEMWNPNTHMSEDCLYINVVAPRPRPKNAAVMIWVFGGGFYSGTATLDVYDPKILVSEMNVIVVSMQYRVASLGFLYFRTPDAPGNTGLFDQLMALQWVHDNIEYFGGNRNNVTLFGESAGAVSVSFHLLSPLSRNLFSQAIMQSGAPTAPWAIIDQEESILRGLRLAEALKCPHDRNRIQDAIDCLKKVNATDMVNSEWGTLGICEFPFVPIVDGVFLTDIPVKSLRNKEFKKTNILMGSNTEEGNYFILYFLTELFKKEENVYISREEFLNSVHQLNPYFHPIVRNAIVFEYTNWLNPDDPISNRDSLDKMVGDYHFTCNVNEFAHRYAETGNSVFMYYFKHRSYNSPWPSWTGVLHADEISYAFGEPLNPRKSFAASEVELSRRIMRYWTNFAKTGNPSLGDDGQWTPVYWPVHTAYGREFLTLDVNNTETGRGPRLKPCAFWKKYLPKLIASTANCSTDLSKRPIELSTATSSARGLLSAHHSKQSFYISSILNLVYTLPLYLMLKLLY |
| DcCCE8 | MNPALILSLLFLYPVHIQCRPRAHQHHSHDHHSHAGPHGENHSYERHSYEHRDPLLIETKSGPVRGYSKEVLGREVHIFTGIPFAKPPLGNLRFKKPVPIGRWPGFIDATKLPNSCYQEKYEYFPGFEGEEMWNPNTNLSEDCLYLNIWAPQRLRIRHKTGEHDTKRQLAPVLVWIYGGGYMSGTATLDIYNADIVAGTSDVIVVSIQYRVGAFGFLYLKGLLPDHFEDAPGNMGLWDQIMAFEWLRDNIEFFGGNPDLITLFGESAGAASVSIHLLSPLTRHLFKRGIMQSGTLNAPWSYMTAERAVEIARKLLDDVGCNGTTNLEDNPERVMSCLGAVDSKTISSMQWNSYTAILGFTSAPTIDGELVPKHPLAMIEDMSKDDNLTEIIIGSNQDEGTYFLLYDFIDYFEKDGPSTLPREKFLILVNQIFKVKPESEQAAAIIHEYTDWENVMDEHLNQKLISDAVGDYFFICPTNHFAQTYASRGGKVYYYFFTQRTSTNQWGEWMGVMHGDEIEYVFGHPLNRSIEYNARERDLSLRMMQAYARFALVGKPVPDDVEWPLYTKDHPYYYIFNAEKSGTGKGPRARTCSFWNDFYPKLIPPPLDPITTNCDLDRSETPDDYAVLIPPLQSVFNSNMTSSAPSGHLLHTLVLILTTCVAVIVRF |
| DcCCE9 | MSFKYLSLILLLPKCYFTQKPFDPRDIQLPVPGDPNYKTYVHNSRRYGQAIPIEKFPKFSDTPRNVDSNFAYPGQNFDPAFNPNYNPALNPNYNPSSGNYYDPLNPFNLPDRSLSPGQPGSSFGLPGVLGGWRTDLQGKLRPDSFNLNSNKDLFVKTPYGQIQGFKVNLYDNPHPEYSVRPEYNPVEKVQGNVSVFLGIPYAMPPVNEGRFKPPRPHKGWQLLQAVDFGPACPQPVEMVGPSKGVRDMDEDCLYLNVYSPMTGAGVSRPYPVMVYIHGGDFSHGASNLFPGHMMAGFYEVVVVTINYRLGALGFLSTGDHNSPGNYGILDQAMALQWIHGNIEHFNGDPNSITLFGPGAGAASAGLLMVNPRTRNLVRRVIAQSGSATADWALIQDRWRVQNTSRLFAQHLGCSFESSWKIVDCLKRGRSSLELGNANFRPQVGMFAWGPVLDLNFTVPADHWHDGWYQKDWYFTNYTTEEYIRMGSFSRDLAYMTGVTTQEAAYIVANNASLKPYYIIDSVAFDQKVKELVLQYNYTLNTQGVYQAIKYIYTYWPDPTNVTFIREQYINMLSDFLYRAPVDNIVKLLVEKNVPVFMYVMNTTVEAFRLPEWCKYRHNIEHYFLTGAPFMDIEFFPSKDLLRRNMWTDNDRNMSHFFMKAYTNFAKFGDPTFTQILGLHFEKASMGTLKYLNLNTTFNSSIQMNYRQTESAFWTWYMPTLVGVVTPTYPPFTEYWWEPRTPIQVAFWSVSTLCMVLLAAVVIFCILWQTAKRRVNHYYSGDIFVRDDTELTMPSVNGVGSSGIDNRDQRSVSNIYEYRDTPPIGSQKKSEVKGKMSASNPSLRTGSGSASSLKDSTSVVSSNPIGSLSRSPGGLKKGRSRSNTLDTDSVPQTTV |
| DcCCE10 | MNAHHLLHLFLYLTVVSIGFCKVTNITDFVPEVNIDKLGKVRGRVTMSHWTKRLIYSFQGIPYAIPPVGKMRFQSPRPAQPWTTPLNATSLKRRCPQIFDASSNIEVLTSTEALEDCLYLNVYTPMISSNVNQTEASQKLFPVIFYIHGGSFRVGSSHSMTPHYLLEKDVVLVTIQYRLGILGFLSLETNEIPGNMGFLDMLLALEWVNDHIRSFNGDKNCVTLMGQSAGGAAVTFFLTSPLVRDDLFHRAIIQSGSGLCDWSFENETGMGRQFIEDLGCRLDNVDDSFVAECLRRKSLSDLFKTYYSMEYGGKFCQAVKQTHGKKRFLTETPEESFAKGAVKNVPVLLGVTKHEGSSFLEVVFRVLWDDPIVENFTDFKVQDLAHQFMEYTRLDVSAIVSHLVETQYFSQNYATMKDVIPAMSDLAGLAVLKSCVFHTARLLSSMHNSTFLYSFHFKGRFTKYFEYRDKNDSRFPHGVAHSDDLIYLFWDPTMPLNDREQKQARTMVDLWTNFATYGYPFSEDVPDWQPVANDSLGPFLRIDERSELRKSFLEEFTIATREGVEAYYSENGGSCERTSWVHVILCTLLHLLLVKYKT |
| DcCCE11 | MTTLKWRLPSALGGFLVLALLYTPTASLSRQKRIVGGHPADPPPDANLNDVADTPDAWGVIVDKPVVFVDDDVQSARIFGASEGNKYYAFRGIPYAEPPLNEFRFQRPKRRYLDGDIHAVKNGNPCLQPSPNDPKKVVGDEDCLTLNVYTPKIPTQNDPNPELLPVIFWIHGGGYRRGSGLQYDPNDLVMKNTVVVTVQYRLGSLGFLSSKQKDLPGNVGLLDIASALHWTRHYIQNFGGDPNKITTAGQGSGASAAMLLSLSKLTSSWVQGIVAMSGSALSSFAVDYRPEESYKNVTRKSTVCSDMTGVELVKCLQELSPEEIVLSDTDIESSNIQNGGFVSGLAELLTPGPVVEGEDDEWFLPNLLENSAMDLITSTNKTDKIPMLTGVTKQETGTGVKGGFRMDIVGLLSNTTNFLTDGLPNKLLHANKGLLNNIQLGNALKKLFSNVDYLTGISKSIEAVNSELDKVVQLTTDSLFNLPMFLTSKVWSLTSKTFVYSFEHLPQRSTASHFLTGLPLIRGSDELKKPEISHGEDLIYLFDAKSLDGNSQVHKISNSDDMQIRNMFTSLVAEFARSGTPQLPNSNVKWSPFSAQNSEFLVLSKTPRMDKEFRHCQMALWEGLAEVLQSQKCSLLNLQKLLGTEKGLLGGLTGVNSAGKRVDLLGGLTGGENNLVGGLTGGLTGGNGGLLNLGGKPPQQQGGGNGGLLNLGGQQPPKQQGGNMQRPPVQNQGLLGTGLLGGGLKSAQDQPEGTNNQVGSGNFGNPGPTKPKLLGIL |
| DcCCE12 | MKLLVGLLCVFVIASRGLCLEVPLVHTENGAVSGTYKLSTGGNRFLSFEGIPYARPPVGKYRFREPQLLKPWIGVWNASSPGSDCLQYDHLTYLADNPFLGDEDCLYLNVYSPQLPTANNQLLLDVIVYIHGGAFTYGAGRWYEPHYLMDRNVVFVTINYRLGPFGFLSTGDEIVPGNNGLKDQVAALKWIQRNIAKFGGNPANVMLTGMSAGGASVHYHYLSPLSQGLFHKGHSMSGSVLLPWTQAEELRKKTEDLATLLGCPVYTSKEIIDCLRHRPAKKIVELVKTYFMPWHFNPYTPFGPTVEVAGVAPFLDQDPHEAMEQGNVKDLPWIASVTSEEGLYPVSNFVANSSLLEELENNWEFIAPYLLDYNLTVDSRVKNQVSNKIKQHYLQGKPISPETTLEFTHMVGDRLFVEGVEAAAKLQAQAVSSPVYFYRFSYRGKHSLSEIMSFGSLENFGVSHGDDTGYVIKVNFMNPLETEEDKIMNSRMLDLWTSFAKTGVPSFGPDVNWSPLDASGELKYLQIDGPNSLNMKTASSVGEADFWHSLPINEPRHNVGANQKTEL |
| DcCCE13 | MNCRTSLILTLVGFITCLSVTQGAVVKVKQGLVSGTTLTLRNGKVVNKFIHIPYAQPPVGKRRFKDPEPIKSWAGVWNATNEEGDILKCTQFMHVPGGPNSVGGQEDCLYLSIYTPKLPQSGDQSKLLDVIVYIHGGAFMFGQGFRYKPFPLIEQQDVVYVEFNYRLGPLGFLSTGDDVVPGNMGLKDQTQALRWIQENIAQFGGNPKSVTITGMSAGGASVHYQMLSPQAKGLFHRAISMSGTSLCPWTLAENLPEKTKIIANQLGCPVECNEKMVECLRSRPAALIADAVRLTQPFYYNPFSPWGPTVDSFAKNPILPDFPAELIKQGKIADVPWLNSVTTDEGLYPAAEFLASEEALKTIDADWTSLAPHILDFNFTVPDNLKAKIAEKIRQKYLGDKPINLENKKAFVQIISDRMFIADAERTSRLQSKVCKSPVYFYYFNFRGRYSLSNHYANRLDDYGVSHADDTQYILTVLGDAIMRDTTEDEQKTMDLLNTLWASFAKTGKPHISSWKPVTPNSFNYLQIDSGENYRLVENAAEIGSRSFWDSLPFDENVWKPEATSHTEL |
| DcCCE14 | MMSFSQLSPVWILLAALHLAHGQVVQINQGRLRGTAERSRNGVKYFSFLGIPYAQAPEGELRLKDPQPHPGWSDTKNATEHGNECPQRNYFNHQLIGSDNCLFLNVYTPKIDPNAKLPVMVYIHGGAFKGGNTRFLKEKFIMDKNIVYVAIQYRIGILGFMSFLDDVIPGNFGLKDQIFALKWVQDNIAHFGGDPSRVTIFGGSAGAAAVDYLVISPLAKGLFHNAIIQGGTATSPWAYIPQTVAKQRAEAVATLLGCPSKPTTEALACMRDIPSDNFIIVTDKFLEWDLSPLGPFSPITDSFMGAGAVVPDHPLALPPNPVNIILGYNSYEGNMIASMVCFNEFRLARDMEVDLPRRLALLTNLQDQVKYSEKAKVADRLFEFYLNSQNISKDNVYKFADLGTDILFGHPSFKAALNYYKKVPLYFYLYDITPRITLLTMFGNCTHLRGPSHGEEIVYFFNDIIPNFELTPEEHKLSHRLLDLWTSFASTGVPSDTWTPVASDRIEYLHMTNDGFKMARGLYEDRMRFVDTLPLLNNQYNLRHEKTEL |
| DcCCE15 | MVLAAFAVASFCAFCGHVLATETLIIPQGRIRGTVRQSRDSNPYYAFLGIPYALPPVEDLRFKDPVPHPGWSGERDATTDSPVCPQAMPYFPEKGDAIFGQEDCLFLNVYSPNPSSDAKLPVMVFVHGGGFLMGQATSNMYGPEYFMDHNVVLVTIQYRLGVLGFLSFGNAEVPGNLGMKDQVLALQWIQENIEEFGGNPDSVTIFGESAGAASVSYHLVSPLSKGLFHRAILQSGTASCSWASTPAWLARDRAHAFATLVGCPTQPIETVLDCLRQLPTETFVTTLNKFHIWFKNPMITFAPVIESPLSQNNFLPDHPLRLPHADVPIIIGVNNKEGGIVGAVYCTDDFKLTHQLNADFDRLLPLIVSCNWLPKDKRAEFVQKVKSFYLNNQPVGEDNLEGLIDLVSDLGFFQGAVDTALHHSGYAPAFFYLYDYELPVSLNSMFGNCSQPLGVGHGDELFQLFRMSVISNYTHTPEEVTASNMLLRLWTSFARNGIPDHTAWGPVPSRAVTSREIEYLYVSRSSVSIHQGFVPERIEFASALISEYMNYGSNDRRDESRSPKTEL |
| DcCCE16 | MLFLILVILFNLYFASGQVVHIKQGTLVGFRNVSRQGKEYVGFLGIPYAKPPVNELRFQDPQPHPGWSGERDATKMNNVCPQVTILMPNATFGHEDCLYLNVYTPEADVGAKLPVMFLIHGGGFRMGAANFYNEKFFMDQNVVLVSINYRLGVLGFLSFADEVIPGNFGMKDQVFALQWVRENIAQFGGDPNSVTIFGCSAGGASVDYHIISPLSKGLFQNAIVQSGTASCPWAMTPSTIARQRAEAVATLVNCPAEPTSEALACMRDKTAEAITLTDAKFQEWVSSPLVNFPPVVDSYLGQDAFLPDHPLKLSPNNVNAIIGLTMREGNLVAIPMCKNEFQLAKELEADLANRLPILMCIQDHVDYKDRKKISEDLMNFYLKGEKIDEHTVGENFQELGTDLLFTHGSYKSALHYYKRIPVYFYLFDIAIPEPNAFTHYLGNCSSAKGPGHGEENYFMFNDLFSAFLGSTPPLTKEDDGLSKEILQLWTTFAAKGVPDEIWTPVVSDKIEYLYMTREGFSMRSGLFESRLKFVNSLPLDVNKYKLDRERNEL |
| DcCCE17 | MKILGLLILCCLGYIWCETVTIKQGKVRGTQFESRGGRKFHAFLGIPYAKAPEGELRFKNPEPHPGWEGEFDATSEGAMCVQEMTLVPRFHSIPMGEENCLFLNVHTPEINPTKKLPVLVWIYGGAFQMGDSGNMTYGPYYLMDRDVVYVNLNYRLGVLGFLSFETPSLSGNFGLKDQQLALQWVKDNIESFGGDSNSITIFGESAGAASVHYHLLSPKSRGLFHKAIMQSGSAFCPWALIPPGIAQDRAHAFATLLGCHGDVNDIVACLRQIPSDTILATQLKFMEWGVDPLISFGPVVEPPHDEAFISQHPYLLQTDPSIPLIIGYNRDEGAIRTSKLCADDMLEMNQLNARWDRYLPLTILLKHNVEQKDMQRVMESLREYYLAGRKVDKHNIRGMTDMFSDVLINHCVMQAAEHYSGPVYFYMYNYQAAISWASVFGNCSIPLGVAHADELINLFNLDSLFPDHQLEGSDLEASKTMVKLWTDFADKGVPISPNPYQEASFIWPPFDVRTGKAYLHIHQNGLAIEEQPFEERHKFVSSLPFKFHKLVKSKSNAKTEL |
| DcCCE18 | MKLKLVTHCLLTAWKSTSPGLPRKMSTKVISTSYGKLKGSLKTSKYDETKYFSFQGIPYAKPPVGNLRFKAPEDLEPWEGIRDALKEGNCGPQYDMGTKIYIGDEDCLYLNVYVPNVAQTTLLPVMVWIHGGGFAYGHGNADAYGPEFLMNKNVILVTVNYRLGILGFLNLETKDAAGNMGLKDQNAALKWVKKEIENFGGDSKNVTLFGESAGGASVHYHMLSPLSKGLFHKAVLMSGSAMCNWGFTKNHLQRAFDLGKLLGCEAKTKEELLTFLMKASPKEFAGNQDKVINDVTKPLCLLWXTEEPLQSLRTGNVHDLPTIVGTVSHEGFLIYNSVAKWPWFVEKLNKELEAVSPYPIAPSKELSKSIRNVYFQGDETICFKKHNEQFVQLFSHTHFLVPMFMSLKYELNSPSRKAPIYCYRFAYDGNLGWFKKLMASSRKIDIPAGVSHVDELGYLLSNDLVDHKKLATEDDRKIVDKFTTLWSNFAKTGNPNPADHQVWSPIESYEQRNYLDIASPSSIVMKKNLDNDKIDFWVNKLNPNPADHQVWSPIESYEQRNYLDIASPSSIVMKKNLDNDKIDFWVNKL |
| DcCCE19 | MTEIIINTELGQIKGVQRSNALTNAALYSFQGIPYAAPPVGDLRFRPPQAHPGWEGTLDATKEGGICVQNDVMLGMFESGSDDCLYLNVYSPCITAGANKAVMVFVHGGGFTFGHPAEVFYGPDWLVAKDVVLVAIHYRVNIFGFLNLGLEDCPGNVGLRDIMASLQWVQANISDFGGNPNNVTLFGESAGAASIHYLLMVPSTRGLFHRVIMQSGSALDSWAFHTAEDNRDNGVAVAKLLGCQSEDSRSVLDFLKSQPALDLLKPQEQIVAAAAKSGKSLVFPFVPSVECTGLPENRLVWDTPAVMLKQKLPSIPFLCGLNSQEGIIMLKQSPGALTKVFDSLDRNFERTVPNNFHADRAKAKLIAAEIRQFYFEDRPIDMGAINRYLDLYSDLLFALGHYETLFSYSQSNPGQGYAYLFSYEGDLNVFKNAVQMMYDLQIPGASHVDELGYLFCVTMMGGVLKPGSTEEKVSENIRTLWTNFAKNGVPNTETFTSWTPCSHNDISYLDIGSTLSTEQGFVFSNRFAFWKNLYASYQANFLKGNL |
| DcCCE20 | MGAYGIPLLIYWPTLIMHRYDRRAVSILSRLIFIFSSVQGVLVNAQDPTVTLRQGTLKGIKIYTADARMSVIAYLGIPYATPPIGNLRFAPPQRHQGWERVFFAGSFGPACPQPHETLVPNVAAVSQQDENCLFLNIWAPEISLNYRNTPVVVFFEGEGFVSGTPSRFPAQDLAAEGLVVVSVNYRMNVFGFFSLEDAEARGNLGLLDQYLALLWVRENIPYFGGDGKSITLMGHSAGAASAVYHMVSPKTAGLFHKVIIMSGSATSPWMKTRSVSQASRSIARSLGCLTVNETRPILKCMRDKSTSELMRAFENQYKDGNWTELVLPVVDTFLPDIEQYLPRDPITALEEGMYHKVPVLTGITSHEGAISIARWSDLISQGFANLRQLFVNSIIPSVMNMYGFASHMSSVPEIRTLLEWQYVDNVPSGDAHALLSKILDFFTDSQYRAPYELQLKLLTKHDSENIGATYAYQFEQTSPDLYSKLVNISGMFQGCGHGQELIYLFGPALMQQVTGRRFTPNEDRLSVIVRRLWAEFMRVGHPAANSYGYGIPWKRYNHDLDNYVVIKSDPNLPPQQPILRQPSTSVINDVAARYRISLWNELLPKMHNLSVILEQINMLNQIRTEMQLAEQPYRSVMFTLIGFVFLLLVLLVICVVLLKRKSEDRERDLF |
| AcypiCCE6 | MRCIYIVWILFLKLVCCELEVRIADGNLKGKVLQSRDGRTYYSYTGIPYAKPPIGELRFKAPQPVEPWNGTLDASRESNKICIQINVADNDEDCLYLNVFTPKVDKKFLPVMFWIHGGMFSLGHGGPGNHGPEYFMDKDVLLVSINYRLGVLGFLSTEDDVIPGNYGMKDQVMALRWVQKNIIHFNGDPSQVTIFGGSAGGASTGFHMLSPMSKGLFHKVILQSGSPVCKWAVSPIGLPRKRAHAVALIAGCNFDTSEDILQCLRKLSADYIIDLQNRLYAWLSHPCIQFNPVVENCNSGQEAFLCHHPIYDFKQESFVPAIFGLNSAEGGLFVASLYNSTSLMYTELQTYFNRILSIMLFYNQYTRPEDLDEIGERVLKEYFPSGRLDDNTHSNAVNMIGDGCFAHCIVDMAKKLSSPVYSYYYDYQNEFSYNKLFGSCQKSLGVTHVDELNSLFKNNNLNSNDLNADNLKVSRLMVNIWYRFVTSETPTIDGTDTGTDWPKFTYDEQFSVLHIDSAQPKIIQNPFDEKYKFWNQLPYSFRLNQTMSGKMEDIKTEL |
| AcypiCCE7 | MYFSRTWTSNQVREKLVYAVIILFLSSAFCQVEVKITKGILKGRVLKSRNERPYYSFTGIPYAKPPVGELRFEAPEPADPWDGTLDVTKHSNACVQKYETNSSEDCLYLNVYTPSTDGNFPVMFWIHGGAFYLGHSSPDMFGPDYFMDSNVVLVSANFRLGVLGFLSTEDDVIPGNYGMKDQVAALRWVQENIVKFGGDLEKVTIFGGSSGGASTGYHMLSPMSKGLFHKAILQSGTPLCRWSTSLPGVARNRSQIISRIAGCVGNSSSKNILKCLKALPASFFSDVYDKLREWHSYPTVLFSPVVEKCDTGKEAFLCRHQLNEFKQESFVPAIIGLNSAEGGMIVSSLYNDTSLRYPEFYTDFNRLISIILSHQHFTLDLSEIGEKVLGKYFPSGKIGDHSHLKTVEMITDGRYLHGILDMALKLVSPVYFYLYDYQNEFSFNTLYGKCNKKLGTTHGDELTSLFKSDTINPKKMNSKDTGVSKLMVDVWTKFATEESPTIDGTINGPLWPTFNSQRQSILHIHSDHPVIIQNPFEGKYTFWNNLPLLSNFKNVISKNIFPPDHMKNEL |
| AcypiCCE8 | MVSGTLKSTYNFRTRIKMIIKWTFIAVVLYLDLVQCIPQELKITKGVIKGQILKSRNGRSYYSYTGIPYAKPPIGELRFKAPVPVGPWDGILDATKESNICIQQGSTDGQEDCLYLNVYSPKTNEKSLPVMFWIHGGGFTWGHSRSGLYGPDYLMDKDVILVTMNYRLGIFGFLSAEDDVIPGNYGVKDQVAALRWVQENIMHFNGDPNRVTISGGSSGGASTGYHMLSPMSKGLFHKAILQSGAPVCKWAVLPPGIPRKRAHAVATISGCNFDTSEDILKCLRQIPAQYLIDLHTKLFTWIVHPFILFNPVVENCNTGQEAFLCHHPIYDFHQESFVPAIVGLNSAEGGLFVAAMYNNTALLHPELRTDFNRLISIMMNYNYYTTPENIDEIGEKVLKKYFPSGGIDDHTHINAVKMFTDSCFTECVIDMAYKLSSPVYSYFYNYQNEFSYNKAFGSCERPLGVTHGDELNSIFKMNSLNPNDLNEKDLEVSKLVINIWYKFVASDNPTIDGLENGLPWPEFTKPNQSVILFNSSHPSLIQNPCMDAYAFWKKLI |
| AcypiCCE9 | MWVASIISILFLSAVICDPVVRITKGAIQGVTSKSRDGRDFYSFTAIPYAKPPVDELRFEPPVTVDSWGGILDATKESNMCTQNNHFFPAIRHLILGQEDCLYLNVYTPNLNGKLPVMFWIHGGGFLAGHSGSNVFGPEYFMDNDVVLVSINYRLGLFGFMSTEDDVIPGNNGLKDQVMALRWVQENIANFGGDPGQVTLFGESAGGASAGYHLLSPLSKGLFHKAILQSGTPLCRWAVSPPGLVRKRTEAVATIAGCNSDTSEEILKCLKKLPANYMVELHNKFFEWINHPCIIFPPVVESCDSNQESFLCNHPITDFKQESNVPVIVGLNSGEGGVFVASLFNETSLQYPELSTDINRLLPILLMYKHVSMPKHFDEITNRIMETYFPSGKIESYSHLDAVKMIGDGTFVTCSLDMSIKLSSPVHFYLFDYKNEFSFNQLYGDCKKPLGVSHADELISLFQSKELNPKGLNDEDTKMSKLMVNIWVKFASSKIPTVDGTDNGQAWPVFTSIGESALLHIDSVQPKLIKNPLAEKYKFWSELPLLSGLNKLISPNNLNIKNEL |
| AcypiCCE10 | MAVAPWSIVAYICLTSAAICYSTAASSSPPPRVRVESGELSGGVEHTLISGRPLYAFLGVPYASPPVYKNRFKEPQPVKPWVGVWNATIAGSDCMGLDHVSFRVVGSEDCLYLNVYTPKLPQEGLISGGLMNVIVYIHGGAFQFGSGIGYGPHYLLDSEDFVYVSINYRLGPLGFASTGDDVLPGNNGLKDQVAALKWIQRNIAAFGGNPGSVTIAGMSAGGASVHYHTLSPMSVGLFNRGIAESGSAFCGWALTENTIQKTKELAESLGCPTYYSKDTVKCLRSRPALAIADSLKNFLPWRYNPFTPFGPTVETGGTERFLTDLPENLPIQNVPLLFSFTEDDGLYPAAEFVSNEETLVDIESNWDEILPFILDYNYTISDELLRSEIAQKIKKFYFGNNTVSVNTKNEVVKMMTDRLFQEPVARTARHLASINTSPVYFYEFSYRGKYSLSDKYSTSGSSQGMGVSHGDDTMYVIKTRYGNPHDNVEDAKLIPVMVSMWSSFAKTGVPDIGSSVVWSPVSKNPSDPLKLIKITQNQTFEQQEYSNPGNYNFWSTLPLTEFPATVKPSKTINRDEL |
| AcypiCCE12 | MSVASWSVVAYICLTSAAICYPTAASSSPPPRVRVDSGELSGGVAHTLISGRPLYAFLGVPYASPPVYENRFKEPQPVKPWMGVWNATIAGSDCLGLDSTFSVVGREDCLYLNVYTPKLPQEGLISGGLMNVVVFIHGGAFQFGSGIGNSPHYLLDSEDFVYVSINYRLGPLGFASTGDDLLPGNNGLKDQVAALKWVQRNIAAFGGNHDSVTIAGMSAGGASVHYHAISPMSEGLFNRGIAESGSAFCGWALTENTIQKTKELAESLGCSTYYSKDIVECLRSRPALAIADSLKNFLVWKYNPFTPFGPTVETGGTGRFLTDLPENLPIQNVPLLFSFTEDEGLYPGAEFISDEEILVDMESNWNEILPFILDYNNTISDESLRSEIAQKIKTFYFGNNTVSVNTKNEVVKMLTDRLFQEPVARAAQYSASINTSPVYFYEFSYRGKYSLADKFSKSGSSQGMGVSHGDDTMYVIKIGYGDPHDNVEDAKLIPVMVNMWLSFAKTGVPDVGSSAVWSPVSKNPSDPLKLIKITQNQTFEQQEYSNPGNHSFWSTLPLTEYHAT |
| AcypiCCE13 | MSVASWSVVAYICLTSAAICYPTAASSSPPPRVRVDSGELSGGVEHTLISGRPLYAFLGVPYASPPVYENRFKEPQPVKPWMGVWNATIAGGDCLGLDSTFSVVGREDCLYLNVYTPKLPQEGQISGDLMNVVVFIHGGVFQFGSGIGYSPHYLLDSEDFVYVSINYRLGPLGFASTGDDLLPGNNGLKDQVAALKWVQRNIATFGGNNDSVTITGMSAGGASVHYHALSPMSEGLFNRGIAESGSAFCGWALTENTIQKTKELAELLGCSTYYSKDIVECLRSRPALAIADSLKNFLVWKYNPLTPFGPTVETGETERFLTDLPENLPIQNVPLLFSFTEDEGLIPGAEFISDEETLVDMESNWNEILPFILDYNNTISDESLRSEIAQKIKTFYFGNNTVSVNTKNEVIKMLTDRLYQEPVARAARHSASINKSPVYFYEFSYRGKYSFADIYSKSGSSQGMGVSHGDDTMYVIKTGYGDPHDNVEDAKLIPVMVNMWLSFAKTGVPDVGSSAVWSPVSKNPSDPLKLIKITQNQTFEQQEYSNPGNHSFWSTLPLTEYHAT |
| AcypiCCE15 | MSKVWRTFIKWRFIATIMFNKFKGYLSNTVVRYLKPRTTVIIEQGTLQGIHYKTQASNKPYVSFLGIPYAKPPVGNLRFKPPVKHPGWSGILKAFSVGNMCMQYSFIKKKIAGNEDCLYLNIFVPQEELKEKKAVMVFIHGGAFNNGSASSDFYSPEYLIDENVIIVTINYRLNALGFLNFDIDECPGNMGLKDQLFAIKWVKANITAFGGDNQNITIFGESAGSASVHCHMLSPQSTGLFQKAIMQSGCLFNSWAFTEKHREAAFKLAKHLGCVKDDPKEIVQFLLSVPAIDIVKLTKIEGITDFQDYEFIPSVESEAISDKFLPAHPEILVKTASTVSVITGINNMEGLIAFGEHKMRKILNDTEEINELFQRNYSKEIINKVKNFYFNECNMECGTTRLENICHLYSDLYFSKDFHRGYDYILKRNISPVYSYEFKFDGEINICKTILFYLRPTFKPLKGACHADELSYLFHGRLFGFAPKANSPELRMCRTMSKLWTNFAKTGNPNSQDLCFEWKNTSAEEPKYLSLDGDNTRMVDGLLNGPRVCFWENIRNS |
| AcypiCCE16 | MSKPWRTFVKWRFITKVIVNKFKRFFSSTVVRYLTPQKKTTVFIEQGSLQGIHYKTQASNKPYVSFLGIPYAKPPVGNLRFKSPVKHPGWSGTLKAFSVGNMSMQYSFLENKIVGSEDCLFLNIFVPLQEEQNEKKAVMVFIHGGVFYNGSGSLDFYSPDYLIDENVIVVTINYRLNALGFLNFDIDECPGNMGLKDQLFAIKWVKANITEFGGDDQNITIFGESAGSASVHCHMLSPLSTGYFQKAIMQSGCMFNVWALNENHRESAFKLANNLGCKKDNPKEVVRYLLNIPAIDIVKSITIDAKTNPDYDFVPSVESEAVTDKFLPAHPEILVKTASTVPVIVGINNMEGLLVFGDENFMKTINGTEEINKLFERKYGYTEEVVNKIKNFYFDERNVECGTTKLEKICHLYGDFYFSKEFHRSYEHSLQQNVTPVYSYEFKFDGKINVFKKMLNFLRPTFEPLKGACHADELSYLFHGRLFGFTPRANSPELRMCRTMSKLWTNFAKTGNPNSHDLKFEWRNTSAEEPKYLSLDGDDTRMVVGLLNSSRMRFWDNISKNIRILQQKSNFDLYYYSPRTTVIIEQGTLQGIHYKTQASNKPYVSFLGIPYAKPPVGNLRFKPPVKHPGWSGILKAFSVGNMCMQYSFIKKKIAGNEDCLYLNIFVPQEELKEKKAVMVFIHGGAFNNGSASSDFYSPEYLIDENVIIVTINYRLNALGFLNFDIDECPGNMGLKDQLFAIKWVKANITAFGGDNQNITIFGESAGSASVHCHMLSPQSTGLFQKAIMQSGCLFNSWAFTEKHREAAFKLAKHLGCVKDDPKEIVQFLLSVPAIDIVKLTKIEGITDFQDYEFIPSVESEAISDKFLPAHPEILVKTASTVSVITGINNMEGLIAFGEHKMRKILNDTEEINELFQRNYSKEIINKVKNFYFNECNMECGTTRLENICHLYSDLYFSKDFHRGYDYILKRNISPVYSYEFKFDGEINICKTILFYLRPTFKPLKGACHADELSYLFHGRLFGFAPKANSPELRMCRTMSKLWTNFAKTGNPNSQDLCFEWKNTSAEEPKYLSLDGDNTRMVDGLLNGPRVCFWENIRNS |
| AcypiCCE17 | MTVVLEQGTLQGLHYKTRLSNKSYVSFLGIPYALPPINDLRFKPPAKHPGWTGIFKAFSCGKVCMQYDVFMTKKIVGSEDCLYLNIFVPQVEEVVEKKAVMVFIHGGAFNYGSGSLDFYSPDYLIDENVIVVTINYRLNVLGFLNFGIDECPGNMGLKDQLFAFKWIKANISAFGGDTNNITIFGESAGSASVHCHLLSPQSTGSFNKAIMQSGCVFNPWAFNERHTEVAFKLAEKLGCQKDDPKEIVKYLLNVPAIDLVKCTTLKIKFEGQRDLLNFQFVPTIESEAVSERFIPAHPDILIKSASAVPLITGTNNMEGMIAFGEHKLGKLFDYHKLEEIRKLFETDYSEEIIQKVKNFYFSEHEQSSDITQLENTCRLFSDVFFTKDFYRGFNSLLKKDGQPIYNYEFKFDGELNACKKLLFATRPIFHSLKGACHADELNYLFYGQLFGFLPKANTPEFRMCKTMSKLWCNFAKTGNPNSSDSNVVWNNTNLDNPKYLSIDGDNTCMVDGLINASSVHFWENIFEIIRLKQKL |
| AcypiCCE18 | MICCNSQHRTQQFPVKMDVVLEQGALQGLQNKTLLSNKPYVSFLGIPYAKPPVNDLRFKAPVKHPGWSGVLEAISEGDKCMQYAFMTDHIIGNEDCLYLNVLVPQVVKTNEPNGKLAVMIFIHGGAFNYGCGSVNEYSPDYLLDENVIVVTLNYRLNVLGFLNLDIDECPGNMGLKDQLFAIKWIKENIAAFGGDADNITIFGESAGSASVHCHTISPQSTGLFQKAIMQSGCALNPWVLNENHRAAAFKLANNLGCLSNDPKEIVKYLKNVPAIDLVKGTKFKDETGFLDYKFVPSIESVTVSNPFLPAHPEQLVNDASPVPVIIGLNNMEGMIALIEHRISQFSDDRITDEISKLCKNRYSTEAITKIKNFYFNKCNIESETTKLEHICHLHSDVLFVKDFYRGFDNFLKQDVTPVYKYEFKFDGELNAVKNMVFATRPVLRHTIKGACHADEINYLFHGKLSGVVPKPNSPELEMCKMMGKMWSNFAKSGDPNSSDLSFKWLNASASAPEYLSIDGDRTRMAEGMINSNRLRLWQEISESVV |
| AcypiCCE20 | MLFRFALALGVLLAFCEALSRQKRIVGGYPSLLPESDGFNVSQPSGNHGNSQNYENIVVQVQDDYRSATVTGVEEPEGYLAYKGIRYAEPPVGRLRFQRPSFVRLSGEVDATKFGSPCPQLSSDGSGLVGSEDCLFLNVFTPLKKLNYSNIPVLIWIHGGGFFSGSALQYGPAHLVKNNIIVVTIQYRLGSLGWLTSNLKHLPGNVGLFDMSAAVQWVKDYINYFNGDPERIVVSGQGSAASAVTLLTMSDFTKGMISGVVAMSGSPLSAFAVDPAPKQTYQNMTTLLGCDQSAPLEAIRCLQMLSIQTIINSDYKYQNTKLGKEGYLTGLGSLLNVGPTVEGYNDGRFLPNFILDSPLRLLKKHKLPKIPMLTGVNKLETKSGILGKYRNQVIDNLNKIPDYINKVLPQKVLNTNTGLFKNGSVNNALKNLFENIDYLNLFTSRLETGIKEITKVMQGTTDALFNLPAFFTSHLWSTRSSAYFYSFEHKPSIKSPGKWFLPNILGNPNKEVSKNDGTNSQHVEDNGEPGHGDELIYLYDVRSIEGKQISGTELKDKKDIEMRNNFTSLVAEFVKNGKPKLKILGDEWPSFTSSKQSDYVVLGENSRIENKFRFCEMGLWGGVPDILQSTYCNLPGITDILKTVPDLLQNGVLGDVTEGAVNLLSLNTIDGLLKNGNNGLLGTNNEGLLGTTNNGLLGINKNGMRGTLSGKKPETTTKTPNLNTQKPTNILSGLGVLG |
| AcypiAce1 | MDQWLLWFGYLVASTYGLSLRHARHQSVGTPTAEEILEPQILIEDTDHVFRQRASDMFAQEPEYTEKRNLNHRRRSEFSGNQDNDFESSGETYSAYKSDDPLVIHTNKGKIRGITQAASTGKLVDAWLGIPYAKKPIGDLRFRHPRPIDRWDKTSPETILNCTTPPNTCVQIFDTLFGDFPGATMWNPNSPVSEDCLYINVVVPKPRPQNAAVMVWIFGGGFYSGSATLDIYDPKVLVSEENVILVSMQYRVASLGFLYFDTEDVPGNAGLFDQLMALQWVHENIKLFGGNPNNVTLFGESAGAVSVSLHLLSPLSRNLFNQAIMESGSSTAPWAILSREESFSRGLKLAKAMGCPDDRNDIHKTVECLRKVNSSAMVEKEWDHVAICFFPFVPVVDGAFLDDYPQKSLSTNNFKKTNILMGSNSEEGYYSIFYYLTELFKKEENVVVSRENFVKAIGQLNPNADAAVKSAIEFEYTDWFSPNDPEKNRNALDKMVGDYQFTCNVNEFAHKYALTGNNVYMYYFKHRSLNNPWPKWTGVMHGDEISYVFGDPLNPNKRYETEEIELSKKMMRYWTNFAKTGNPSKTFEGSWVTPKWPIHTAYGKEFLTLDTNNTSIGVGPRLEQCAFWKNYVPDLTAISKSMKSDKNCTTISGGTKTNMIKLSLWTIVMTTVVLML |
| AcypiAce2 | MSVDCVYTSAVTLLLCCSAVLGRPSSNGGADGGGGGGGGGGAGAGGGGGGGGGGGGGSVDDTDESPVVVTSSGMVQGYTKIIANREVRVYTGIPFAKPPVGPLRFRRPVPVDPWTGVLNATRLPNTCYQERYEYFPGFIGEEMWNPNTKLSEDCLYLNIWIPKKQRTRHHSNNAHHAKIPVLVWIYGGGYMSGTSTLDIYDGDLLAATFDVMIASMQYRLGAFGSLYLTPELPEDSDDAPGNMGLWDQALAIKWIKENAAAFGADPETITLFGESAGGGSVSVHLISPETRGMVRRGIIQSGTVNAPWSYMTGERAVDIAKKLLDDCNCNSTSLNNNPIGTMSCMRSVDASTISKKQWNSYSGILGFPSAPTVDGILLPEHPLDMLAKANFSDIDILIGSNLNEGTYFLLYDFVDFFDRTSATALPKEKFVQIVNVIFKDRTQLERDAIIYQYSGWEKKEVDDKYSNQKQLSDVVADYFFVCPTNLFANIVSSRGARVYYYFFTHRTDSHLWGDWMGVLHGDEMQYVFGHPLNMSLPYNARERDLSIRIMEAFTRFSLTGTPVSDDIDWPLYNESNPIYHVWNAAELHVGYGPRAAECQFWNGFFPKIAQALKETTKITCEDYPDSMPTTNENCTFTSSFATINPRISFTIIFIFVLPAHGLF |
| AcypiNLG-1 | MGGGGTATLALVLVTAAAAVVMVAGGVRPEVLGYTETVWLDRQGPLKGLITTVGGGAEQRLDRVEVYLGVPYAASQERFMPPGESPTWCPKADDGSFDRSHCRPLRAEYLKPVCPQRPPDLLVANKRLSAVRQNYLKRLTSYLGNQSEDCLYLNIYAPHDPKNEMNDEAGNGYKATTKKSKYAVIMFIHGESFEWNSGNPYDGSVLASYGKVIFITINYRVGVLGFLKSNGGDIPASNFGLLDQMAALEWIKNNIQAFGGNPNAVTVMGHGTGAACANFLMMAPPVNNNNLFQRAILMSGSALSDWAWVKNPIMNTIQVGQSLNCEPGGNEKFFECLRRKRFTDLVSTKMYLPPFVTVFGPMVDGVMITNEPLPLLKEHKNLMKNYKILAGVTEYESYHLLDSVSLAHGMLENEQNKVLLDYMKAKFELFPDFTLTATLAQYTDRNRTWSSSGALENRDVLLDILSDAMVLAPLTQLAEIQSEVNENTYFYVFTHRSTFSEYDVVKKSINGEDLPYALGVPLGGNTIHLKTQYDPKEKRLSEMIMTHWSNFAKTGDPNFSKKSYQNEWKMNNVRWPKYNTVNRAYLELNLTNRQRVNYRTSKCKFWNVDLPQRIEDERQKDAEQSKPPYSSLPVIPKESQPPWIDREPIFSLPTLMPDDPMLKPDEFPEEPNPGEEPSAEPSDKTVQETVEASSSFVVSLIIIIGILFLLVNICAFGGIYYQRDKLRVRERLFNNRFRCNGANTSRTFDDDDEQDDVYMKTTAEEPEIANVHKINKNQAYDSVALASVKPEAIGGKRLGKWAEISRQRSSSTITMDPHTRVKEWINTEIIQRYSPRILRRQRREDKKRQLVCAEDPSVMVEKCEVTDAHKAAEKTRNKVSVGIDATPAARTISVLKQTPIELTKSMDAMGHLERVEIKPSLSLGSINKKTLTRSDALNDCDDLLMSSQDTLHKSSTSIQLKPAVKEKRPGIKVTHGYSKSEPARDIPKEPFYTQVCKPKTLAVASSKNASFDSSSHYEDINVTSKEYAMNGGNGSVDCCSDGGGGVRRIKYPKVLPDFPAQCGAGNERRLSLPPPLAGIPEGSGKIPPPPPPRVSSTLGRKKDKADAMTVAGPFNRQPQQDTIPEEHKSLVVPCVPNKVCPAVQVATDMTLTVKKPVEPRIVIKASSVQQNSQHQHPQHLQQQHQQKQPKRTNIPRVVHPQNSDRNNKTTAAGTESDTGTIKRKKK |
| AcypiNLG-2 | LFRRAILLSGSALSSWALVEDPVSYALDLAKQVNCTTDGTGNNPEPIVDCLREVPLDLLMSAGVALPTYLSAFGPSVDGVVVKSNYQEDVITNLLPEFDGYSGSSVAFGARKFQKFEFGSGINKYDLLFGVVTSEALWRFSSNDIQAGFEGDRRDKILRTYVRNAYSYHLSEIFFTIVNEYTDWERTVLHPINTRDATIAALSDAQYVAPLVHTGDMLSQPKTPAADDLNKDRRTKSFFYVFDYQTRDGDYPQRIGTAHGEELPYMFGAPMVTDGMNHFSRNFTKAEALLSDAMILYLGNFARTGDPNDHDDKPDSGSKERNRFRSVTWDEYDPVHQKYLEISMKPRMKNHFRAHQLSVWLRLIPELHRAGMEDVMLRHNLFRNHDDAELYEGVVRPDPLSRRAPQRATVNRFTANGTVAEVVQSLTTTGRPQWPAAEATTCLPPARGSNAANASTSAEDALATLGAAGYAAYSTALHVTIALGVSLLVLNGLLLVIICYQKDRFKCLQHHQQHQHQQTVGGQEDSVRYKAATVPDDRLQQSAAVVVDKAGGYDGRHHHMQHHHHPPAQQMHLAADAVAVDMEQRHHHHHGAAGHHQHHHMHHHHVPAAAGGQHPPLPQTTATMTTTTQQQQLSAATAAVRMAAGHDMTFPKAAKPSSRLLHDHQVGGGSGGCPPNGSLVHGTLPKPPPPPRSSVGVQSQESQPLLVQQGVGIGQMPKTGTLKMPVAAMSEMRV |
| AcypiNRXN-1 | MVLHVNDTREMRVKPATATVLMKRDRTTTVVDGCGRGGGSGGSGGEVAYGGGSGGGVNFGSADKISPKPVLVFIHGESYEWNSGNPYDGTVLASYGGLVVVTINYRLGILGFLNLNSNQHLKSPSNYGLMDQIAALHWIQENIAVFGGDPSNVTLMGHGTGAACVGFLMASSAVPDGKLLFHRAILLSGSPLSPLSLVRDPVYYGHQVSKLVNCSPDLQHLHLLNCLRDTPLDQLLNAQLHVPEFTTAFGPSVDGVIIDVGTAAESSRLGTQSNGQDIGPGRPPEDPKELVLANGFGPAILANPSARQRLLSRLSRFDLMLGVVRAESFFAFTGDDVQYGIEADRRTKILKTYVKNTYKYHLTEILATIVNEYTDWDRPVQHPVNIRDETLEALSDAQVVAPVINTADLHSANRRNSYLFVFDYQTKYGDYQQRQGCIHGEELPYIFGAPLVGGLMHFPRNYTKSEVLLSEAAVIYWSNFARTGNPNEPQDQDMLHKQEHGRFKNIEWTAYETVHKKYLSLDTKPKLKSHYRAHRLSFWLNLVPELHKPGDDVPYYHHMFQSDLRGMTFKPGSERTTSRPSPDVYYRGQPPAIMQQPTTGNKTTSGTSDGGTANGTNVLQPPPVVVASSSSSDRGDGDNDDGGIGSGGRQLKNGPSGDKDDGEGGGRSSSGSDGQDELGGGGGGGGAAYQTFPLALAVAIGIGCSLLFLNIMIFAVIYCNKSAGWGGRRGSSNSSRRTSGAGGDAEDLQQQQQDDRIKKIKKRPENGGPMTNLCSTGGKP |
| AcypiGliotactin | MIETPKFVMLFAQCHALSISLILFVVAISNAQDYRYQTYNGQNYPPQQTAYDRNLPQSANPYNPLLDQYGQQNNYQNRLPYNQPGRPGTPSYRPNGQNYAGDDMLLLPGILGRWRPDLQGRERTDSKQLDRDVFVSTKYGQIQGFKVHLYDNPLPENGYRPFQTPVERKQAEVAVFLGIPYAAPPINEGRFRPPRPHKGWQLHQAVDFGSACPQPAIYTGITKGVPKVDEDCLFLNIFMPSTGVAPAKPYPVMFYIHGGDFVHGSSNSFHGHMMAAFYNVVVVAINYRLGALGFLSTCDDNSPGNYGLMDQAMALRWVYDNIEFFNGDRKSITLFGPDAGAASAGLLMVNPKTSFMVSKVIAQSGSALADWALIKDRWRALNTTKVFAAHIGCSTESTWKIVDCLKRGRSFLELGSDFKPQIGFNAWGPIIDNEFLVPKSDWYDGWQQSDWHFINESVKTAIQSGHFNKDLRYLSGVTTQEAAYILYNNKTYTVTEEFFNNKVKELVLQYNYTLNPSGVYQAIKYLYTYWPDPINPDSIRTQYINMLSDFLFVAPHDEMSKLLVAEDVPVYMYVLNTTVTSIPLPEWRLYSHDTEYYFLTGAPFMDSEFFPRIPVVDKRKWTDNDRNMSHFFMKAYTNFATYGNPSQSQILGIHFDMAFKNDLKYLNINSTYNTTIKQNYRQMESAFWSEYLPTVIGIIVPTYPPTTEFWWEPKTPIQVAFWGLSGLNMILIALTVILCILWCNAKKTKDRYYNGDVLVVREEPMPMSHQGIDNRSVTSNIYEYHDAPIKTLHASPKKTASSSTLRTNSAASMRDHPMAINEKSVPQTQV |

**Table S2.** The amino acid sequences of 48 CYPs used in Figure 3

| **Protein name** | **Protein sequence** |
| --- | --- |
| LeCYP6a13 | MFTENWWINVITPCTIIATIGYYFCVSTFKKWEELNVPYIKPIPLFGNFLNVALSKDHPLEFYNKIYYEFAGHKYGGLFQMRTPYLMVRDPEIINDVLIKDFWYFPDRGIYSDFTANPLSNNLFFMENPQWKTIRNKLTPAFTSGKLKTMYDQIKECGDLLIKNINKDLKEYGNEIEIRDIMGKYSTDVIGTCAFGLKLNAINDDESLFRKYGKSIFTPSLRMLFRELCLMITPVLLKVIRVKDFPTAATEFFHEAFKETLMYRLENKIVRNDFVNCLMQARTDLVLNKDLPEDEKFTESQIVANAFVMFAAGFETVSTTVSYCLYELALNKSIQDKVRKEIQLKLSKNDGEINHEFLMDLNYLDMVIAETLRKYPPLVALFRKASKTYQIPNDSLIIEKDQKIIIPIYAIHYDSKYYTDPEKFIPERFSSEEKAKRPSGIYFPFGDGPRMCIGIFLNV |
| LeCYP18a1 | MTTEIMSDGDGYSRELWLNAVAAALGLTYSAYRQLRVARTLPPGPWGVPFLGYAPFLSNHCTYLKYNELARRYGPICSFTQRGNTVVLLSDHKLIKTAFDMKQITGRPNDGYMDIIGGYGAVNSTGKLWESQRKFLHLVLRHMGMTFTGHNRLNMENRIMIEVSTLMEMFHKTCAKPIDLNAGSLCLAITNVISSLTMSVRFEPNDPRFERYMHMVDEGFKLFGMLRPVSLFLPRRHITDERNIQEKIKNNHREIAGYFQNIIEEHKSTFDPNCIRDLVDAYLLEINRSQEAGTMDQLFQGLDPNRQVQQILGDLFSAGMETIKNTILWAMVYMLHYPDVMTKVQDEIDSVVGQYKSPVLDDYPNLPYTQATLYEVLRKSSITPLGTTHATTSDVTLNGYHIPTGAQIIPLQHFVHNDPNLWDEPEAFKPERFINAEGKVKKPDCFLPFGVGRRKCLGETLAQMELYLFFSTLLHEFDVCLPDGDELPSMDGQVGITLTPQSFKVVMKARKK |
| LeCYP6a2 | MNTPFLMIRDPELINTIMVKDFSYFTDHGFDTDPSINIMANSLFMLNGDRWRTMRQKLSPGFTSGKLKDTHDQIKDCVDQLMEVIDDNLKISDHFELRDMVANFSTDVIGMSAFGLKLDTVKNGNEDFRKFGKKIFQSDFKQLFVQAMLLLCPKLVITLRLQQFPKDAAEFYASMFRDVLEYRDRNNIVRNDVTQTLIQARKDLVTNNDDDDSTSENKWTEIDIIGNAILMFVAGAETVAITMCFCLYQLALNKDVQDKLRKEIVTTKAKSGGQLNNDFLTNLHYINMVLEEVSRMYSITTMIIRRATKNYKVPGQSLVIEKGQRIIIPSYSIHNDPKYYPNPETFDPERFSTEEKAKRLNGTYIPFGDGPRLCIGKRFAELEMKLVLSKLLLKYEVIPCEKTEIPLNIRGPGNIVNPKNGIWLSFKPIVAN |
| LeCYP4c1 | MLWVKVCLLHQVGSIQKYECNAFVQNIYIAVILSGYKEIFYDRILQFKTIIVDKWRRHRRMITPAFNAKLLEQFFPMFNEKNDILIRNVMKEINKTQVFDLWDYIAPFSLDSICQNAVGYNLDMQSNNKECEFGEAIKTGMELNGIRVSKPWLYPEIMFSMYLKLIGQQRIFETVKKFPSQVINEKKNEFDQRKRAINVKKDVINNDDENQSKLFLDILFKLNDEGGNYSDADIRNEVITMMYAGSDTSTIVICFCLLMLAIHQDIQENVYDEIYNIFGESDQTMTIEDTTKLVYLEQVIKETLRMYPVASLMLREIQADLKIFSSDYVLPKGTMCVICPVATHHSSDLYPNPWSFNPENFSPENVDKRHKYSFIPFSGGPRGCIGSKYAMLSMKVTVSTFLRHFSVHTNIKLTDIKLKFGLMMRSVNGYPVTIRPRDRRPTYKRNQNGQGKCAIVG |
| LeCYP6k1 | MISSVTEWLLDNVTCLSLIVVFASFYYYSTSTYGKWRKLNIPYVPPVPLFGNTIRMMMRLEHPIDMFDRFYNSFPDVKLFGFYQMRDPVLLVRDPELINAILVKDFSYFTDHGFDMDPSTSVLANSLFFSNGRRWRTMRQKLSPGFTSGKLKDTHGQINECSDEMVSGIVKTIEKKTDRIDVKTITSGFSTDVIGTCAFGMKLDTIKNDDSDFRRYVKIMFQSTPKQMIVQVLLMICPWVIKVLKIQMFSAEATNFFHKVFTDVFKYREEHNVIRNDLAQTLMQARKELVLKENLTTEDTFTDDDIIGNAILMFTAGSETVSSMISFCLYELALNTEIQDRLRTEICSMKSKHDGQLNNDYLMDLHYINMVLEETARKYSIAFNLMRVATKTYTLPDESFVIEKGQKLIIPLFSIHRDPKYYPNPLSFDPERFSVEQKSQRPNGTYMPFGDGPRLCIGKRFAESEMKLVLSNVLSKFEVLPCEETEIPIDIRSKSGITSPKNGIVLKFRPIAES |
| LeCYP6a14-1 | MSFLSDWLLDNVTYLSLTAVIVSFYYYSTSTYGKWQKLNIPHIPPVPLFGNTLRLILKLEHPSDMFDRIYNRFPGVKLFGFYQMREPMLLVCDPELINTILVKDFSYFTDHGFVLDPSTTVLSKSLFFANGQRWRTMRQKLSPGFTSGKLKDAYWSINECSNKMVSDIVKKLEKTNQLEVKTMTDGFTTDVIGTCAFGLELDTINNDNSEFRQYAKTLFKRTMRQIIVQAITMICPLVINLFKIQMFPVAATNFFHKVFTDVINYREKYNIIRNDLTQTLLQARKELVLKENSAVEDKYTDDDIIGNAILLFVAGSETISSMVSFCLYELALNKEIQDKLRTEILSKKAKHDGQFNNEFLMDLHYTNMVLEETGRKYTISPMILRVATETYTLPDESFVIEKGQKLVIPMFSIHRDPKYYPDPLKFDPERFSIEQKSQRPNGIYLPFGDGPRMCIGKRFAESEMKLVLSNVLSKFEVLPCEQTEIPLSITSEPGLLTPKRDLVLKFRPIVEH |
| LeCYP6a14-2 | MSATQLFLDSVMAGWWTVAVLTLLVAVAYHFSTSTFGYWRDRGVPYAKPTVPLFGNIGGLALGIEHQARMFSRIYDGFRGHRYGGLYQMRTPHLMICDPALVNRVLIGDFTHFTDHGMYAASPEENPLANGLFNMNGAQWKIMRQKLSPVFTAGKLRHMRGQVAECSEQLMRNVAADVPAGGGQMEIRDVLGKYSTDVIGTCAFGLHLNAINDERSSFRKYGKAVFAPSFRVLLKELAWMVTPALRRALRISDIPPDAAQFFTAAFTDTMKYREEHGIVRDDVMQSLIQARTDLVVNKTEPSVEFLETDIVANAFILFAAGFETVSTAMSFCLYELALKKPIQDKVREEMNKMKNKHNAEIDNDFLKDLHYLEMVLAETLRKYPPLITLFREATKDYQVPDDTFIIEKGTKVLIPAYAIHHDYRYYPDPETFDPERFSPEEKAKRPNGTYMPFGDGPRLCIGKRFAEMEMKLALTELLTKYEVEPCEKTDIPMRFSSRSLIIMPENGIWLKFKPIHTSK |
| ApCYP306a1 | MFWIIGVILFGVLCAGYLWRSNRNLPPGPWGVPIFGYLPWLNPTEPYKTLTALASKYGPIYSIQMGKHFAVVMSDPTLVRMALARNELADRTNFEVVNEIMQEHGLIFTHGPLWKEQRKFVCNWLKVIGVTKFGDKKNNLQLLIADAVSTTISKLRQSNNRPIDTGTFFLVHIGDFINLIVLGKAWPEDDPNWIYLRNLAEDGSKKFAIATPLSVLPILKIIPKYRNTVFEVIEGVKNTHLIYKTLMEKRGNEIHESDDLMAMFMKEMTKRKNDKDSHYFTEKQCCFLLSDLFGAGVETTVNTLRWFLLYMALNQEIQNDLQKLLDSACTDGGLIGLEQIESIPLLKACVSETMRLRPVAPSGIPRSVNTEITISGYRIPKGTMVLPLQWAMHHDEKYWTDPETFRPKRFLDDEGNMINHKAFMPFQAGKRACVGDTLSYWILYLFGANIIHNFNVSAEQGLSEKEINTIMDGEFGITLSPATHKVVFKSRI |
| ApCYP15a1 | MFFVAVVISVFIIVCILDIITPHKYPIGPTRVPLLGNYLEIRKLRNKLGFYHLVWDHLAKYYGKVFSVKLGRIEAVVVSGYDAVRQVLCKDDFDGRPDGFFFRFRAFYKRLGIVFVDGPTWTEQRKFCMQHLRKMGFGGDLMERIIIEEVNDLMLDISRKCENGKPIEVYGLFDVSVLNGLWAMLAGHRFALNDSRLARLMELVHVSFRMLDMSGGILNQMPFIRFFAPKCSGYKYLKQIINEFYTFLKESVEEHKCRANDQEDDFISAFLKEIEKNKESPGSFSEEQLLVILLDLFLAGSETTSSMLSFVILLLLKHQDIQAKVHAELDAVVGDREIHLADKNRLNYLEAVLMEVQRHSNVAPLAIAHRTIRKTSLQEYTIPKDTLVLASIWSVHMDEQHWGDPKVFRPERFLDSSGKIINDSWFMPFGVGRRRCLGEILAKTNIFMFIAKLIQHFEIRIPQGAQLPDKPQDGVTISPSPFSAIFIPRRCLSQ |
| ApCYP305a1 | MSLLLMAWYFVCFVTVILLLIALRTCRKPKNYPPGPKWIPFVGNTYQLSKLAATKNGQYLAFEELRQRYKSDIIGLKLGREYVVIVFGNDLLNETFHRDEFQGRPDNFFMRLRTMGKRRGITMTDGDLWKVHRSFAVRHLKLLGLGQRRVDELIHDEYQLMVDRLFDATKSVTPTLYLQSAVMNVLWELTAGTKFEDPKLLTLMRKRSSAFDMAGGLLNQIPWLRYLAPTRTGFSLITEINQQLYSLISNIIVEHKKTITHTTRDFIDAYLNQMKKEEIYNTMFTEEQLIAVCLDLFIAGSSTTSSTLDFAILAMARWPDVQAKVQSTLDEIQPPGTYITAEQILKNRYVEAVLLETKRLNHVTPIIGPRRVLRNTNLNGYNIPKNTTILMSLYSVHQDQLKWGDPEVFRPERFMDTNGKINTTEDMYFFGFGKRRCPGEALAQRFVNLAFANLIHDFTIEIDQLPDGVNCGILLTPKPYKIKMTKRK |
| ApCYP307a1 | MDTAKGVVAAAADNVTVVLLLLLSVVLLILAVKSASGRGPWTSRRRPGKSTAAVALTAVPDGPTAYPVIGALHAMDGHRDKPFHRFTELSHKYGPVFSMTMGSMPCVIVNDFDSIKEVLITNGSKFGGRPDFSRYNVLFAGDRNNSLALCDWSWLQETRRKIARKYCSPKVCSSNYGLLDSISSDELDVFLESLAAVTIRGFECEVQLKKQLLMACANMFIRFMCSTQFEYGDPKFQNMVRTFDEIFWDINQGYAVDFLPWLKPFYAGHMRKLSKWSTQIRRFIMDTVVSKRISYAKAAALGSPGDYNDYAADDVDEQEPIDFTDALLMSLRKEPGLKMNHVLFELEDFIGGHSAVGNMIMLALSMVATRPHVAQAIRDEAEQVTGGQRLVRLYDKPDMPYTEATLFETLRFISSPIVPHVATEDTTIKGFKISKGTCIIINNYEINTSPAYWDNPEVFDPNRFVHRESGTKPCIRKPEYFLPFSTGKRTCIGQQLVSGFGFVLLAGILQRYEVKATAQLAIPEARLALPPDTYPLILKPLDGSR |
| ApCYP18a1 | MTAETMSDGDGYSRELWLNAVAAALGLTYSAYRQLRAARTLPPGPWGVPFLGYAPFLSNHCTYLKYNELARRYGPICSFTQRGNTVILLSDHKLIKTAFDMKQITGRPNDGYMDIIGGYGAVNSTGKLWESQRKFLHLVLRHMGMTFTGHNRLNMENRIMIEVSTLTETFHKTCGKPIDLNAGSLCLAITNVISSLTMSVRFEPNDPRFERYMHMVDEGFKLFGMLRPVSLFLPRRHITDERNIQEKIKNNHQEIAKYFQSIIEEHRSTFDPNSIRDLVDAYLLEIKRSQEAGTMDQLFQGLDPNRQVQQILGDLFSAGMETIKNTILWAMVYMLHYPDVMTKVQDEIDSVVGQYKSPVLDDYPNLPYTQATLYEVLRKSSITPLGTTHATTSDVTLNGYHIPTGAQIIPLQHFVHNDPNLWDEPEAFKPERFINAEGKVKKPDCFLPFGVGRRKCLGETLAQMELYLFFSTLLHEFDVCLPDGDELPSMDGQVGITLTPQSFKVVMKARNK |
| ApCYP303a1 | MWILVLVLFSVVVALLSYLDMRKPKNYPPGPKWLPILGSALTVNSLRKQTGYLYRATICLAESYGPIVGLKVGKDRQVVCCGYNAIKEMLTKEEFDGRPQGPFYETRTWGTRRGLLLTDEEFWVEQRRFVLRHLREFGFGKRTMAELVQDEAVQLVEDFKEKIAMSKNGNGEIFEMRDAFSVGVLNTLWSMMASKRYNADDIELKNLQALLTELFANIDMVGALFSQFPVLRFIAPEASGYKSFVNIHQQVWKFLKAELDDHKETFIINQPRDLMDVYLQMLHSEDKKESYSESQLLAICMDMFMAGSETTSKSLGFGFLYLLLNPEVQKKAQEEIDRVVGRDRLPTLNDRPNMPYLEALVLESVRVFMGRTFSIPHRALKDTTLQGYHIPKDTMVIANFAALLNDDDVWDHPDRFWPERFIGCDGKLIVPDEYLPFGYGKHRCMGQTLARSNIFLFSACLLQNFDFSVPDGQAPPSTLGVDGVTPSPGEFNAYVSLRPR |
| ApCYP307b1 | MEFVFSSLTYLLLFVLTAVLLFLIRDELKTKQVDHRAGLVDPPAPKAWPIIGHLYLMARYKVPYRVFDEIMADLGSVFRLDLGSVPCVVVNGLNNIREVLMIKGDHFDSRPSFRRFNQLFKGDKNNSLAFCDWSQLQKTRRELLRAHTFPNTTSNMYTRLDTCLKTELADLTDTLDTMANTECVDIKNMLLHTCANVFMSYFCSTRFSRSYDKFREFIRNFDDVFYEVNQGAPCDFLPSLMPLYHWHFKKIRSWSSKIRNFMETEIFNKRKAAWVPGTKPVDFVDNLLDAVTQPDRDDGFDMDIGLFSLEDIIGGHSAITNFIVKTLGFLVDRPDVQRRIQEESDAVVRASGSVGLSDRSQMPYTEAVVYESLRLIASPIVPHLANRDTSVDGVRIRKGTTVFLNNYSLHMSPELWNNPEHYSPERFINAEGRLEKPEYFIPFSGGKRSCMGYKLVQLLSFCTISTLLNKYTLLPVEDVSYAVPKGNLALPFVTFPFRLRPRNFRKQ |
| ApCYP6k1 | MISFLIDCLVNNVTCLSLIVIFTGSFYYYSTSTYNKWRKLKIPYVPPVPLFGNTFRMLARLEHPIDTFDKIYNHFPDFKLFGFYQMREPMLLVRDPELINMILVKDFLYFTDHGVDIDPSMSTLAKSLFFANGQKWRTMRQKLSPGFTSGKLKGTYCQINECSDEMVSSIVEAIGKKTDRIELKTITGRFSTDVIATCAFGLKLDSIKNGDSEFRRYVKILFQTTTKQAIILILSLICPRVVKILRLQFFSLEATNFFSKVFADVIKYREDHNVSRNDITQTLIEARKELVLKEISTTEDKFTDDDIIGNAIFLFSAGSETISSLVCFCLYELALNKEIQDKLRAEIYSMKAKHNGKLNNDYLVDLRYTNMVLEETGRKYSIAFNITRVATKTYTLPDESFVIEKGQKLIIPMFNIHRDPKYYPDPLRFDPERFSMEQKSQRPNGTYIPFGDGPRLCIGKRFAEAEMKLVLSKVLSKFEVQPCEQTEIPLDIRSGSGLLSPKNGLVLKFKPIIEH |
| ApCYP6a13 | MTMFTASWWINVITPCTIIVTITYYFCVSTFKKWEKLNVPYIKPIPLFGNFLNVALGKNHPLEFYNKIYHEFAGQKYAGVFQMRTPYLMVRDPEIINDVMIKNFSSFPDRGIYSDFVAEPLTNNLLLMENPQWKIIRNKLTPAFTAGKLKTMYDQIKECGDELMKNIDIDLNRTSNEIEVKDIMGKYSTDVIGTCAFGLKLNAINDDESPFRKYGKLIFKPSLRVLMRELCVMITPALLKVVRLKKFPTAATDFFHAAFNETMTYRLENNIVRNDFVHYLMQARNDLVLNTDLPKHEKFAESQIVANAFVLFAAGFETVSSAISYCLYELALNKSIQDRVRKEIQLQLSKNNGQINHELLIDLNYLDMVIAETLRKYPPLVALFRKASQTYRVPNSSLIIEKGQKIIIPIYAIHYDNKYYSDPEKFIPERFSAEEKAKRPSGVYLPFGDGPRICIGKRFAEMEIKLAFVEILTKFEVFPCEKTEIPLKYSKKVLTLVSKHGIWLRFKRIN |
| ApCYP6a13-2 | MFSFIFIWWINIITPCLFIFTITYYFCTLTYSKWEKINVPYIQPIPLFGNFLDVALGMQHPIDFYRKIYYELAGYKYGGLFQMRTPYLMIRDPEIINNVLIKDFSNFPNRGIYSDFSANPLSNQLFFMENPQWKIIRKILSPAFTSGKLKLMYDQIKECGDELMKNIHKNLTKTDNKMEVRDILGKYSTDVIGTCIFGLKLNAVSDDNSTFRKYGKSLFLPSLRTHLRELSLMITPALLNILRFKDFPADATEFFHSAFHETITYREKNNIVRNDFVQTLIQARNDLVLNKNIPQRERFLESQIVANAFVMFAAGFETVSTAISFCLYELSLKKHIQDKVREEINLKLSKNNGLINNDLLIDLNYLDMVLAETLRKYPPTFALFRKASQTYHVPNDSLTIEKDQKVIIPIYSLHYDPKYFADPEVFDPERFSPEEKSKRISGTYLPFGDGPRICIGKRFAELEMKLALVEILTKFETEPCERTEVPIRFSKKALITMPENGIWLTFKKITNQ |
| ApCYP6k1-2 | MMYFLTDWLLDNFTYLSLIAVFTGFYYYSTSTYGKWQKLNIPYIPPVPLFGNAFRMVTKLECPMDMYDRLYKQFPDVKLLGFYQMTEPMLLIRDPELINAILIKDFPYFTDHGFVMDPSTTVMAKSLFFSNGQRWRKMRQKLSPGFTSGKLRDTYLAINECSNQMVSSIVEKLGKTDRLAIRSIISGFSNDVIGMCAFGIQLDSMNNEDSDFRRYSERIFEKTTKQIIVQAVTTIFPFVINLFKIQMFSAEATNFFRKVFADVINYREKNNIVRNDLTQTLLQARKELVLKENSTAEDQFTDDDIIGNAIVLFAAGAETISSIVSFCLYELALNKEIQDKMRAEICSMKAKHDGQFNNDFLMDLRYTNMVLEETGRKYSIASILMREATKTYTLPDESFVIEKGQKLIIPMFSIHRDPKYYPDPLIFDPERFSKEQKSQRPNGIYMPFGDGPRMCMGKRFAELEMKLVLSNVLSKFEVLPCEETEIPLEITDETGVIAPKRDLVLKFRPIIED |
| ApCYP6a13-3 | MFTANWWINVITPCTIIVTIAYYFCVSTFKKWEKLNVPYIKPIPLFGNFLNVAVGKDHPLEFYGKIYNEFAAHKYGGLYQMRTPYLMVRDPEIINDMLIKDFSSFPDRGIYSDFVANPLSNGLFFMENPQWKIIRNKLTPAFTSGKLKTMYDQIKECGDELMKTIDIELIKNGKEIEVRDIMGKYSTDVIGTCAFGLKLNAINDDESPFRKHGKSIFTPSLRSLFRELCLMVTPALLKVVRVKDFPTDATDFFHAVFKETITYRLENKIVRNDFVQCLIQARNDLVLNADLPNHEKFTESQIVANAFGMFAAGFETVSSTISYCLYELALNKSIQDRLRKEIQLKLSKNDGQINPEFLMDLNYLDMVIAETLRKYPPLVALFRKASQKYRLPNDSLIIEKGQKIIIPIYALHYDNKYFTDPENFIPERFSAEEKAKRPNGIYLPFGDGPRICIGKRFAEMEMKLAFVEMLTKFEVFPCDKTDIPLKYSNNVITLVPKHGIWLTFKRIN |
| ApCYP6a13-4 | MFTYYYYYGLTNKYGDLETIPWEKQETPSKHDSHNVIITRRHLTECSLCLHLSRTATALVCPIKECSVDSCLSRVCCVSLKRSRAMFTANWWINVITPCTIIVTIAYYFCVSTFKRWEKLNVPYIKPIPLFGNFLNIALGKDHPLEFYNKIYYEFAGRKYGGLFQMRTPYLMVRDPEIINDVMIKDFSSFPDRGIYSDFTANPLSNNLFFMENPQWKTIRNKLSPAFTSGKLKTMYDQIKKCGDELMKNIDIDLNKNGNEIEVRDILGKYSTDVIGTCAFGLKLNAISDDESPFRKYGKSIFTPSLRMLFRELCLMITPALLKVIRVKDFPTAATDFFHAAFKETMTYRIENKIVRNDFVHCLMQARNDLVLNTDLPKHEKFTETQIVANAFVMFAAGFETVSTTVSYSLYELALDKSIQDRAREEIQLKLSKNDGQINHEFLMDLNYLDMVIAETLRKYPPLVALFRKASQTYRIPNDSLIIEKGQKIIIPIYAIHYDTKYYPEPEKFIPERFSVEEKAKRPSGIYLPFGDGPRMCIGKRFAEMEMKLAFVEILTKFEVFPCEKTEVPLKYSNKVLTLMPKHGIWLRFNRIN |
| ApCYP6a13-5 | MLIFANFWIDFIILITVLFSIIYYYCTSTFNVWKKLNVPYIRPIPLFGNYLRVALGIENPMETYRKIYCELAGFKYGGMFQMRTPYLMIRDPEIINNILIKDFSYFTDRGIYVDFKTEPLSEVLFLMNNPRWKKFRSKLSPAFSSGKLKQMFNQIEKCGHDMINNIFAELKKNPNDIDMRDVVSKYSMDVIGSCAFGLTLNVASDDTSLFRKYGKTAFGPSILYFIREICVMISPAILKILRLTFFPSKTTAFFGSVFKETKTYREQNNVLRNDIVHALIQAHQSDENSSKDETLMESQILSNAFGFFAAGFDTTSTSISYCLYELALKKNIQDRVREEIKLTKSKYNGVIDNEFLNDLHYLDMVIAESLRKYPLMFALFRVATKTYRVPNDSLIIEKGQKIIIPTFSLHYDPKYFSDPEVFNPERFSPKEKAMRPNGVYLPFGDGPRHCIGKRFAEMEMKLALVEILSKFEVEPSEKTMIPVQFSKLSVVVIPRDEKILLKLNPLSE |
| ApCYP6a14 | MSASQLLVDLAAGWWTVAVLALLAATVYHFCTSTFGYWRDRGVPYVRPTVPLFGNIGGLALGVEHQARMFGRIYDGFRGQRYGGFFQMRTPHLMVCDPALVNRVLIGDFAHFTDHGMYTAGPDENPLANGLFNMNGAQWKIMRQKLSPVFTAGKLRHMRGQVTECSEQLMRNVAADVPTGGGQMEIRDVLGKYSTDVIGTCAFGLHLNAINDERSSFRKHGKAVFAPSFRVLLKELAWMVTPALRRALRIGDMPPDAAQFFTAAFTDTMKYREEHGIVRDDFMQSLIQARTDLVVNKTEPSVEFLETDIVANAFILFAAGFETVSTAMSFCLYELALKKPIQDKVREEMNTTKKKHNAEIDNDFLKDLHYLEMVLAETLRKYPPLLTLFREATQDYQVPDDTFVIEKGTKVLIPAYAIHHDYRYYPDPETFDPERFSPEEKAKRPNGTYMPFGDGPRLCIGKRFAEMEMKLALTELLTTYEVEPCEKTDIPMRFSKRSLIITPENGIWLKFKPIHTSK |
| ApCYP6a13-6 | MISYLTNLLFDYIFLSLIIVCTFLYYYTTSTYDTWRKLNVPFAKPVPFFGNIFKMFTGLERQVDAFGRIYQQFPDEKFCGFYQMSTPFLMLRDPELINTVIIKDFSYFTDHGIDMNPSVNVMARSLFFATGQKWKTMRQKLSPGFTSGKLKGTHEQIRECSDQLTNCIYEKSQKTDAIEVYELVGNTATDVIGTCAFGMKLDTINNDNSSFRQNVKKVFKPSGKVIFAQILGVLFPKIVKFLKLQTSPVDVDAVNFFHSVFGEVIEYRTKNDVVRNDLTQTLMKARQDLVVSSDYKGEEKYCELDIIANAMLLFTAGSETVTATASFCFYELALNKVIQDRLRDEIISSKIKHGGQLNNEFLEDLHYADMVLDETHRKYTIITALLRGATQNYNVPGESLLIEKGQKILIPIYSIHHDPKYYPNPETFDPERFTAEEKSKRPNGTFLPFGDGPRHCIGKRFAELELKLILSKILTKFEISPCEKTEIPLQMNKERGITSPKNGIWLNFRPIVE |
| ApCYP6a2 | MIDVISCSIIGLLSSVYILYATVFLSIAYYLCTSTHDKWRKLNVPYTKPLPLFGNSMNLVLAREHPMDFFTGLYNRFPDEKLCGFYQMTTPFLMIRDPKLINNIMVRDFSYFTDHGFDTDPSVNILANSLFMLNGDRWRTMRQKLSPGFTSGKLKDTHDQIKECTDQLINIVDDNLKVSDHFEIRELVGNFSTDVIGMSAFGLKLDTIRNGNLDFRKFGKKIFQSDFKQLFVQAMMLFCPKLVTILKLKQFPDDAADFYGSMFRDVLEYRDRNNVIRNDVTQTLIQAKKDLVTNNDGDDSTSKNKWTEMDIVGNAILMFVAGAETVSITICFCLYQLALNKDIQDKLREEIVTTNAKHGGQLNNDFLTNLHYMNMVLEEVSRMYSITMILFRQATKNYEVPGQSLVIEKGQKIIIPAYCIHNDPKYYPNPGTFDPERFSTEEKAKRLNGTYIPFGDGPRLCIGKRFAELEMKLVLSKILLKYEVLPCEKTEVPINIRGAGSIVNPKNGVWLSFKPIVAN |
| ApCYP6k1-3 | MISFMTDWLHDNVTCLSLIAVLASFYYYSTSTYGKWRILNIPYVPPVPLFGNTTRMMLRLEHPIDMFERFYNSFPDVKLFGFYQMRDPVLLVRDPELINAILVKDFSYFTDHGIDLDSSTSVLANSLFFANGQKWRTMRQKLSPGFTSGKLKDTHGQINECSDEMVSGIVESIKKKTDQIDVKTITGGFSTDVIGTCAFGMKLDTIKNDDSDFRRYVKIMFQSTPKQMIVQVLLMICPWVIKVLKINMFSVEATNFFHNVFTDVFKYREEHNVIRNDLTQTLMQARKELVLKENSSIEDKFTDADIIGNAILMFTAGSETISSMLSFCLYELALNIEIQDRLRSEICSMKAKHDGHLNNDYLMDLYYTNMVLEETARKYSIAFNLMRVATKTYTLPDESFVIEKGQKLIIPMFSIHRDPKYYPDPLRFDPERFSTEQKSQRPNGIYMPFGDGPRLCIGKRFAESEMKLVLSNVLSKFEVLPCEKTEIPVNIRSMSGFITPKNGIVLKFRPIVEH |
| ApCYP6a13-7 | MISCLIYVLFGTPAIAAVAVLAAILYYYTTNTYDKWLKLKVPHDPPWPLVGNTAKMMTLIEHQLTTIDGIYKRFSGEKYCGFYQMKTPFLMIRDPELINNILIKDFSNFADRGFHKDPALNIIANGLFFMEGPKWKMMRQKLSPGFTSGKLKLAHNQIAECSDELMRFIAAKMKENDQIEVKETMSKYSTDVIGTCAFGLKLDTVKNEGSDFRLYGRKILKLSFRFLLAEMVSPKILKLLGVAEFPPDASAFYESAFKEVIRYREENGIVRHDVAQSLIEARKELVLDSTDENGFTEQHIIANAILMFLAGFETVSSTLSFCLYHLALNQDVQEKIRDEMNSKLKQHGKINNDFLVNLHYTDMVLAETERMYVVTNALFREAVKTYHVPGDTLVIEKGTKIMIPIYSIHHDPTYYPEPYIFDPQRFSPEEKAKRQSSTYLPFGDGPRFCIGKRFAELEMKMVLSQIITTFRILPCEKTEVPLKLQNGLPMMVAKNGIWLRFQSISE |
| ApCYP6a13-8 | MFEFVYELFDLKMLLVTAFLGAIYVYSTWTHSHWSKLGISSPSAPVPLFGHAMPSMLGQMHFMDVLHNLYKELGDQRFGGIYTMRTPQLLVKDPELIGHILIKDFNNFTDRGLYAGTHTNPLNNNIFFTRGERWKTMRQKLSPTFTANKLKYMNEQVKECSDGLLSTIGKNLDDDAGRIEIREMMAKYSTDVIGSCAFGLKLDAINDPDSEFRKHGKTVFQPSLRSKIRVAVIFMQPSLLSIFRVHHYSHRTIRFFHDAFQQTIEYREKHNEDRKDFVQHLMKAREDLVLNPNLKPEEKFTEMDIVANAYILFIAGFETVSTSMSFCMYELALRKDVQDKVRKEILEVKSKYNGQMNSECLNELHYMGMVIKETLRKYPPLVTLNRVVTKPYVIPGTQIKLKIGTKIVVPVHAIHYDPKYYSDPEAFEPDRFSDENIHNIQPNTYMPFGDGPRFCIGKRFAEFEMKMALSEVLTNYEVMACDKTQIPIKYVIGSFVNIPESVWLKFRKVNT |
| ApCYP6a14-2 | MFPAVVIIVACCTTVILFLYKYTTYTYKYWKSKSVTFATPVPLFGNIKDHVTLKMTQGECLKNIYNDFPREKFVGMYQLQTPTLLLRDPETIRLFLVKSFAHFTDRGFSYDGHREPLTKHLVNLEGDTWKILRQKLTPTFSSGKIKSMLGLLQGCGVQLIEYMDATIESGKTEFEIRDLTAKFTTDVIGTCAFGLECNSLKDSQSEFRRMGCAVLNSSASLALAKMVRVFFPKLFKALKLRTFPAEVQQFFMGIVKQTIDFRNTNRVRRNDFIQLLLEIKNQNHNQENAIKSIELTEELIAAQVFVFFLAGFETSSTTLSFCLHEMAVNQDIQNRVYDEINETANMYGLPFSYEAISSMNYLEQCLKETMRKYPPVQALARVCTKQFRVPGTDLDLDVGTAVLIPVYAIHHDPQYYPEPDTFNPDRFAKDGDGGGGDNGRPSGVFLPFGDGPRICIGMRFAMLEMKLALAQFLHRYLVTLSDKSCTRIEFEPASFLSCPKGGIWLNVNKRKA |
| ApCYP4c1 | MIFSNVIGALTSDSNTQWMALLSLVVLGVYFLFSDRFSENRGRQISLLPSITRSQWTSLILSLKLASFGPRDILPYFDNVIKKYGSLIHLKIIARHYIIINDPDDIKVLLSSVQHITKGPDYEMLEPWLNKGLLTSTDQKWHSRRKLLTNTFHFKILETYVPSLNKHSRSLVKNLINASDNGKSIADIDSHVTLCALDIVCETIMGVNLRTQEGKSMNYVKAIKNVSQILIKRIFTFWYWNEIVFNLSSIGREFRKSLKLLHDFTENVIRERRKILENVEQKKVDENGKKRIYSFLDLLVGVSKENPGAMTDKDIREEVDTFLFEGHDTSSIAITMAIIHLGLDQNIQNLVRDELYEIFGDSDRDATMEDLKAMTNLERVIKETMRLYPSVTGITRTLKQPLHLDKYTIPSKSVMVVVPHLLHRDKNIYPNPEKFDPDRFLPEQCNGRHPYAYIPFSAGPRNCIGQKFAMYQMKTVLSTILRYTNVETLGTQKSIVISTQLILRADYLPSVKITPITNTTRHIIL |
| ApCYP4c1-2 | MEVSQDFPVSSLKHSAGGPRMTSTELTAYGVISFIVVLWCHYKWNRRHFERLASKMTGPPAYPIIGAGLEFVGTPQQVIERIIKLFDIYGSEPFKVWMGTSLGVTISKPEDVQIVLNSSKALEKDQFYKFFKNTVGEGLFSAPVHKWRRHRRLITPVFNANLLDQFFPVFNEKNRILTRNLKKELGKTQPFDLWDYIADTTLDIICQTAMGYNLDTQLNNESEFAEALTKASELDSMRIYKPWLHPDIIFSIYGKLTGLHNVYKTLHKLPNQVIKEMKETYAQRKIDNKSNTIDVNDDDKKRLKVFLDTLLDLNEAGANFSDEELRDEVVTMMIGGSETSAITLCFCLLLLAIHPEIQDKVYDEIYEVLGDGDQTITIEDTTKLVYLEQCLRETLRLYPIGPLLLRQLQDDVKIFSGDHTLPKGTTCIISPICTHHIPELYPNPWSFNPDNFDAENVSKRHKFSFIAFSGGPRGCIGSKYAMLSMKVLVSTFLRNYSVHTNVKLSDIKLKLDLLMRSANGYPVTIRPRDRRPTYKKNTHCSTVNL |
| ApCYP4c1-3 | MFHEHAPERNKESILSSLKNCMDPSGGSLLEISSGSGQHISYFAAHFPNIEFQPTEINRRLFETINACTHNLSNVLPAKYLDVSSDPSVWLGGQLMNTQYDYILNINTLHVSNFKCTEGLFRGSCCALKPKETGAIIMQSVGEFRLAVSEVLLYSAIISVVVFWCSCKWNNRHINKLDSKMKGPPAYPIIGSALELLGTPEQVINVLLGFYNNYGSEPFKVWLGPFFGVYIIKPEDVQIVLNNSKALQKDRFYEFIKNIFGEGLLTAPVDKWRKHRRLITPLFNANLLSQFFPVFNEKNKILIRNLKKELGKTQPFDLWDYIAPTTLNLICQNAMGYNLDSHSQCGSEFEKAMIKASELDSIRIYKPWLFPNIFFSLFLRLQGQSNVFKTLKKLPLKMINEKKEVFAQKKIVKETIVMNNTDGEKKNLKVFLDTLFELNETGANFSDNDILDEVVTMMIGGSETSAITLCFSLLLLAIHPDIQNKVYDEIYDVLGDGDQTITTEDTIKLVYLEQVLKETLRLFPVLPLVIRKLQDDVKIISGNHLLPKGTTCYIAPLFTHRDCDSYPNPLNFNPENFSQENISKRHKYSFIAFSGGPRGCIGSKYAMLSMKVMMSMFLRNYSVHTNCKFNDIKLKLDLLLRSANGYPVFIQSRDRRPSYKLNKT |
| ApCYP4g15 | MVTNVQGVNPLFALSAFNLFFYLLTPAIVLWYIYFRMSRKQLYDLASKIPGSEGLPLLGNALDFMQDPHTIFEKIYERSFEFEKNSPIKMWIGPRLLVFLTDPRDVEVILSSNVYIDKSPEYRLFEPWLGNGLLISTGDKWRAHRKLIAPTFHLNVLKSFVTLFNVNSRDTVSKLRKMGSSTFDIHDFMSECTVEILLETAMGVSKKTQKKSGFEYAAAVMKMCDILHMRHTNLWLKPDFIFNFTKYAKEQVGLLDLIHGLTNNVLAKKKEEFLKKKSLMKEVSDIPAASEEIVETSSTLEVEEVPYGNSFGQSAGLKDDLDVEDDGIGEKKRVAFLDLLIECSENGVVLSDEEVREQVDTIMFEGHDTTAAGSSFFLCLMGAHQDVQQKVVDELYSIFGDSDRPVTFQDTLQMKYMERCIMETLRMYPPVPIISRQIKEKVKLASRDITLPVGATIVIATFKIHRNEDVFPNPEVFNPDNFLPEKSASRHYYAYVPFSAGPRSCVGRKYAMLKLKIILSTILRNFKINSNLTEKDWKLQADIILKRTDGFKLSLEPRKSLAKTAA |
| ApCYP4c1-4 | MFAQMRMAIHNAAHALPMTKSELYFYASIVIFVVLWCRMRWQYRQFYRLADKLKGPPSYPLKGSIFDLSTTPEKLMYNFKESAEKYNYEPVKLWVGPFFFVGVYKPEDVQIVLNSSKALEKGMIYHIIRHAVGEGVFTAPMGKWKKHRRVIASIFSSKFLDQLYPIFNENNKKLVENISKHVGETQPFDIWDYIISCNLNNVSQAAMGYNLNDQRTLSEFVLAMKKVSELSKCIVKPWLYIDQIFAVYTYLTGLNVYMSQLNRVSLQIIRDKKLEFKSIKLQQSTDKSHEVVPEKKRNSTKVFLDKLLKLNDEGADFTDEDLKDEVITMTVAGSDTSAISECFCILLLAMHQDIQDKVYDEIYSVLGDSDREVIPEDIFRFKYLEMVLKESLRLFPPGAIFSRKINENVKLTNFELPKGSNVFVSPYVTHRCPQLYPNPDTFNPENFSAENEANRHKFSFLAFSGGPRGCLGVKYAMISMKLMMVAVLRRYSVHTDCKLSEIEMQIDLLAKKANGYPITIRPRERTQDR |
| ApCYP4c1-5 | MVSGGFNFTITKSFIPIFYEESNVLDDILKQKCDLKSNECDISVPVSMATMEIIGRTALGVKFNAQNGGRHRFVENLQTVMHAWEYRVTHPWYLSKTLFQLSSVKKKHDQSQKIINEFTDEIIKKKLDELNQNANNKNKVETDDEDVCRKTKTVIDILLENYHEMSHEQIRDELGTIMIGGQETTAMANACAIFMLAHHPDVQNKVFEELQSIFSTGDGDHSRPLTYEDLQQMEYLERVIKETLRIFPPLPVFCRSLDEEMKIGEHMCPAGSTLLVSPLFIHSSGQYYTDPEKFNPDNFLPDTCHSRHPYSFIPFSAGYRNCIGIKYSMLQMKTVISTLVRKNTFSPSERCPTPKHLRVMFLATLKFVDGCYVKIVPRTS |
| ApCYP4c1-6 | MIEVNFYSVVLMPLAGLISYAIWSRLRMPVEYRQISSHVPSATKTFWSEMVLSWKLAMMQPKDILPFLTDLIRNNGPVVHFNLSGRSYVLLNDPDDLKILLSNTQNIKKGPEYEMLKPWLNEGLLLSSGQKWHNRRKLLTNTFHFKTLDMYNHSINKHSRILVDKLLDASANSNKEISIADYVTLCSLDIICETIMGTEMNAQEGKSVQYVHSIKCACKSVIERIFKFWLWNDLIYKISGSGQSFFKSIKALHEFTDNVIKSKRALLNNSGIEEMQSDSKFEKTKKKSFLDLLLNVLNDTPDQMNDRDIREEVDTFLFEGHDTSSISMTMTLVLLGMYPDIQDRARDELHSIFGDSDRNATMEDLNAMKYVEAVIKESLRLYPSVPGITRELQTPLQLKNYIIPPMTTIAVYPFILHRSENIYPNAEEFIPERFLDEENKAKFQFGYLPFSAGARNCIGQKYAMNQMKIVVSTILRNAKFESLGSKEDIQISTQLVLRIESLPKMKFFNL |
| ApCYP4c1-7 | MDSSTLILICIAVALIAIAIVRRLEFVASFRSIAGPPSLPIFGNALQLNGSPSDFFRLLLEWHSKFGDTYQLWIGLRPFIAMANADHIQQILKSTVHIDKNLEYNLLLPFIGTGLVTSSGSKWHTRRKLLSPTFHQNILEGFLPLIEKQMKTLVKVLRKEVNNVNGFDIKPYAKLAALDTIGNTAMGCEINSQENSQLDYVKALDELTAIMQKRFITPWLKPNLLFNLTSLSKRQKACIDVIHTFTRKVIKERKDNFKLFNNQTSDANKNEIHYEKKPNRALLDLLIEVSEDGKVLSDEDIQEEVDTFMFAGVDTTSVTLSWVMYVLGKHPHVQDKIVEELNQKIPNFGDGNLTLNILSSLDYLGRTIKEVLRLYPSVPFIGRQIYQPLTIGDHTILPGTSIFINVFALHRNEKHFENPEKFDPDRFLKEKKNDRHRFAFVPFSAGSRNCIGQKFAMIVLKIAVATVIKTYRVKSIDPEEKLGLVGEIVLNALNGIHVTLEERT |
| ApCYP315a1 | MANRYCSLVLVNSTKKRFMSTSNLKTVITESKKEIPIVKGLPLVGTMFSILAAGGGRKLHEYIDKRHQKYGSVFREKLGSVDAIWISNPLDMKLLFAQEGKFPKHILPEAWLLYNDTYGQKRGLYFMNGKEWWKYRQIFNKVMLKDLNVNFIKSYKVVINDLLNEWELSNGQVIPNLIADLYKISISFMVAHLVGRVYDDCKNDLSNDINCLAQCIQKVFQCTVKFTVIPAKTSKLLKLNIWNDFVIAVDNSIESANNLVSKLMSLNGDGLLNSVLNVHDIPIDMIKRLMIDFIIAAGDTTAYSTQWSLYTLGLHKSIQNNLRHSLLKTDFLECDYLNNILKEVLRMYPLAPFITRIPPSDIYLTDHKIPANSLVIMSMFTSSRNGKYFNSPNEFIPDRWNRLKNNKYNGVNEPFATLPYGFGARSCIGQKMAHVQMCLTLSECIKRYNIHTMQPVEIALDLITVPDDQINIKIHKL |
| ApCYP49a1 | MSVLARRLRNLRITVDHANKSTEVFTSVSQGDVDFVKDYSELPGPKSLPLLGNNWRFMSYIGDYKVTEIDKLSLRLWKEYGDIVKIEKLLGRPDMVFLYDADEIEKVFRNEELMPHRPSMPSLNYYKHVLRKDFFGDLAGVIAVHGEKWYEFRTKVQQPMLQPRTAKFYIGTIEDTATAFVNRIKKIKNKDQEVPDDFLNEIHKWSLESIAKVALDQKLGCLEDEHAVDSDTQNLIDAINTFFANVPELELKIPFWKLFSTPTWRKYINALDTITNVTSKHINRSMDRLLSQKSFCPDSQSSLLQRVLSLDPSNPKLAQILSLDMFIVGIDTTSAALASILYQLSRHPDKQKKLREEIRTVLPNADSKLTSSKLEQLQYLKACIKETLRMYPVVIGNGRCMTKETIISGYKIPKGVQVVFQHYAISNSSKYFSQPDQFLPERWLKGSGYKHHPFASLPFGYGKRMCLGRRFADLELQTVVSKIFQNFEVKYEYGDLEYTVHPIYMPDGPLKFKMIED |
| ApCYP301a1 | MYCCRTGDFNDILRKLFTMKNIRQFQIHSIRWRSTATQHAHSPHVSAGSPEALEVTNDLITAKHYSQVPGPTPWPIIGNTWRMLPIIGPYQISDLANVSYILYKQYGKIAKLGNLVGRPDLLFVYDADEIEKVYRQEGDTPFRPSMPCLVKYKSQVRGQFFGRLPGVVGVHGEAWREFRTKVQKPVLQPQTVKKYIQPIEEVSDYFIKRMQEMKNENSEMPADFDNEIHKWALECIGRVALDARLGCLNPDLPKNSEPQKIIDAAKYALRNVALLELKYPFWRYLPSTLWKKYVSNMDYFIEICMKYIDDAMLRLKNKSQSVNESELSLVERILANEPDPKTAYILALDLILVGIDTISMAVCSMLYQIATRPEEQEKIHQEILKILPNKDDKLDASKLEKMVYLKAFIKEVLRMYSTVIGNGRTLQKDMVICGYRIPKGIQLVFPTIVTGNMEEYVTDCKQFKPERWLKQSTDYIHPFASLPYGHGPRMCLGRRFADLEMQVFLAKLIRSHKLEYLHKPLEYKVTFMYAPDGELKFKMTERPTS |
| DcCYP6a13 | MNCEFYNKIKYDLKLPYGGLYHLMTPKLMVTDPELYKYILIKDFDHFVDRGIGESDAKRDPIEGHLFGSKGEYWKRLRNKLSPTFTSGKIKYMFPLIKKCSDQLITTIRKQIGESKQMSLEVKDYCARYTTDVIGSTAFGIEINSLENPDSEFRQVSLLVMKPSVLQMLKAVLSELIPLIKYLNLKIFDPRISQFFSNLVKTNLSYREKNNIKRDDFLNIMMQLQIAQRGKTLSPDDVEMTDDVITAQSFVFFFGGYETSSSVLTFCLYELARNPDIQSKLRQEIMATKKKEGELTYEICHKMSYLDKVTKALRMYPSLPQLDRISVKKYKLPNTDLTLDVGTKISIPTFAIHYDPEYYPDPEKFDPERFSPENIESRPHYTYLPFGDGPRNCIGMRFGTIQFKIGLAQLISNFEFSIDSANATFEYLKGSILLQT |
| DcCYP6j1 | MLEWIRPLLDSFVLLQILLVTTVVLLYKWSTSTMDYWKKKKVAYIPPLPLIGNLGRVITNDTNLGYLLKEMCDKKINERFFGFYMTREPVLILNDAELVMQVMLKDFSQFNDKGFHIDNDFDMILNNLFFLKGRQWKFQRNKMSTGFTSGKLKSMYPTIHKCSTSFINHLTKLSKQTDNLELNIKSAIEDLFTDVVCKCILGLDMHSIENPNREYKDIQDATLKPNWRLHVKQVLQFLSPYIDRKIRITGKHIEDWFYGFAEKNMTFRTENKIQRNDFLQTLINIYSEQHNIDAGSEEYFGLRHLAANIFVFIVAGYETSALTTTFFLQKMALYPEIQERLRQEILDVKKSKEDGAFEYEDFKKMTYLSMVLDETLRMYPVLPIYLRRNIVPYTIPGSDVTLDVGTYVLTPTVALHYSSKYWKDPHKFDPERFSEENKDSIVPGSFIPFNEGPRQCIGKRFAHVQMKSAICEILTHFEIQKTPRTVEHTEFVVGSMVMVPKEEIVADFVPRKFY |
| DcCYP4d2 | MFNMHISALYLLLLATLLFAICLWKRRKLYVLSYKLDGPFALPLIGNALLLLGGPEKAFEAVHTEYYKHRNKGLKIARAWLGPELFILIGDVHMVTTVLNHNLKKAKIYHVLDEVLHRGIFVNPDIPKWKASRKLIVNVFHFSVLKSYIRIFHSEAAILVNKWRHLADGRTSFNPEEDINLATFDMVMMSTLGINPRAQENPSHEFLVNFDSAFQIGMSRMLNPILHSDLVCRLIGLKARQKAHVQVLIDEAQRTLDRIRERQKMNREKENAEIKDPSSHLMDPEAKSFAELIMENADYVESDDEELISQIITIIGAGQDTTKTENMVVLIMLAIHADVQDKVLEEIDRVLGRNTTHCPSYEDLCQLEYLECVIKETLRLFPAAPLIGRHIDEDFILDGLTIPAGVTVLISIYALHRDPKYYPSPGRFDPSRWQTPDAAQRPPNAFMPFSCGPRNCVGGKYAMLQMKTVLSTLLRHYRVLPGAECRSMQDVKFEIRITLKMNEKCAIRLQRRM |
| DcCYP6k1 | MGLLAFNLLVLFLTALVAFYKWSTRNLNYWKIRKIPHIKPAPVVGNYWELLLGKVNVAEKYREFYHQMKDVKYFGILIGTYPALLIKDPGLIMRILVKDFRYFYDRGFHVSDSDVLGTNLFFLRNPKWKLLRNKTVSVFSSAKLKSSFDILDKCSDDLIDYLNENAHKKEWPTRQLMGDAMTDIVCKAICGLEIHSIKNPSREHVAMQNMAFASSFRGFARNTINFFSPYLAKKVHIFPRILHTYFDDLTWKTIEMRKEKNIRRSDLLQLLLEVYEQEQMLPAENRVLSRELFVSNIFIMILAGYETSTTTSSLTLHELAHHPDIQDKVRKEIKQVTRESGGKITYDSLKKMKFLDQVLSETLRIYPLANMIFRECMEEYNIPGTDHVIEKGVFVQLPTLALHTDPALWKDPETYNPDRFSEENSASIIPGSYSPFGDGPRICIGKRIH |
| DcCYP4c62 | MELLHILLTLVVVVLGTKLFYWYRRRLVFIRTIDKIHGPPSWPLLGTVLYDLVPRDKLFKHAMARTQTFKPVYRSWTGPSAIVHLTRPEHIELIMNNSKNIDKGFGYNFLHPWLGTGLLTSNGQKWFSHRKMITPTFHFQILETFMDTFVQKSDILTDKLSQQLACKEGFDIFPFITKCALDIICETAMGSEINAQASSESDYVRAVYDISTLVLERMMRPWLWPSLIFGLTRDGKRHEENLKILHGFTRRVIEERKAARAAGGVREGNDLDENFGNKKRVAFLDLLLEASESSETPLTDEELREEVDTFMFEGHDTTAAGLAWTLFLLGSHPEHQDKVYAELEEIFADDPGRKINSRDCAQMKYLERVIKESLRLFPSVPFIGRVLTEELQVGEYQLPVGCMLNIEIVSLHRCADQYPNPEVFQPDNFLPENVQKRHNYSYIPFSAGPRNCIGQKFALLEEKCVLASILRKFKVISLEKLDDVTIMIDLILRPASGVKVKLEPRHKIN |
| DcCYP4d8 | MSLASLSMIIRAACGVDFKIQQRHHEEHPFITGVEKAFEIFMLRIAKPWLGVGIIFSLFGYKKQLDQACKTTRAFTENIIDKIKTKIVQENSAVEYSIEGQPKIMEWNMFGDLIKGQPKITQDQRKTLDNASLVPLHEVLDLSKETFVEILVRDHLTNTHQEQRITHSELIDEVLTVVSAGYDTTKTTNSLNLIMLALHPKIQQEVYDEIVQVLGDDPGPSRPTIKSKSYTFLLEYQRNVKDYTLPHPS |
| DcCYP6a2 | MKVCFAPGLFQFIRSLLSFFFPKLAVHFTLFPQEVADYFTKLTTTSIEMRKQEKAQAEKRVDYLQLMIELYEKDLMLPESERVIRMQEVVSGVFVLILAGHETSSSTSTNVLHELAYNQEVQDKARREVQKIYKEGGGKVTYEDLAKMTYLEQVISEALRLYPLVNCLFRECTQDYAIPDSRHVIPKGVLVHIPTYALQTDAALWSDPLEFNPDRFAPENESKIVPGSYAPFGDGPRICIGKRFGFLQMKLVLSKVLLNYRVTPCSKSERRYPIKTQTLLAAPEGDHWLKFTKLKPEY |
| DponCYP345E2 | MAAVLILCTILLVLISTLLYKYFTRNFNYWKSKNVFYIKPIPLLGNMLPALIMKTTLGEWLGDLSHKIKKDYFGIFVFDRPVLVVQSPKLVKLILQKDFEYFQNRSVAHYEHDPILSNFMFMAKNPRWKAVRSKLSPVFSTGKLKQMFPHILNEANLMVDYVRNLANVPNVESKEICAKFSTNVIARCAFAIDAGCFNTENAEFRYLGRQLFDFRYSTAFRLFTGFLAPKLASALDISLFDPRVLNRLSSIFGEILSSRISTPELKGKDLIDLFIEESKSKNVHFDQNVMMAQALQFFAAGFETVSGTLSFTLYELCVNLQLQERLRTEIFDNLEKHDGIACEGVSEMKFLDMCIAETLRKYPVLPFLDRTCLRDYKVENSDLVIEKDLDVYIPMFALHYNPEFFPQPQKYDPQRFADKCKINNEGLYYVPFGDGPRICIGNRFGLLAVKIGLVSLLSRYKLEATSQTPRPIKFEPKALILQSNVGVPLKFVPI |
| DponCYP6DE1 | MALIIAVGLLLSIILLYCVYSKWKFQHWTRKGVPQLNPRLFFGDTLPLVRGQALKDFHLGLYQKFKSTGAKCVGIYNAHMPELVPIDIILLKDIMVKDYSYFSSHGVFYHEKNVLTSHLFNIEGQSWKERRTKLTPVFTSAKMKQMFDTVASKSPGLVRLVGLSADANKPADIKEILSRFTTDVISGAAFGLECKSLYEPDNMFRAIGKEAFEPNALKLYIEYLFPRQLLGSIGYQTFSSRIVSYFSKVVNETIQYREKNNVQRNDFMQLMLQLKQHGSLVKEDGSVDVKKTETQITDREILSESFLMFLAGHETSSSTSTFALFALSQNQDIQEKVRNEINEVLERHNGKLGYDALMEMEYLDKVIRETLRKYPTVPVIPRRCTKDYQIKNTNTVIDKGTRLYIPVIGVHLDPEYYPDPERFDPERFSPENKAKRPDIAWMPFGEGPRQCLGMRFGLLQSKVALASLLPTFRFTINKAMKPPYIADAGTLVYMFKQDVLLDATRIN |

**Table S3.** The amino acid sequences of 46 GSTs used in Figure 4

| **Protein name** | **Protein sequence** |
| --- | --- |
| LeGST1 | MSNNSTMGLLIISNPVFECYAFYASILILKMIMMSFLTALQRFRKKVFISPEDTAISKGGGEVRYDDPDVERVRRAHLNDLENIPIFLITGLLLVASKPPEMMANNLFRIYTFVRIIHTICYAMFVLPQPTRAILFIAGAVINIIMVLYVILNMHNI |
| LeGST | MTYKLTYFNFTGLGEPIRFLLSYLDIDFEDIRIDFEKWPSIKHTIPYGKVPLLEIDGKVLNQSLAICRYLAKKANLAGSDDWESLLIDIAVDNFNDFAQNIKSYWFDSNEESKAAKYVTLVNETIPYYMEKFENIIAENNGYFVNGKLSWADLFIVSVLDFLIYKSDIDMLKDRPNLRALREKVLAVPKIKSWVEKHQ |
| AcGSTd1 | MTIDFYYARWSPPCRTVELVASILNVKLNPIVTTPSKGDTQRPEYKQMTPQHTIPTIVDNGFVLSESRAICKYLVEKYGSATGAYSKEQLYPKDLQKRATIDHRIDFDLGALYRRASDYFGPVFATGHYGTAALPKLKAALEILDAYLAKTKWVAGPEVTLADIVIVVTISCLEIVGYDLTSYPNVLRWFKAAQTTLPNYNEANHEGTIEFKEYLFSKLLD |
| AcGSTd2 | MPIDFYYTPGSPPCRSVLLTAKALGLELNLKTLDLHHGEHMKPEFLKINPQHCVPTLVDGDLALWESRAIIVYLVQAYGKDDSLFPKDPKKQALVNQRLQFDVSTLYPAFADQYYPWIFAGVPKSDDKEKKIHDALAFLEIFLGSSAWAAGDSVTVADIALVASISTFEAVDIDLKKYANISKWFEKCKSTLAGYQEFNQKGIDGFKIMVANLTKK |
| AcGSTt1 | MVKLKFYYDFLSQPSRTLYIFMKKTEIPFEPKPLNLRQGQHLTEEIESLNPFKKVPFIEDKGTVLIESVAILRYLCRTYNVADHWYPLDIKKQALVDQYLEWQHNNTRAHCTEYFRHKALWPLKTGQAANVEKVEKLEKKMIENLNLLENIWLKDKPFLCGDEITISDLVAACEVEQPRIAGFDPLVDRPKLTKWITRVQSNFSPFYKEAHHIVVQTADEYQYYASELSKKNS |
| AcGSTt2 | MAKLILYYDLLSQPCRALYMFFKKANVPFEGKVVDLLKGEQFTPEFVALNPFKKVPVINDKGFVLIESIAILRYISRTHNVADHWYPKDSVKQAQVDEYLEWQHTNTRADCGLYFLHKVLWPAMNGKPANEQRIAQLEKKMIITLDLIENVWLKNKKFLCGNEISISDIFGICEIDHTRMAGFDPYANRPNLSEWKMRTSSYLSPYYEEANERLEEHVVKYNKKYGKLNSKI |
| AcGSTs1 | MTSYKVTYFDITALAEPIRFLLSYLNIDFEDFRFQREQWPALKPTMPFGKVPTLEIDGKVLNQSTAITRYLSKKAGLAGSDDWESLLIDIAVDNIHDLRQAIAGYAYDPNEESKEAKYAPLINETIPFYMDKFEKSIEENNGYFVNGKLTWADLFFVAILDYLNFMAKIDLLEGRPNLKALKEKVLEVPQIKAWVAKRPTNNP |
| AcGSTs2 | MTSYKLTYFNLTARAEQIRFLLSYLNIDFEEVRIGFEQWPTIKSTMPFGQVPVLEIDGKVVNQSVAICRYLAKKAGLAGGDDWESLLIDIAVDNIYEIRQEIMKYYHERNEEIKSKLRDTIVNDSIPFYFDRFEKIVSENGGYFVNGKLSWPDLYFVSILDHIKAVINVDLVDGRPHLTALKHKVLDIPQIKSWIAKRPKSLL |
| AcGSTo1 | MSTKHFAGDSVEPPKVPGSYRFYSMRFCPYAQRVQLVLNAKGTPHDTVFIDLLDKPKWYLDIFPAGKVPALIYDGNFLSESLLLADFLDEQFPEPPLWNCSPLQKILDKLVIESFSKVGTAFYKLIFTSETVEEINFNELVASLIPIEAELAKRGSKFFGGDKPNMVDYMIWPWMERLDSIKPYTQGKFVIPFDDKFPKLASWKALMIVDKAVAPYYLTPEKHAQHFVNKKAGLPAYDF |
| ApGSTo1 | MAIKHLSKDSVEPPKVPGSLRFYSMRFCPYAQRVQLILNAKGMPHDTVFIDLSDKPEWYLKIFPAGKVPALIYDDKFLSESLLLADFLDKQYPEPPLQASSPLQTILDKLVIESFGKVGTAFYKLIMTTKEIEKQNFDELVASLIPIETELVERGTKFFGGNKPNMVDYMIWPWFERLDAINPYSNGTFVIPFEDKFPRLAEWKSLMIADKAVAPYYITPEKHAEHFTKRKAGLPAYDI |
| ApGSTt1 | MAKLIYYHNLLSQPSRALYMFFKKAEVPFEGKVVDLLKGEQFTAEFEAINPFKKVPVVNANGFVLIESIAILRYICRTYNVADHWYPKDSVKQAQVDEYLEWQHTNTRADCALYYLHKVLWPVMNGKPVNEQRVAQLEKKMITTLDLIENVWLKNKTFLSGNEISISDIIAICEIDQTRIAGYNPYANRPNLSNWKMRTATYLSPYYEEANEILEMHVAKYNKKYGKLNSHI |
| ApGSTd6 | MTSTLIKYNVILIVLCIGITLIHNSSEATVKKEVNLYYDPFNPQCRSVLLTLGALDLELNLKRIYLFHSEQLPEDFRNINSLHTLPVMQDGDLVLSESNAIIVHLVRKYGGQDDHPLYPNNPKIQAKVNQGLHFNNSYFSQAFEIPHIFRGILKTAEVEDKIHEALNFLEEILEKSTWTAGNTITVADFALVASISTFEVVDFNLGNYQQIQNWLSKCKTTMASYDTANQEGIYELKSLLESKNGFQNKSK |
| ApGSTd7 | MPRIDYNTSSLYNIIRYYNTNPIVLTTTTVPSCRSVLLTIKALNLEVNLKVIDLLADGTQEKDFLDINSLHTLPVMQDGELVLVESHAIIVYLVQVYGKKNDPLYPHDPTFQAQINQRLDFNNFYFYLAFEIQHDDKDDGRIPKIVGMDKIHKALKFLEEILKKSIWTAGNIMTVADFALVASISTFEAFDVDLGKYENIKNWLSLCKSVMPNYDWANQEGIYAVMAIR |
| ApMGST1 | MISFEVDEVLFRTYVFYTAILVLKVLAMAPLTAKQRFAKMVFANPEDAKMNPKSKVKYDDADIERVRRAHLNDLENIPLFIIVCFGYLLTIPNVYIAINLIRLFVASRIIHTIVYAVVVLPQPARGLSWFAGFATTVYMAVQVILSFV |
| ApMGST2 | MSDTSTMGLFMISNPVFECYAFYGSILILKMIMMSFLTALQRFRKKVFISPEDTAISKGGGEIRYDDPDVERVRRAHLNDLENIPIFLITGLLLVASKPQVMIANNLFRIYTFVRIMHTISYAVFVLPQPTRAILFIAGAVINIIMVAYVILSMHYF |
| ApGSTs1 | MTTYKLTYFNLTARAEQIRFLLSYLNVDFEDVRFEREQWPAIKPTMPFGKVPVLEIDGKTFNQSIAICRYLAKKAGLAGDDEWESLLIDVAVDNIYEIRQEIMNYYHEPNEEIKSKLRGPIVNDSIPFYIDRFENIVSENGGYFVNGKLSWPDLYFVSILDHIKSVIDVDLVDGRPHLTALKHKVLAIPQIKSWIAKRPKSQ |
| ApGSTs2 | MAVYKLTYFNFPALAEPIRFLLSYLEIDFEDVRFEREQWPSIKPTMPFGKVPILEIDGKVLNQSAAISRYLSKKAGLAGSDEWESLLIDIAVDNVNDLRQAIALQVFDSNEESKAEKYVTLINETIPLYMNKFENTVVENDGYFVNGKLSLADIHFVAIIDFLSFLAKVDLLEGRPNLQAHKNKIFDIPQIKSWIAKRPAFSMKL |
| DcGSTe1 | MNPLKKVPVLNDNGIYISDSHAILTYLTSQYGMNSSHLYPRDLKKRAIVDSRLHFDNGVLFPSLANIIRPMVYEGQTTILEDKKKIALEALDFVEGLLKQTEWVAGDKMTVADFSLVATVTSLATLLPEVESYWKIQAWIKRCEKNMIFYEEVQRPGLESFKSLANRNAAPK |
| DcGSTe2 | MGLILHEIIASPPVRAVKLCLTELGLEAEYKTCNLLAREQFSDEYLKLNPQHTVPTLEDGDLIVWDSHAINAYLVSAYGKNDALYPKDPKVRALVDQRLHFDSGVLFSALRNIGLKIFFKNEKEIPEEDKLRAREALDFAEKFLQGRKFITGDTYNIADFSIYTTASALVALVPGLEKYPNLAKYFDLCKSSFKGISHDEEGLQAFKGLVQQKLSGK |
| DcGSTd1 | MTIDFYYVPGSAPCRAVQLAAAQIGVPLNLKHTDLMKGEHLTPEFLKLNPQHTVPTMDDNGYTLSESRAIIAYLAEQYGKDDSLYPKDPKARGIVNQRLYFDIGTLYQRFADYFYPHCFGGAPLDAEKAEKLDQALGFLNTFLASSPWVAGDNITIADCSIVASLSTIEIVGFDLSKHSNVVAYLAKAKTSLKDYEEANNKGTLAFKGLVDHLTKK |
| NlGSTe1 | MTIDFYYMDISPPVRAVNLCLAALNLEVNRKEINLFNRENLKPAFIQLNPQHTIPNIADDGFVLWDSHAINAYLVSKYAKDDSLYPKDIQKRAIIDQRLHFEGSVLFTHGVRCFLPLFFGLSKTIPEDQRSTDQYYEMVDKFLEGNTWIAGDQMTIADFSYISTLSGLSQIFTGVEKYKNISTYMDRCKENMKDYDSANQQGVDKYVGILKNILQSE |
| NlGSTs1 | MSGYKLTYFPVTALGEPIRWMMSYLDIKFEDYRFEREQWPSIKPTTPFGQVPVLEIDGKSVWQSVAISRYFGKKADLAGKDEWESLMIDVIVDTFTDFRLAVGKWFYESDEATKKKLEKPLLETTVPFYLEKFDSTIKENGGFLANGKLSWGDIYFVATSGYINHMLGFNMSDKYENIKALCEKVAAIPKIKEWIDKRPAGI |
| NlGSTt1 | MSGRQSAVTLYYHLLSQPSRALKIFMDVNKIKYIPKEVQLAQGEHLQPEFEAINPFKKVPVISHNNFILTESVAILRYLCREFDVADHWYPKDTLQQAKVDEFLEWQHIELRAPLAMYFRTKFLMPMVTGKPAKQESVDRMYKLMVIGCDKVENIWLKDKPYLCGNSISLADILGACEIEQPRMAGYDPAEGRPKIKEWMDRIKKDLDPHYADAHAYLNAVAKKNAARSSQVVSKL |
| NlGSTz1 | MSIIGKPVLYSYWRSSCSWRVRIALNLKEIPYDIKPVSLVKGGGEQHCNEFREINPMEQVPALQIDGHTLIESLNIMHYLEETRPQRALMPQDVHKRAKVREICEVIATGVQPLQNLTVLIYVGEEKKKEWAKHWITRGLRAVEKLLSSCAGKYCVGDELTFADCCLVPQVFNARRFHVDLRPFPITLRIDRELENHPAFRAAHPSSQPDCPPEATK |
| NlMGST1 | MSSNLYTSDNPVFSAYLFYCAILVLKVLLMAPLTGRHRFAKRIFANPEDKLPKSVVKYDDPDIERVRRAHLNDLENIPVFMVAALLYIATNPSYWLALTVFRVFTVARIVHTIVYAVVVVPQPARAIAWGVGYAATIYLAVQVILFSL |
| NlMGST2 | MSSSLYTTDNPVFSAYLFYCAILVLKVLFMAPLTARYRFAKRIFANPEDTTLTPKSKVKYDDLDIERVRRAHLNDLENIPVFMVAALLYIATNPSYFLAVNLFRIFTIARIIHTFVYAIVVIPQPARALAWGVGYAATIYLALQVVLFSL |
| AgGSTd1 | MDFYYLPGSAPCRAVQMTAAAVGVELNLKLTDLMKGEHMKPEFLKLNPQHCIPTLVDNGFALWESRAIQIYLAEKYGKDDKLYPKDPQKRAVVNQRLYFDMGTLYQRFADYHYPQIFAKQPANPENEKKMKDAVGFLNTFLEGQEYAAGNDLTIADLSLAATIATYEVAGFDFAPYPNVAAWFARCKANAPGYALNQAGADEFKAKFLS |
| AgGSTu1 | MKLYAVSDGPPSLAVRMALEALNIPYEHVSVDYGKAEHLTAEYEKMNPQKEIPVLDDDGFFLSESNAILQYLCEKYAPTSDLYPNDPKDRALVNHRLCFNLAFLYPQISAYVMAPIFFDYERTAIGLKKLHLALAAFETYLQRTGTRYAAGSGLTIADFPLVSSVMCLEAIGFGLGERYPKVQAWYDGFKQAHPSLWAIAAKGMEEIAEFEKNPPDLTGMVHPIHPIRKPAAK |
| AgGSTu2 | MPAPTLYYFPMSPPARAVLLLMKELELPMNLKEVNPLAGETRTEEFMRMNPEHTIPTLDDNGFYLGESRAILSYLIDAYRPGHTLYPNIPKEKALINRVLHHDLGSFYPKFFGTIGALFSGAATEISDEMKTTTQKALTDLEHYLTRNDYFAGENLTIADLSLVPTIASAVHCGLDLTNYPRLNAWYESCRVLKGFEDDQEAARQVGEYLRSKFPTGLEALN |
| AgGSTo1 | MSNGKHLAKGSSPPSLPDDGKLRLYSMRFCPYAQRVHLMLDAKKIPYHAIYINLSEKPEWYLEKNPLGKVPALEIPGKEGVTLYESLVLSDYIEEAYSAQQRKLYPADPFSKAQDRILIERFAGSVIGPYYRILFAADGIPPGAITEFGAGLDIFEKELKARGTPYFGGDKPGMIDYMIWPWCERVDLLKFALGDKYELDKERFGKLLQWRELMEKDDAVKQSFISTEDHTKFLQSRKNGENNYDILA |
| AgGSTs1 | FNVKALGEPLRFLLSYGNLPFDDVRITREEWPALKPTMPMGQMPVLEVDGKKVHQSVAMSRYLANQVGLAGADDWENLMIDTVVDTVNDFRLKIAVVSYEPDDEIKEKKLVTLNNEVIPFYLEKLDDIARDNNGYLANSKLSWADIYFTAILDYLNYMTKSDLVANHPNLQRVVDNVTSIESIRSWIDKRPKTEI |
| AgGSTt1 | MSKNLKYYYDLMSQPSRALWIFLEKTKLPYEKCLINLGKGEHLTEEFKAINRFQKVPCITDSQIKLAESVAIFRYLCREYQVPDHWYPADSRRQALVDEYLEWQHHNTRATCAIYFQYVWLRPRMFGTKVDPKQAEKYRGQMEGTLDFIEREYLGSGARFIAGDEITVADLLAACEIEQPRMAGYDPCEGRPNLTQWMARVRESTNPYYDQAHKLVNKFAQDTASKAKL |
| AgGSTz1 | MANVDILPESQPILYSYWRSSCSWRVRIALNLKEIPYDIKPISLIKSGGEQHCNEYREVNPMEQVPALQIDGHTLIESVSIMYYLEETRPQRPLMPQDVLKRAKVREICEVIASGVQPLQNLIVLIHVGEEKKKEWAQHWITRGFRAIEKLLSTSAGKFCVGDEITLADCCLVPQVFNARRFHVDLRPYPIILRIDRELEGHPAFRAAHPSNQPDCPPEAAK |
| GmolGSTD1 | MPDLYYVPGSAPCRAVLLAAKALNVTLNLKLVDLHKGEHLKPEYLKINPQHTVPTLVDDGLALAESRAIITYLANKYGKGSTLYPEEPRARALVDQRLYFDIGTLYQRFGDYFYPQVFGGAPADQEKKQKVEDALKLLDQFLDGQKYVAGPNLTLADLSLIAGVSSFEASDIDFKKYSNVNRWYETVKSSAPGYQEANEKGLEAFKSLVKK |
| SzeaGSTd1 | MGNCIENEGIAPLNNGVINLKMSGSNEPIDIYYFPPSPPSRAALMLIKALGLKHNVKTVVLPNGEHMKPEFLKLNPLHTIPVIEDNGFTLYESHVIMKYLVDVYGKDDSLFPKDPKKGAIVNLRCYFSACYLFPRFAEYHVPTMFHGVPPSEEGLKKLKEVLEHLNGFLDNQMYVAGPTLTVADFANITVITTIDASGSVDLRSYPNIWMWYQRCKKSMEEYGFEEVNQAGASAFGQMYKTKLSSIK |
| PiGSTd1 | MYSIRSLAFMFLVVLGNAAARSKSSRMPAQAIKLYYLPPSPPCRAVMMTARALGLDLNLVLTNIMEGAHMTPEYIKMNPQHTVPTMDDNGFILWESRAIMAYFVNAYGRDDSLYPKNPRLRAVVDQRLNFDIGTLFPRYIDVYMPVLFKGEEMSQEAVDKLNEALGWLNTIMEGKTFVAGDNLTIADISIVITFTNLDAFDYDFSEYENLTQWFERTKKALEPYGYMEIDHAGGQILASFLKKD |
| GST-msolf1 | MPSEPIKFYYLAPSPPCRAVMMAARALDLELDLIPTNIMDSDHKTPEYLKMNPQHTIPTMDDSGFILWESRAILAYLVNAYGKDDSLYPKNPRQRAIVDQRLNFDIGTLFPRYSNLYFPMLFRGDEYNQENADKLNEALGWLNIFLEKSAFVAGDNLTIADISIIVTITNLDAFKFDFSEHENLTKWFERTKKALEPYDWEDIDETGAQMLADFLKREH |
| BmGSTe1 | MVLTLYKMDASPPVRAVYMVIEALSIPNVKYVDVDLLAEDHLKEEFLKLNPQHTIPMLTDDKFVIWDSHAIATYLLNKYGKGSSYYPEDPEKRALIEMRLHFDSGILYPALRENDEPIFFWGETTFKPEGLAKIKSAYDFTEKFLSDSPWIAGDDVTVADMSCVATIGSLDALLPINEKEYPKITSWLKRCSELDFYQRGNNVKGLLEFKALLKQYLSRGKE |
| BmGSTe3 | MSLMLYKLNASPPARTAMMVCELFKVPVKMVDVNLSKGEHFSPEYLKRNPLHTVPTLEDGDLIITDSHAIAMYLADKYGKDDSLYPKDLKSRAIVNQRLFFDSTVLFSRMRSVTFPVIIEGCKTVTEKQINDIIEAYGYVETYLSNTKFIATNNLTIADISAYAVVSSLLFIVPLDGAKFPKTQTWLNEMEKKPFAQKYNVNGVAELGALLKEKLGS |
| BmGSTe4 | MVFILYKKDTSPPCRSVQMVLHELGIYDVELIEVNLPERDHLKEEFLRMNPQHTVPTLIDGDFIIWDSHAIVTYLVNRYAKNDTLYPKEPKQRAIVDQRLHFDTGVLFAILRATAEPVLYNNEKSFKQENLEKMEAAYEFVEKFLTSDWLAGDQVTLADICCVSTISSMNVIVPIDKKKYPKIISWLQRCSEQEFYKKANEPGLKKFIEMFKNKIGN |
| BmGSTo1 | MSEKHLQTGDVLPPYSGKLRVFAMRFCPYAERTVLTLNAKNIPYDLVFINLDQKPEWIFNFSPKGTVPALEYEPGKALFDSNIINVYLDEKYPEIPLQASDPLRRAQDKILVESFAPAQSAYYTAAFNAQALEPSMVETYHKGLEGLQKELETRSTKYLHGDEPGWVDYTLWPFLERFEALPLIGKAEFAIDQTKYERLVTYIEAMKNVPAVKSYFLAAETHAKFIESRAQGDANYNMLDTSAVCCMRPRKKKE |
| BmGSTo2 | MSAIKDSRNINFNIKHLRKGDPLPPFNGKLRVYNMRYCPYAQRTILALNAKQIDYEVVNIDLIDKPEWLTTKSAFAKVPAIEIAEDVTIYESLVTVEYLDEVYPKRPLLPQDPLKKALDKIIVEASAPIQSLFIKILKFSDTVNEEHVAAYHKALDFIQEQLKNRGTVFLDGSEPGYADYMIWPWFERLRAFAHDERVRLEPSKYSLLLEYIDNMLKDSAVSQYLIPLEILAKFHEAYTKKERPNYELLNECLKSF |
| BmGSTs1 | MPKVVYHYFACKALGESGRMLLAYGGQDFEDHRVLSADWPDFKPKTPFGQTPVLVIDGKQYAQSTAICRYLGRKYGLAGANDEEAFEIDQNVEFLHDIRAKAAAVYYEADEELKAKKHEDFSKNVYPDMLKKLNSIVEANKGHIAAGKLTWGDFVFTSMFDYLKTMLQIPDLEVQYPAFKKVLQSVLTQPKVKAFLDLGRPYEFEF |
| BmGSTt1 | MVLKLYYDLMSQPSRVLYILLKTMKYDFEPKYVNLRKAEHYSEDFTKVNRMQRVPVIDHNGFILTESIAILKYLSRENVIAESLYSKESKLQARIEEFLEWQHIGLRLHCAMYFRVVHMDPILTGRKSDEKTIQGYKRRMMMALDDFDTKWLGRGTAFIVGETPTVADLVAACELEQPRMAGFEPKDHFPNIAAWWPKVRDHFAPHYEDAHVILNKIINKMDRAANSKL |
| BmGSTz1 | MGKQPVLYSYWRSSCSWRVRIALNLKEIPYDIKAVSLIKGGGEQHCNEYREVNPMEQVPSLCIDGHTLIESLNIMHYLEETRPQRPLMPQDCFKRAKVREICEMIASGIQPLQNLIVLIYVGEEKKKEWSQHWITRGFRAIEKLLSTTAGKYCVGDEITLADCCLVPQVFNARRFHVDLRPFPIILRIDRELENHPAFRAAHPSSQPDCPPEVAK |
| BmGSTu1 | MVLKLYAVSDGPPSLSVRQALVALEVPFELINVDFGAGEHMTSDYALMNPQKEIPVLDDEGFYLSESNAILQYICDKYRPGSPLYPQDPKSRAIVNHRLCFNLSSYYANISAYTMAPIFFDYERTPLGLKKVHISLDVLETYLTRTNTSYAAANHLTIADFPLINSTMTLEAIDFDFSKYTKIHKWYNDFKVKYPDLWKISESAMKEIQHFAANPPDLTHLNHPIHPIRKIKN |

**Table S4.** The amino acid sequences of 61 UGTs used in Figure 5

| **Protein name** | **Protein sequence** |
| --- | --- |
| LeUGT2B7 | MATTKMPTAMIASLVLWLAVWTMPLADTARILAVETVAGKSHWYFMSSVLKSLTEAGHTVTVFTPFPDGDRANYTEVDTSRDFPIKLDMDVMKTIQDFSEPFKLIDIMSKMVRFYCDAVYGNRKLADLMASGIRDRYDLLIIEPLGLDCVSYLGDALGLPVIYSIPSPMITFSERLFTGHLSNPATVSNMLANHAVPGTFVQRFANTVLLTYSLVRIRYDQLVTWFTDPRPYDLAPTVNPSIIFQNSHYITESSRPVTPNVVYVGGIHLKPAKTIPKDILDFIEGSPQGVIFLTFGSTIKVSSFPEHIEKSFKEALANIPQRVLWKYEGEMKDKPKNVMTRKWFPQREILLHPKVKLFISHGGMSGVYEAVDGGVPVLGIPVFYDQPRNIEHLVLAGMAISMDLLSTTREKLSNAISELINDEKYAKNAKIASNRFKDRPMTPQQTVVYWTEYVIRHKGAPHLRSHALNLTWYQYLLLDVMAVVLIFVFLVTFVVFKVFKCIKAFRNDKVKTQ |
| LeUGT2B13 | MSNVCRRRLRRNDSAAAATAMCRAVVTVVCCLSLVAGPWTTPVDGMRVLAVETIAGKSHWNFMRAVLRALTDAGHTVTVFTPFLDGDRDNYTEVDMSAAFRMKLDVDLAEVLDKFVEPSSILPLIVTISRSLCDAIFEDDRMKDAMRPPADRGDGGYDVVIVEPIGSECVSHAATVLGLPLVFVVPSPMISHYEGSFLGHVPNPAVVSHMMADHAVPRTFVQRFRNAVLLAYSVFTVRYTEWSLKRADGSKPYDTLAPVRPSAVFVNSHYATEASRPVPPNFVHVGGLHLEKPNRLPTDILKFIDESSNGVIYFTFGSVVKMSTMPDYIQKSFKEALAQVPQRVLWKYEGQMDDIPPNVMIKKWFPQRDILLHPKVKLFISHGGISGVYETVDAGVPVLGFPLFYDQHRNIANLVDAGMAISMELLSVSTDMVLNSILELINNEKYSINAKITSERFKDRPLSPEKLIVYWTEYIHRHNGAPHLKSQALTLTWYQYFLLDVIAAILILILLISFVCYTIIKSIYKYFLKYLHNSKRKSEKLS |
| LeUGT1-7 | MKYIMILVILLNFYFRAIKSSNILVFVPSPWKSHITSFQPLFLELANRGHNITVISKFIVKDPPPTYTQLIPSYEFDIDGRSDFLMRERSIHYSFFEDPITRSTILVDTTYMFLSDPEVQKFIRYDQSTFDLVIIESFFQECTVALGHKYRAPVISIVPVTPWVSVSRWTSNPTDFSYIKDFMLDGGKSMTFWERFTNSYIGFYCQFVELITYLPKLENMMDTYFQYPGYENRPTMSEMLKNISLSLIDSDSTIFSPRPYIPSFIEVSGIHIKPKKQMDERLQDFMDKAKTGVVYFNFGTILNVTSIPKLSLRSLINVLGRLEQKIVFRWINNDTRKFPTNFYVNSWLPQREILNHPNCKIFITHGGVHGIIETIDAGIPIIGFPVFGDQFQNVRISQENGIGIMSNIFTMTEETFEKDIKLIINDKKFSENVKRMSSIFHDRSMSAMDTAVYWVEYVIRNKGAHHLRSIAVDLTWYQYYLLDVITFFIIVLLCFICICYFVTKRIMRFMFNLFTKQKMD |
| LeUGT2C1.1 | MFAAVQRPLLLLLLPMVLSPLPAALAANILAFMPMPIKSHFGGFQPMFEELARRGHNVTVVSSFPMVNRSVPNYTDVDVTPVKGLPEINVMDLIDLNFLTSVINRWFFAHLLSAQMEHATLIDFLRSEDNSFDLVMVESFLQEYTVAIGHKYNAPVINLSPSMVWVSASKWLHLPSTFSYVPDCCIGVTDDMSFTERLKNTIVGLVEMFVEDYLYIPMIKATMSKHFAYVGWESRPTLEQMLNNVSLTLMNAYHVVGVCRPYLQGVIEVGGMHIKQPKPLPKDLQNYIDSATNGVIYFSFGSIMNLSNLPKEKLSIFLNVISRLKEKVIMKWVPDKSIKLPQNVKVGSWLPQNDILAHPNVKLFITHGGLHSIEEAVYNAKPVIGIPFFADQHSNLRLVEKIGYGKLITFGQLTEESLGNTIEEVISNPAFKDKAIIQSRVYRDQPMNPLDRAIYWIEYVIRNDGAQYLKAGSIGLNTAQYFLFDVTLFLLLLIAIFAWLGYRGIVKVSSKYKAD |
| LeUGT2C1.2 | MRWLPSSLQSVVTLAAVLVCATDAARILAVETMAGVSHWNFMSGVLRALVDNGHTVTAFTPFPTADGDRPQENYTEVDLSRELQAARDNQMADIVQKWRTPVGQTLQWARRSRYVCDKVYDHPAMRDLLRLHHRDGRPPFDIVIVEPFLSDCVSYVAGQLGVPLIFASALPAIGLMERLHTGHAPNPATASHLVASHGVPKTFAQRLSNSALFAYSNVAVEYAERVLKYIEPREYDTAPPVKTSLMFINGHHVSEHPNPVLPSIIHVGGIHLKTPKKLPKDILEFIEQSSHGVIFFTFGSTVKMSSIPEHIRNAFIDVLAQIPQRVLWKYEDELKNKPKNVMMKKWLPQRDILMHPKVKLFISHGGISGLYEAVDAGVPVLGFPLFGDQYRNIDNLVNAGMAISMELSSVSEETLLKHVLELINNKKYRINAKTASNVFKDRPMTPAQSVVYWTEYVLRHKGAPHLKSHAINLTWYQYYLLDVISFTIILIYVVIFAIYKILKCIYNCLSKCYHNVKAKIE |
| DcUGT362B1 | MYNMKHVLWILTVICSLFLENSCAFRILVLCPHISRSHFTIFESIAKGLTDHGHVVDILSHFPQSSKIPNYNDISVEGSMKLQTNDLLLSDISYYNPLSDFFFIHQMGEDTCEAVMSTKAALDLLHSSKKYDLIITEVFNTDCFLGFVYKFKVPYIAVSAAHIIPTAAERFGIPDNPSYIPNAFLSYDSDMNFVQRMFNAITTLSINLMRKYYYDPKHHKVATKYFGEDLPPLDQLARNTSLVLVNSHFTFMGSRPYPNNVIEVAGLHVKSPQPLPNDIKKFLDESVNGVIYFSMGSIIQGKSFPSDKRKAFLRAFEQIPQRVIWKWEGENMSGKIDKILLKSWAPQRDILDHPNVKVFISHGGFLGTTEALYSGVPIIGIPMFGDQKANIRVVEKAGFGVTLPYDQITEETVLVALRTVLGNPSYKKRAEKVARLFQDRPMPPLDTAIYWIEHVIRHGGGAHLRPASLELYWWQYILLDVIIALILLIAAMVWSIQWLVRYALITYYNTVDDKKRN |
| DcUGT374A1 | MTTNPEKMESHENLTTVDWSFTYNVWKKSESLDLQTRKSQWTNVRFFASKFSTTCTSAQWAHSNTQRFIKEIVSANKTYDLLIIECWAHQSYLGLVDLIGNPPVIMTATAHSFAPLTMPFNNPENPAYVPVLWTANTDTMTFWQRVTNTVQYHMYNWYFMKTMQEEDEVRHANLDKKYIKRSLREIFMDGSKNSFMGSFDSRITGHVRPVQPKIVEVGPLHLVDPKPLDESLQKWMDGAPEGVIYFSLGTNMKGTSMGDFRRKAFLKAFARLPQYRVLWKWETDDVMEGQSENVRLQKWMPQQDVLAHPKVKLFIMQGGLQSLQEAFHYGVKLICIPMFGDQDLNAQRIAKIKTGVVLEFDNLNEDNIYDALITVLEDPIYEENMQYYSSLSKDTPMSPIEKSIWWIEYVLRHKGVAHLHSAAADLSYVQYYLLDVIAFIITILLVLLLALYLATVRVLRKLNVIRLESEKDIKKKIK |
| DcUGT374A2 | MVTVKRCGYWTLFAVTCFISSAIASDILVVCPTASYSHQQPFLIISQALLQRNHTVTLLTCNPEKIKPHVNLTIVDLSFTYELWKNKKDLGLQGRYSQWKNLYTFSTVFSVLVTSEQWKHPNLRNFIDMIVTTNKTYDLMLFERVLYHSYLSLVDLVGQPPVIGMATAQYFSIISWPFNNPENPAYVPVIWTANTDSMTFWQRVTNTVQAMAYYWLHMRTLDKEQAILNQNLDEKYRKRSLREIYFDSAKDSFMFSFDSRITGYARPMQRKLVEVGPLHLVDPKPLDESLQKWMDGAPEGVIYFSLGTNMKGTSMGDFRRKAFLKAFARLPQYRVLWKWENDVMEGLGENIRLQKWMPQQDILAHPKVKLFIMQGGLQSLQEAFHYGVKLICIPMFADQDLNCQRVGKIKTGIVLEYEDLNEEIIFNALKLVLEDPQYEENMQYYSLLSKDTPMSPIEKSIWWIEYVLRHKGVAHLHSAAADLSYVQYYLLDVIAFIITILLVLVAAIYLLTKKVLDLVKSKLANPKEKERTRKKLK |
| DcUGT375A1 | MWSSTLLLSLCLLTHGEAGRILVLMPYPIWSHYQQFEPLFEALASRGHHVTVYSAYPPSTNLSNFHHVNVDIVEAREFFAGLNHFQLKESGDYGDFTFSSHLQSTVQAFGYGIKMASEIFNNTHIAALIASDHKFDLVISEIWFAQEALAVFGHKFQAPIIGLISYGTPHTVSTYMGTPNLYSYIPDYKFAFPARMNFLQRLQNTILGVYTQLVGDWWYYPKLDGIMRDFANHSAELPHLTTLLRNVSTTFVYSDVMLEYPRPQTSNLIHVGGIHLRNKKLPKDLQDLMDSATRGVIYVSFGSLIRPSRMSDSMRTLLVTAFSRTGLTVLWRYEGDSIENLPGNVHIRKWIPQQDVLAHPNCRLFISHGGVNSALEAIHYGIPIIGVPFYGDQLSHVRHIVDLGAGVELSYFNITLESIAWATSIVLNNPRYKASALAASRRFKDRPDSSLNTAIWWVEYVLRHHGAPHLRSAYDDLSWAEFLLLDVLAFVSGVVFLVLYILLRMGRMVKRLLSPSVGNKGKSKPKSKRS |
| DcUGT375B1 | MAKSIPSIFYLLLLTSHLQAARILIVLPLPLWSHYMQFHPLWQSLAERGHNVTVYSAFPPETPIPNFRHVDLTLPKIKDVFDSYDHFERKAIQASQSNFEQGIYTQNLLFNFGIFISQEVFANRAFQDLLHSNDTYDLVILESFFGQEALAVLGHKFQAPIIAETSYGTPHNCFLFMGNPNLYAYMPDYKFAFPARMDFLQRLQNTAMGLYAHVVGDWWYFPKVDEIMRTFTKPEDNLPYIKTMMNNISATFIYSHPILESERPQVSNLIHVGGMHLKNEKLPKDLEDIMSNAPQGVVYVSFGSIIQPAKMPAELKDTLVRVFSKMRQTVLWRWKGTPIANLPKNIVQKNWVPQQAVLAHPNCRLFISHGGVNSVLEAIHHAVPLVGVDFYGDQKSNVQRMVDLGAAVPLSYFNITEKSLLWAANTVLDNESYKLAALKASRRFNDRQNSPLNTAIWWVEYVLRHHGAPHLRSAFDDLSWVEFLLLDVLAFVTVVLLTVLYVLLWIARAVKCRLYFAKKAKSE |
| DcUGT376A1 | MAASWKLFLGLIVLGLYSTSINSYRILGFFPFPIYSHQVPFFRVFDELITRGHDVTLVAGFPLSDTDPKLYKYIPIPFLLKFNDAKGRSPLLFAHKSTAKTVNHYSNFCLTSLQNILTTREVTEFYRTDNGTYDLIISEITFCGEPHAALGHKYRGVPVINFQPLGYRAAVFSLYGNLLMPNIMPDVREPFTDRMTFWQSLDNMYLTLWDLWDMYMSYLPQLEEMMRANLHYPGSESRPDLVSLLHSMSLTLVEYDPAILGVAFPIAPNLKFTGGLHLRPGELLSADLESFMNGPGAKGVIFISLGTILQFSHLEPHVTAAFVEALKSLPYAVIWKVDVPVEMPSRVLTGQWFAQQDILGHRNCKLFITHGGIHSSMEAVYHGVPVVMMPGFSDQHQNCKLMEEKGMGLITPHETINGDILYITIREVLNNPRYRDTVGRLSKQVRSLPYSNLDQAVRWAEHVAANKGVLGYTPAAQQTSVMRLLGVELWVIWTILVVLVVVIVMSLIRCLRRCCRKK |
| DcUGT377A1 | MLLPSSLLLILLGIGAAQGANILIIAPLATHSHAIWFDIVTTLLVKDGHQVTVLSSDPEKQSLPNRTTYTLETSYDTHEYEMDLGKMKTEWSSSVAGVYKSLLDWNIENCRRQKGSRGLAEFLEKFVRKTEKVDLIIREGAGSECYVALIHLLGYPPVIAATPFPNFEVPGYWIGNYDNPSYVPYSLTSYTDQMSLLQRLHNAYIGLYFKFMRSFYYLNQIDELSKEIFGDGVPHVKSLDDHVVLAFVNHHPVLDYPRPTVPAFIPVPGLQLKPPKKLPQDIQSFLDGAKDGAIIFTFGSSLLTASISPVYRTMFFQVFSQLKQRVIWKWEMEHPADKPDNVMLAKWLPQADILGHNNTIAFISHCGQAGTQEAIYHAVPVLAIPFLLDQSIMAAKLIAKGVAIELNYEELTFDSVKSALDTLTTPTHGYKERMQKLSKLFRDTPEQSSDKIIFWTNYILNHGGKHLTPASKQLSGIQYYIIDVAACVVLTPIALVYLTRALAWLVRRQAKS |
| DcUGT378A1 | MNHFPFIVLLSFSFLGTLSCDKILVFLPLPIWSHQMQYEKVWTALAERGHEVTIYTKFTPKSNSTNLKHVPIRLPKIDARMKEFNPAILTSSVNEILAMLFLWDFGYEGQVEAFQSPEFKKLMASKDQYDLVITESFFAMESSVALGHRFNAPNIVICSFGIAMNSLNFFGSPNLPSFMADFRTKYTNEMTFFQRFYNFYVFFVTRIMEYVYYYPKHQALVDTYFGKDYPSLYTMLSNVSLSFMYTNLAMSAAVPLVPNLIPVGGIHLNKPGKLPQDLQQRADAAKGGFVYMSFGSVVDPTKLSEETKLGFLEVFKQLKLPIFWKIDITNDPVLNAKTLPDNVFIQKWYPQTDILAHPNLRLFITHGGISSLMEASSLGVPVLGVPFFGDQYRNMVLLRHRGYALIEPIQTLTKQSFLKNAQTMLNDPSFKQNAKKWASIANDEIVSPLERVVYWTEYVLRHKGAPHLSASSRQLTWYQMYCIDIILVILGILYAVAKLLSMCCCRSSKKHTQVSSTKKKN |
| DcUGT379A1 | MKTHQGRNQYLISAAILLMCIRIQQCSKILAIFPTPAKSHQIIFDTILVELYQRGHDLTVITQYPETLVHYERMKVLDIKGTHTYNSTIEDIYELSADSIKRIHINFIDQEIQVENIFLHENMKSIWNMENKYDLIITEMFLTDAFLVIPYLYKVPYISIASSTLHPQHSERLGLPDNPSYIPSYVSAYTDHMSFTERLSNTFVGLYYKWYYDYKSHGAANRIIHKYFPEIPRIQELLNTCSLTLVNTHHTINIARPLPANVVEIGGIHVKPAKKLNEEMERFLNESHHGVIYFSMGSMLKTSSFPPDKFKAFLKAFSKIPQRVLWKFEDNDTSIFKPYKNIRTSSWMPQRDIFAHPNMKLFISHGGLLGITEAVYEGIPVLGIPVFGDQWANIKKLESLKAGKLLPYLEITEETVSDALKTVLSPEYKENAEDLGKRFRDRPQSPLEVAIYWIEYVIKYNGAYHLQSAAVKLTWYQYYLLDIALVLIVGLVSVCFVLKYLCGSLVRRKHKTE |
| DcUGT380A1 | MIRLTLIFLGVLLCHIDASNILAFFPMALNSHIKPFQPLLYELSRRGHNVTEVSSFPPPPGVDNYTYVYVPHLFNATNTMEFRNRTHADLIGLFHSLCLAQMEQVLRTPEIQTFVQRDDSHFDLVIIEGTFCGECLLAMGHKYKAPVINFQPLGYWPSNYYVYGNLLSPAVIPDFRLPSTTQMNFWGRLDSLWFAVTDLFLTNLFYYPKQVALMDKYFKYPGYQSRPPMVDMLRNISMTFVEHDISIGVPQALTPNMLFTGGMHIKHAKPLPPDLDKYMSDAPNGVIFFSFGTNVRFANMPPYVLNAFVESFSKIKQKILWKTDVEVEVPPNVLVRKWFPQADILGHKNCRLFLTHGGIHSAMEAGYHGVPVVMMPGFSDQFQNVLLMQEKGLGRVIDMDSLDSDVVVEAVNAVLGDKTYAANAKRISAIMKSSPVSSLEKAVYWTEYVIRHEGAHFLKPASTRLSLVQFLCLDILLVVISVMAAMLFVLFKCGQVLLRAKKKDKTE |
| DcUGT381A1 | MKYTRLKYQQSAMAMASAVNYLLSLLSLCWLVSPTVDSANILALFPTASYSHQMPLLALPRALAQRGHNVTVITTNPLKEPMPNYTEIDISFTYNYWKSTAGEKRLINLDTKIGPIEVVNSFISGTNILTDIQLGSPQVQKFITYLDKEKPKFDLVLYEDLMHTSYLGFLHKLDYPPLVSMLTLPLLCGLDLSSGNVCQPSYISEMIIGRTNKMSFYYRLEGYLYLLYARLVLAPRIFSAQDALAKKYFGSSCPSTKDMVRNRSLLLSSSMWIFEYTRPVFPNTIHVGPLHIGDTKPLPQDLAKWVEGGKKGAIYFSLGSNVKSAALEDSKRTAILAALARFPDYRIIWKWENEELEGLPSNVICRKWLPQHDLLAHPNIKLFITQGGLQSLQEAVHFEVPVIGIPFFGDQNYNVKIIRRLGIGSYMEFEDIHTENLFENIQEILNNYDRYKKAVKRASDISKTQMMSPRDTAVWWVEYLLKADGNVSHLQPEYWHLTWYEYFGLDVYLVIFSPVILALYGLYRLVLTINRRWSKGKLKSE |
| DcUGT381A2 | MRMSQSIWVIIAIAVTSADSANILAIFPSASYSHQIPLLAVTRSLAQRGHNLTVITPNPSKVPIPNHTDIDISFSYSFWHTDDSKEALLSLQRRIDAFELLDQFTSITCTAVEFQLNSTAVQNFIKYIDKEKPKFDLVLYEDIPYPAFLGFLPKIGHPPLVSMLTLSVPCAFDFSFGNVCNPSYVPEVWMGFSDKMTFFERLQNYLFVFVTHYWIKTRVINGQTELARKYFGHTGKPSLEEMTRNRTLLLITSSWLYQFPRPVFPNTIHVGPTHIGDTKPLPEDLRTWIEGAEKGVIYFSLGSNMRSASLEESKRSAILTTFAKFPQYRVIWKWEEEQLPGLPSNVICRKWLPQHDLLAHPKIKLFITQGGLQSLQESVYFEVPLIGIPFFGDQDYNVKIIKNLGIGTYMDFDSVSTEVLYNLMKEILYNTSYMDTVKRISALSKTQMMSPRDTAVWWIEYVLKSGGNLRHLQPDHWDMPWYQYFGLDVFLVLLSPVILVLYGIYKIISRCKRKSSGEKLKKS |
| DcUGT381B1 | MFDLQKPEMLASHSQLALILMAFLLTVESANVLIICPTPSYSHQVPFIAIGKELVRRGHTVTMIGTDPLKEPPVNYTDIDLSFSYKYFKPQLQKGEVLPDAVDNQRRLTGYEFIVNIGRITIAYTEDQLKSQQMQQFFKYIDENHVKFDLIIYEGLLHTAYLGFLPKLGYPPLIVMQTINPTCLFGLHSESMICNPSYLPEIMSGYTQSMTLMERMNNLFMQLYSKFYIRSRLMKKQDEIMERYFGTRGLSGKQLEENKTLLFISTSWLLTYPRPVFPNTILLGPIHLNNPKPLPQNLKDWIEGAKDGVIYFSLGTNMQSASLQEDKRKAIVDSFKQFPRHRIIWKWEEDILPDLPSNVMCRKWLPQHDILAHPKVKLFIMQGGLQSSQEAIHFGVPMIGIPFFADQDTNVRKLESMDVARFLEYENITAETLVTLMKSILYNETVYRKSQVYSKLSNTQMMSPKDTAVWWIEYVLKAEGNVDHLKYNLDQIPWYQYYLVDLVGIFIAGIFLVVFVLFRMALLLRRLCFRSKAKKKKQ |
| DcUGT382A1 | MLLSPYAWLLPLLCLIGPAYSVNILAIFPHHGLSHFMNVKPYLLELARRGHNLTVISFFPLKAEERPAGGNYHDVSLDGCMQLYNNNVSFARFEDKGFAASLLSPTQLNLAGLYMMADETLAAIFKAPELSRFIRSVLTEGHNFDLAIIETFQMDVFLGLIHRMGVPFISVTTCNLFSWSADRIGQPQNPSYVPTNIDLFGDHMTLIERIKNTISLLTMSVVHPLFFLANDQRMAEAQFGPGLPPLAEIAKNTSLLLANVHFSLNRPRPVVPQMIEVGGLHIAPAKPLPKDLAQFLDASTQGVIYFCMGSLLRGESFPKEKLQAFLSAFSQLPLNVLWKYEGAELPGKPSNVKILPWTPQRDILSHPNIKLFINHGGLLGTTEAVYEGVPLLGIPMFGDQPINMKAVELAGAGVILDYASLSQQTILEGIRTVLKPEYKFNMRLLQAQYRDRPMSAMDTAIFWTEYVIRHKGAPHLRTAAVHMPWYQTLCLDVALVFITVLLAVVFCIKLSITLVLRKLCSSNSSTHKNKKD |
| DcUGT383A1 | MSPVLVTLLAILLVHQVHPYNILVTNLIPFQSHLMTTSVIANHLTERGHNVTLITLKATRPHPKLNVILNKDVFRLPPNFPNIVGNFSAFEFGKFMMDRATDTSDELLSTPLFRKLINVGTADFQDSDGIPVYNISYDVIIAENHFMQEIYGAALSEKFACPLITYQPILTPPHAAHLLGNYYNPAFMADYKLRYTGNMTFWQRLINSLLTAYEILYQNFIYLPRIDTIMRTHFAKVGAAKWPYIKDILRARNALTLVDTHHLVTDPKPNNPNVIEIGGIHITPGKPLPQDIEDFINASPAGVIYFAMGTFVDGENLTPKRKANLLKLFSGLKQWIIWKIDPSNFQETLPPNVNVGKWFPQNDILAHPKCILFITHGGIHSVLESLYHGVPMVGIPVFADQAQNLLALQEKGMGEMVEFNFEYEDLKRKLDKVLNENSYRQKIAKFSAIYRSEVTDIVERTMFYIEYVVRHNGAHHLRTASTRLTWYQYLNLDVLLVVGLGLGGSAYVLYAVVFWLLMALRKSGKNGASRSKKTN |
| DcUGT383B1 | MKLAVAFSLVALLTSPRGCHGYNILVLGVFPVPSHVMMLSVISNELVAQGHNVTFVTTKATRPHPNLNVIINEKVMHFEPAFLEEVGRAGATGLATILWNLGLDGTDKVLATDVLQKLINAQDASQPKFDAILTETYFLTETIAAGFAQKFDCPLINYHPVVMTANVAYMVGNPYNPGYMPDYKMPFTSNMNLWQRIQNTYVALYLTLYQNLIYFPAQDKLLRKHFAPLGAAQWSYIGDILRQRQALTLVDTSHVLTDSIAHAPNVMDVGGLHIKPAQPLPKDIEDFISSSGEAGVIYFAMGSTISTENLGPVRIETLCKLFGSLEQRVLWKFDSPELEKSSKLPPNVKLSKWFPQNDILAHPKCRLFITHGGVHSAFESIYHAVPMVIVPLFADQKQNGQKAEEEGYGLMVDFDVFDYEELRRKVHQVLYEPKYKTNVQRLSTIFRSEPLHPLQKAIRSIEYVIAHRGAPHLKTKATSLPWYQTSQLDVIASCTLGLGLVLYTLVRIGTSLAEFTRGKDDKKATKPDRNKKNK |
| DcUGT384A1 | MFRIWFSLALTALLCTTSQSARILVFIPLFNPSHYMQYEPLFEALAARGHHITLYSPHTLGQNLTNFKHVYLKNQAFSESAKIFNPLKKEETYVIFRINPKVPSSTKVNREIISEPHFNNLIRSGRGSFDIVLAEPLFGQEATVYLGHALGVPLINLGASAAHADILDVMGSPNIVSHMPEFYSSLTDRMNFIERAINFIYAIHRYYLRLWTYWQVDHMIAAQFGANVLPSVESLLRNISLSFVCTDVGLEYPRAQSGNIVPIGGIHIERNGNLSLPEDIQKTLDSASRGFILYSLGSIMKSEAAPDTLARTLVETFSKFDNYKIIWIWNGQQVTELPSHVVQIKQWVPQIPILAHPNCKLFITHGGLKSQIEAVHFGVPMVIIPYFYDQFQNAAKAVEFGLGIELSNKNLTVESLEWAIRSVLHDSRYKEQAMARSRILKDRLRSPLDTAVYWTEYVLQHEGALHLSPVSRHLYWFQYYLLDVMACILAVGVVVVLLMRRMVRNLLGKRVNVGKEETKMGIGKND |
| MpUGT329A6 | MESQLKVLMFVHGACTSVIIIVTVFSPANTANILAVFPHQGFSHNVVYQPYILELANRGHNITIITNYPLEHPNINNISIRGSMPISNNKKNISHIEGKFMNEIQSSISTIWNFHYRGKIYEAMFTVDGVKKLLNSPSKFDLLITEHFNNELFLGFAFKFNLPFILLSSCNMLPWNQHAIGQPYSLANIPSTLTGLGTKMNFYNRAMNTISHIVQLLGYKLFRRTRDEAIIKQKLDIEVSLDQLILNASLIMVNTHFTMLESKPLVPAAVEVGGIHITPIKPLPVDIQKYIDEAENGVIYFCMGSLLLGESFSAEKRQMFLNVFDKIPQRILWKWEGELPGKPSNVMIQKWMPQRDILAHPNVKLFISHGGLLGTTEAVHEGVPILSMPMFGDQVTNIKAIVRRGAAEMMNYGDLNEDEIFVKITSMLTNPIYKQKAKELSEAFRDRPMPALETAVYWTEYVIRYKGAPLLRSATVGTPWYQYYLIDVLFVIFLFVTTIVVLLYCLIFKVLLRLLNRKSKEKQS |
| MpUGT329A7 | MMIVLFVIIIITATVVSPADTANILAVFPHQGLSHHLVFLPYVQGLANKGHFITLISNYPIEHPNINNLSIRGSIAITNNKENISHFESDSMNEIQRSMNTIWSFYTRGTMYEAIFTVDSVKTLLNSPSKFDLLITEHFNNELFLGFVLKFNIPFILLSSCNLLPWNQQAIGQPYSLANIPTTLTSLSAKMNFNSRVINIISHAVQLFGFNLLCRTRDEAVIKRNIDFEISVNQLILNASLIMVNTHFTMFESRPLVPAVVEIGGIHIMPTKPLPIDIQKYIDEAVNGVIYFCMGSLLRGESFSAEKRRMFLNVFDKIPQRILWKWEGELPEKPSNVMIRKWMPQRDILAHPNVKLFISHGGLLGTTEAVYEGVPILSMPISGDQVTNIKSIVSKGAAEMMNYGDLKEDDIFIKITTMLTNPIYKKKAKELSEIFRDRPMSPLETAVYWTEYVIRHKGAPHHRSVAVGMPWYQYYLIDVLVVIFLSVTTIFVLLYCLIFKVILKLLNRKSKKKQN |
| MpUGT330A5 | MKGQRDTVITATALLLVTAVIRCAAGYDILAIFPFSGKSHFHMFRAVSEALTARGHHLTVVGHFPKSPKEPSRQHQSQIGDTNGGGDGGGSYTDYSLVGSMPVYENFTTDEVTGNGYLKEMLIILQDGLDNCEAVLSSGRLSQLIQSRAKFDLVLVEIFNTGCFVSIANHFGAPVVGLTSTSLYPWFVGMVGDVVMPSYVPVNLLPFTSRMMFAERLINSVILVAMKTYYKFKYEHAAQEMVDKYLGKLNGGTVSESLNNVNAIIVNTHFVFGDTRPLPPGIIEVGGCTYKKPMPLPEDLERYVTEAERGVIYFSMGSIVKSSSIPTNQKMAMLRVFGRLDGYRVLWKWEDDPPPPEVRPENVMFVPWMPQFDVLNHPNVKLFISHGGLMGILDALYSGVPIVGIPLFADQFSNMNFIVQNECGLQLQLDLIDEQVAGDTISTVLHDDKYAKNAKRFSTLYRDRDQDPLEKAMYWVEYVARHRVDLMLKPATQDWWYERCLLDVAVAVTVAVATTAYLAKRVLAKSRIAVATDHR |
| MpUGT341A7 | MFYQVSIIITILTISGSGVVRPIAAVADILAFFPLPIYSHFSGFNPLFLELANRGHRVTVVSPFYPKGDVLTTYRHVPIPDVKIQRTRSPMEIRHRYRVNNMINVRLGMMKMVTRTLELNETRAFLNDTHNAFDLVLTECWYSDIYLAIGYRYSAPVVCLSPMAPSVTLSQSLGMPDHPAYVPSFWLRYSDSMSFGERLYNSAIAAAELIVSEIAFQSTDQQMLDDLYTYPGHRNCPPLDALRQAVQLTLVNGHHSVSYARPYPPNVVQVAGMHIRPQTSASIDRKFKALLDGATHGAIYFSFGSNIKMSDLGERDVQSFVESFRKLKQIVLWKWENGTIANLPDNVYIDKWFPQQYILSHRNCKLFITHGGYHSLVEALHYGLPLIGFPFYTDQYYNMRFVVENSFGIEILLENLNVKVLVDAIGKILSDTSYKENAHTASNIFLDLPVSAMDTAVHSIEYLIRNGVADYKLPASMSLNRYQYFLVDMAVSIGAILALTTLILYKSIEYLRKII |
| MpUGT341A8 | MFSQVSIILVILTIFGSGVVRPIAAVADILAFFPLPIYSHFSGFNPLFLELANRGHRVTVVSPFYPKGNVPNTYRHVPIPDVKIQRIRSPLEIRHTYRVTNMINVKQGMIKMVTRTLELNETRAFLNDTRYAFDVVLAECWYSDIYLAIGHRYSAPVVCLSPMAPSVTLSQSLGMPDHPAYVPSFWLRYSDSMSFGERLYNSAIAAAELIVSEIAFQSTDQQMLDDLYTYPGHRNCPPLDALRQAVQLTLVNGHHSVSYARPYPPNVVQVAGMHMRPQTSSTVDQKFKALLDGATNGAIYFSFGSNIKMSDLGERDVQSFVESFRKLKQIVLWKWENGTIANLPDNVYIDKWFPQQYILSHRNCKLFITHGGYHSLVEALHYGLPLIGFPFYTDQFYNMRFVIENGFGIEILLENLNVKVLVDAIGKIFSDINYKKNAQTASSIFLDLPNSAMDTAVHSVEYLIRNGVADYKLPASMSLNRYEYFLVDVAVSIGVVVSLTVLILYKSIYYLRNITNLKDKKSK |
| MpUGT342C4 | MDTKCLLWILLLLATTNTINAAKILAITPIAAASHWNVMSSVLEVLLNRGHTITVVSPFPRKIPHENYTQIDVSKLVPFAIASPWETVVGVYKPPTRCLKFLNDVQQHMCRTAFNHPDLQLALQTQHYDLVITELLASRCDLYLASSLGVPHVAIMSSQMLTWYQDSFDSPSNPSYITTLNSPYPKPETFVQRFWNVVDYVTIFLYFKHIDNAATEMGRQYFGDGRPHAEALLRNVSMVFLNTHSNFDLSKPLATNFKEIGGIHLKPPKPLPTDLQEFIDSSEHGVIYFSLGSVVRMEDLPIAIQHGLTEGFGELSQNILWKLESDRPIINLPKNVITRKWFPQYDIIRHPNVKLFITHGGNSGVIEATSAGIPVLGFPIFFDQPRNLELFKHWGSGLFVNYDNFTKEDFVCKIKRILSDQKFKDNAVDLSHRFHDRPLNPKDTVAYWTEYVLRHDGAHHLKSQAVNTKWHQYFSLDFLVITSVIIISLLYFFYNVISIVLK |
| MpUGT343A10 | MDLVSMNFVTSVQLKWRICIQLSEMVMSHRKMKQFVQSSSNSFDLIMVETFGQEYAVAMGHKFNAPVINLAPAMIWASISKWLHVPSTFSYIPDACTQSTSDMGFIERLKNSITGFMQLYVENYLYLPKTKEVMDTYLKYRGWESRPPLEHMLNNISLTLVNSHNAIGISRAYLPGIIEVGGMHIKDPKPLPKNLQTFLDTANEGVIFFSFGTLVNLNDLPKAKLKVFISVLGRLKQKVIIKWIPDDNNVKLSQNIMTGSWFPQRDILAHPNVILFITHGGLHSLEETVCNAKPIVGIPFFAEQNFNMKIVEEKGYGKLVNFFEITEESFGNAIEEVLSNVTFKEKAIIQSLVYKDQPMKPLDRAVYWVEYVIRYGGAGHLKSDSIGLNDLQYFLFDISLIVLLSFGLIAWLCYFLFVKIMS |
| MpUGT343A11 | MQRATCSTRERHLVTTSKKSKIFLPLIVLFYTNIINNNTRLKLLHFCLFKYKMLPSMFLCTIVCAAIAVAKASNILVFMPMPFRSHFRGFQPLFEELSRRGHNVTVVTSYPENRQIANYTEIGPFISKGHERNVTEMMHKNFVTSALSVWKLGEILTEVLKHKSMVDFLQTNSDSFDLVIIESCCQEYTVVLGHKFNAPVINLVPAMLWSSISKWIHVPSTFSYIPNILLETTSDMSFTQRLKNTITGVLQLYVENYLYLPRMKEVMDTHFTYKGWESRPSLEDMLNNVSLTLVNAHHAVGVSRPYLPGVIDVGGMHIKESKSLSGDLQTFVDSAEHGVVYFSFGSVINLNHLPKEKLNIFLGTIEKLKQKVILKWTPNASVKLPQNVMSGSWFPQSDILAHPNVRLFITHGGLHSLEETVYHAKPVVAIPFFGDQHLNMKLVERKGYGKLVDFFKITEESFGNAINEVLSNPKFKKNAEIQSRVYRDQPMKPLDRAVYWVEYVIRNGGAGHLQGDSVGLNDMQYFLFDIVFLLLIPILCIIWLSYLFIAKITSKLY |
| MpUGT344A17 | MFSFAATLVALYACSSSLQWTPASAANILAVQTIPGKSHWNVMRSMLRALTDRGHTLTVFTPFVDGDRDGYTEVDVSEKIAPNLGINATFLIENYIPLRKAMPSMINHSRKRCDMIYGHPRMVDILNRAAAWHFDLVVTEPFMSECVAYVANVLGVPMVHVVAFPVVTYSERPLTGHVPNLAAAGHAMSRRGTPKTFAERFGNVALMVYCSALTWYTEWKLRRSNPRPYDAMDLVRPSMIFINSHSITIPARSQTSDFVQIGGIHLTPPEPIPKVIYRTRSVATLGGLGIVTPKYYTEDILEFIDDAPHGVIFLSFGSMILMSSLPETVQLAFKDALARVPQKVLWKYEEKMKEIPDNVMSRKWFPQRDILLHTNVKLFISHGGISGVYESLDAAVPVLGFPIYHDQHRNIENLVNAGMAIGMDLLSVTEGTLLNAISEIVNNDRYQKNAKIASERFKDRPMSPAESVVYWTEYVLRHKGAPHLKSHALNLTWYQYFLVNVISTFLFIAFVVLFIIYYGLRVIRSHICTFFHSVNTKSK |
| MpUGT344A18 | MPFGDVYIEVDVSGLRSFKPERRKNMFSFAATLVALYACSSSLQWTPASAANILAVQTIPGKSHWNVMRSMLRALTDRGHTLTVFTPFVDGDRDGYTEVDVSEKIAPNLGINATFLIENYIPLRKAMPSMINHSRKRCDMIYGHPRMVDILNRAAAWHFDLVVTEPFMSECVAYVANVLGVPMVHVVAFPVVTYSERPLTGHVPNLAAAGHAMSRRGTPKTFAERFGNVALMVYCSALTWYTEWKLRRSNPRPYDAMDLVRPSMIFINSHSITIPARSQTSDFVQIGGIHLTPPEPIPKDILEFIDDAPHGVIFLSFGSMILMSSLPETVQLAFKDALARVPQKVLWKYEEKMKEIPDNVMSRKWFPQRDILLHTNVKLFISHGGISGVYESLDAAVPVLGFPIYHDQHRNIENLVNAGMAIGMDLLSVTEGTLLNAISEIVNNDRYQKNAKIASERFKDRPMSPAESVVYWTEYVLRHKGAPHLKSHALNLTWYQYFLVNVISTFLFIAFVVLFIIYYGLRVIRSHICTFFHSVNTKSK |
| MpUGT348A4 | MRSAVFAAFVFCALTIAPTGSSEILVIFPTTAQSHYRVVRPLIHRLLDRGHKILAITNFPDAVERANLSQIDIAGLKPHSKFKTSNNGIMKSITRVIGNANTYATILSHPTVAKLLQSGRKFDLVLAEFFTSTPIFAPIATVVDAPIVGFCPMITFPWINDVMGMDTTMSYMPNILSDSSDRMSFFQRISNIVKTAIIYIAFNWIYTPIIRQINNHHYGIQTESAIKSMANLSMIMTNNYHSMFLPFPQLPGIVEVGGIHVVDEKPIPQDLNDFINNADRGVILFSLGTVVSEESLSADKLYNILDAFSKVKQRVIMKFDSEKYKIQLPVNVKMVKWFPQRDLLAHPKVLLFISHAGMMSVIETIHCGKPMVAIPIFGDQMFNTNLLVGKQVAVAIEYKHLDSDHLFNAINEALAEKYTINMKKLQQLYNDRPQSPLETAVYWTEYVIRNKNKSKELLKSQKVHLNLYQSNLIDIFAAFLLPLIVVIYFIQWKMKHILYNGQ |
| MpUGT350B3 | MNSIVLIIGIVISVNSCLSSNILAFLPSKARSHYGAFEPLLKELAEKGHNVTVLSPFSMKNPPSSYHHIQVEDEDLVMSFNPFSVAKHFKPILVPFRSWIMWPKFTEIILNKPSVQKLIHSEGLHFDLVLFENFYHECFVALGHKFNAPVVQLFPSIPNAGVAQWHRNPYDGSYIPDINSGFCDDMSFIERLTNTVLSFIHTALSSFFYFPKQRDLMDKYFNYTGWETRPSMENMLKNISLTLINTHFSVGTPRPLVPSYIDVAGMHLKPASSLPEDLLDIMNNAPEGVVFFSFGSVLKLTQLPKNEFDIFIKLLGTIKQKVLFKWDSDTITDFPPNIIVRKWFPQADVLGHHNCVLFITHGGIHSTEEAIYFGVPMLAISVFGDQLHNSLVMQNRGAAIRIKYSELSEDVLEKALHKMLNDKSFKQKATELSQTFRDQPLQSLNKAMYWIEYVIRHDGAHHLKTAAGELTWYEFLLIDGLFLVVIMRIIITVILWYVGKKLWRRFCCVKSKPN |
| MpUGT350B4 | MNSIVLIIGIVISVNSCLSSNILAFLPSKARSHYGAFEPLLKELAEKGHNVTVLSPFSMKNPPSSYHHIQVEDEDLVMSFNPFSVAKHFKPILVPFRSWIMWPKFTEIILNKPSVQKLIHSEGLHFDLVLFENFYHECFVALGHKFNAPVVQLFPSIPNAGVAQWHRNPYDGSYIPDINSGFCDDMSFIERLTNTVLSFIHTALSSFFYFPKQRDLMDKYFNYTGWETRPSMENMLKNISLTLINTHFSVGTPRPLVPSYIDVAGMHLKPASSLPEDLLDIMNNAPEGVVFFSFGSVLKLTQLPKNEFDIFIKLLGTIKQKVLFKWDSDTITDFPPNIIVRKWFPQADVLGHHNCVLFITHGGIHSTEEAIYFGVPMLAISVFGDQLHNSLVMQNRGAAIRIKYSELSEDVLEKALHKMLNDKSFKQKATELSQTFRDQPLQSLNKAMYWIEYVIRHDGAHHLKTAAGELTWYEFLLIDGLFLVVIMRIIITVILWYVGKKLWRRFCCVKSKPN |
| MpUGT330A3 | MKGQRDTVITATALLLVTAVIRCAAGYDILAIFPFSGKSHFHMFRAVSEALTARGHHLTVVGHFPKSPKEPSRQHQSQIGDTNGGGDGGGSYTDYSLVGSMPVYENFTTDEVTGNGYLKEMLIILQDGLDNCEAVLSSGRLSQLIQSRAKFDLVLVEIFNTGCFVSIANHFGAPVVGLTSTSLYPWFVGMVGDVVMPSYVPVNLLPFTSRMMFAERLINSVILVAMKTYYKFKYEQAAQEMVDKYLGKLNGGTVSESLNNVNAIIVNTHFVFGDTRPLPPGIIEVGGCTYKKPMPLPEDLERYVTEAERGVIYFSMGSIVKSSSIPTNQKMAMLRVFGRLDGYRVLWKWEDDPPPPEVRPENVMFVPWMPQFDVLNHPNVKLFISHGGLMGILDALYSGVPIVGIPLFADQFSNMNFIVQNECGLQLQLDLIDEQVAGDTISTVLHDDKYAKNAKRFSTLYRDRDQDPLEKAMYWVEYVARHRVDLMLKPATQDWWYERCLLDVAVAVTVAVATTAYLAKRVLAKSRIAVATDHR |
| MpUGT339A2 | MTAVVVEPTGRLAVGLQAAMVVVMVLLLEWPSAASGARILAVFPYNGHSHFAMVEPLMVALSERGHHITVISPFPRRKGSGGGRRYVDVDVSDTLPPVISQLNVTKEFGHLVDPVTGFRHLCQMNHRVCEATFEHPLVRALIHEPLRFDVVFAEAFATDCFAAFAHAYDAPLISIRTSDYSPQLNRRVANPQNPAYLVNHLLTYTSRGMSFAQRLVNALATHFGVVGYHAFSDGPSTELVRRHFGPGTPPVPEIARRRTALVLVNAHHSLTQPRPTVPNAVEVGGLHIAQPAEIENEWSDYCDLCDQGVIYVSFGSLLKGSSFPLQFTMAFVRAFEALPYCVLWKYEGEMISKRIKVSKWMPQQQILGHKNVKVFITHGGLMGVMEAVYFAVPMIGIPVFGDQQSNVANCVAKGIAIGLNHRQITVEKLIKSIQAVVMDSKYKSKAFELSARFRDRPSSALETAVFWTEYVIRHGNETNTASLAIDFDFYQYFLLDVVLVGLSILLFLFYTAFRIKKCIR |
| MpUGT342C3 | MDTKCLLWILLLLATTNTINAAKILAITPIAAASHWNVMSSVLEVLLNRGHTITVVSPFPRKIPHENYTQIDVSKLVPFAIASPWETVVGVYKPPTRCLKFLNDVQQHMCRTAFNHPDLQLALQTQHYDLVITELLASRCDLYLASSLGVPHVAIMSSQMLTWYQDSFDSPSNPSYITTLNSPYPKPETFVQRFWNVVDYVTIFLYFKHIDNAATEMGRQYFGDGRPHAEALLRNVSMVFLNTHSNFDLSKPLATNFKEIGGIHLKPPKPLPTDLQEFIDSSEHGVIYFSLGSVVRMEDLPIAIQHGLTEGFGELSQNVLWKLESDRPIINLPKNVITRKWFPQYDIIRHPNVKLFITHGGNSGVIEATSAGIPVLGFPIFFDQPRNLELFKHWGSGLFVNYDNFTKEDFVCKIKRILSDQKFKDNAVDLSHRFHDRPLNPKDTVAYWTEYVLRHDGAHHLKSQAVNTKWHQYFSLDFLVITSVIIISLLYFFYNVISIVLK |
| MpUGT345A3 | MCSGAIRFIIVLCSIGCLSTIPAHRILACEPSAGHSHWNVMSAVLESLVAAGHEVVCLTIHPATDRLAAHPNYTHVDMSSMVTGSLQKARDIDYAHVMKVFRSNAFMVGLATSRAVYVCKYLFDMPEIRDILDGRSPAFDAVIMESLHSECMSTLPDRLGLPVVYVVPSPTVNWMPAATGAPDHPSYLGAMLADRPTPVTFGHRLANALVYAHTTLVRWYNDVGRDHRWPDHRHSMVFVNTHHSIEPSRPVGPNVLEIGGIHLNQPLKPIPRDLADVIDKSNEFGVIVFTFGSLISMNTLPNDVLDAFKIVFSQLPQTVIWKYENDHMPDKPENVMLCKWLPQRAILQHPNVKLFISHGGMSGVYEVVDAGVPVLGMPLFYDQPRNIQNLVDLGIALSMNINNLTHTTLSEAINRLIKDKSFSENAKRVSSLFRDRPMTPSASVVYWVEYLIRHRKEDNIRPSSADASWTSHFMLDIGAALVVAILILWSVTRAALRVVLNKSIPSNVK |
| MpUGT348A3 | MRSAVFAAFVFCALTIAPTGSSEILVIFPTTAQSHYRVVRPLIHRLLDRGHKILAITNFPDAVERANLSQIDIAGLKPHSKFKTSNNGIMKSITRVIGNANTYATILSHPTVAKLLQSGRKFDLVLAEFFTSTPIFAPIATVVDAPIVGFCPMITFPWINDVMGMDTTMSYMPNILSDSSDRMSFFQRISNIVKTAIIYIAFNWIYTPIIRQINNHHYGIQTESAIKSMANLSMIMTNNYHSMFLPFPQLPGIVEVGGIHVVDEKPIPQDLNDFINNADRGVILFSLGTVVSEESLSADKLYNILDAFSKVKQRVIMKFDSEKYKIQLPVNVKMVKWFPQRDLLAHPKVLLFISHAGMMSVIETIHCGKPMVAIPIFGDQMFNTNLLVGKQVAVAIEYKHLDSDHLFNAINEALAEKYAINMKKLQQLYNDRPQSPLETAVYWTEYVIRNKNKSKELLKSQKVHLNLYQSNLIDIFAAFLLPLIVVIYFIQWKMKHILYNGQ |
| MpUGT349A3 | MKYVGLLLVAVLGALSTTDSANILGVFPINGRSHWVVYESLMKALAARGHNVTVITSFPQKTPLANYTDIDVSASFPSAVNTLSVEMVLKYLTSVFANQWFIADHQMNICRQSQKLPEVKALLQSGIKFDAVFTEIFGADCDVGYAYHFKAPLLSIMSSSHLPWSYDRVGGPDNPSYIPTIVTRAAGKMNFKERMINTLYYIYFKIAWKYYSEWPANELLKENFGPDVPHINEIVYNTSMVFVNGHFSLDGPRPLVPNMVEIGGIHVKSPKPIPKDILKFIEDSPNGVMFFTFGSLIRISTLPPNVLQMFKEVFAKLPIRVLWKYEEEMPDKPDNVYISKWMPQRDILNHPKVRLFMTHGGLLGTIEAVHSSVPVIGIPFFFDQPRNILKLVEQGSGILLDYETLTKDILYNAITTIVNNSSYAINASKLAKRFKDRPLNATETAVYWTEYVIRHKGAKHLRTAAVGMPWWKYFLVDVIGFILLVIFGVLYLIYFVLKTIYKKLCKKTVPKKKEKKN |
| AgUGT329A3 | MVNATVTYYLLVAATVAASYRASAARILGVFPHHGYSHHMVFLPYLRTLADRGHDVHVISNFDSSHPNITDINVVGSMPMSNNNVTFPITAGYFGIVGSLTALFGLSYLARTTEGLFDVPAVQNLLEDRTATFDLVIAEHFNSELPLGFAAKYRAPFVLLSSCPLPPWTMSLVGQPLQIAYRPSMLSGLPERMDLGQRLINTAVTAVSVAVFRLINRSWSQQTLRKRMDLDVSLDELASNVSLVLVNTHWSLNGVSPTVAAVKETGGMHIMPPKQLPIDIQKYIDEAENGVIYFCMGSLLRGETFSPEKRQMFLNVFNKIPQRVLWKWEGELPGKPSNVMIRKWMPQRDILAHPNVKLFISHGGLLGTTEAVYEGVPILSMPIFGDQMTNIKAVVSKGAAEMMNYGDLNEDEIFKKITSMITDPKYRQKAKELSEAFRDRPMSPLETAVYWTEYVIRHKGAPLLRSAAVGMPWYQYYLIDVLVVILLTVTTIFVLLYCLIFKVLLRLLNRKSKQKKS |
| AgUGT329B3 | MCLNIINMKMINYSSQIVIQLLVILPFFQDAFCANILGVFPIEALSHHIIFDSYMSELHRRGHNVTVYSHYPDLASEQYKRIQISNVSSVLDPSYVTMDHMYSPSISNNYKHMFHIVSNGELYTQSDALRQLYDQPEDVYDLIVTETCNTDLYLALIERFKAPFIAWTTSPLFVWSADRMGASTHPAYIPVLMTKYGPQMNLVERTYNTLLRLIAFHKYYTGSSISSQKIASKRYKNASQLNQLVLRTSLLFVNTYHALWGSRPLPQNVVEVGGLHVKPSKPLEEDIQKYID |
| AgUGT330A2 | MPIYENFTTDEVMGNGYLEEMLLILQDGLDNCESVLSSGKLDQLIQTRAKFDLVLVEIFNTGCFVSIANHFGAPVVGITSTSLYPWFSGMVGDVVMPSYVPVNLLPFTSRMMFAERLINSVILIAMKTYYQFKYEPAAQEIVNKYLGKLNGGTVSESLNNVNAIIMNTHFMFGDSRPLPPGIIEVGGCTYKKPMPLPPELERYVAEAQRGVIYFSMGSIVKGSSIPTTQFLAMLRVFGRLDGYRVLWKWEDNPPPPEIRPENVMFVPWMPQFDILNHPNVKLFISHGGLMGILDALYSGVPIVGIPMFADQFSNMNFIVQNDCGLQMQLDQIDDEIANYTISSILLDDKYTKNAKRLSALYRDRDRNPLEKAVYWVEYVARHRVNLMLKPSTQDWWYERCLLDVVFAVFFTVALTAYLVKWVLFKSRIATTTDQ |
| AgUGT341A4 | MFYQVSIVMGIFIISTSDVARSITAGANILAFFPLPIYSHFSGFNPLFLELANRGHRVTVVSPFYPKGDVPNTYQHVPIPNIKIQRNTKQNLIDIRHAYRVSNMINVQRSMMKTVKRTLMLNETRTFLNNTRNSFDVVLVECWYSDIYLALGHRYSAPTVCLSPMTPPVTLSQSLGIPDHPAYVPSFWLRYSDFMSFSERLYNAAIAVAELIVSKVVFHYENQKMLVDLYTYPGHQNCPPLDELRRTVQLTLVNGHYSVSYPRPYPPNVVQVAGMHMRSKTSTFVDKKFKALLDGAVHGVIYFSFGSNLKMSDLAERDVQVFVESFRKLKQIVLWKWENGTIANLPDNVYIDKWFPQQYILSHKNCKLFITHGGYHSLVEALHIGLPLIGFPFYTDQYYNMRFVIENGFGIEISLDHLNLKVLDDALGNILNNISYKNNAQRASNIFLDLPVSAMDAAVYSVEYLIRNGVNHTSPVSTSLSWYQYFVIDIMVFISVIIALTTLVLYKSIKYLKKIINLKDKKL |
| AgUGT342A2 | MNASWCIVTIAVGVLTLGRYCHHGADGARILAMMPIAAKSHWNVVDSVLQTLVARGHHVTAITPFPKKSRVANYTEVDMSGLMPSGMSVPWDTVMGECSVHNNLPFLSGRHKDMCRTVYEHDEFWRIITTTKFDLFITELLASSCDAYVSYYLKIPQIVIVSSHVHTWYHHTFGSHMNPAHVSTYHASYAVPTNFIQRMMNTYDYLYSHMVFKWVDRESTVIGRKYFGPDAPDADTLMKNTSLVFVNGHYTVDLAKPLLPNFVNIGGIHLVKPKPLPEDIEQYINDSPNGVIFFTLGSVIRLETAPAYLQTTFVEALREIPQRVLWKYDVPNIEDLPTNVKIGKWFPQRDILEHKNVKLFISHGGMSGIYEAIDSGIPVLGIPLFFDQSHNIANIAHWGAGIMLDHKTLTKDIFLNAINEIMTNYDKYKLKAMELSRRFKDRQNTPKEEVIYWTEYVIKHKGAHHLKTAALKLSWYQYLLIDIIITIVLIVLVSLSVIIILVKAIKNRICNLSKPKKE |
| AgUGT342C2 | MDTKCLLWILLGLVSTDTINAARILAITPIAGASHWNVMSSVLEVLLNRGHSITVATPFLRKIPHENYTEIDLSKRIPLAIGSPWETVVGVYKPPVTCLKFLNDAQLTTCRATFDHPDLQRALHAGHYDLVITELLGSRCDLYLASHLGLPHVAIISSQMLTWYQDSFNSPSNPSYISTLNSPYPKPETFLQRFWNFVDYAIICTYFKYVDTAATVMGRKYFGDSRPNAEALLRNVSMVFLNTHSNFDLSKPLATNFKEIGGIHLKPPKPLPSDLQEFIDNSEHGVIYFSLGSVVRMEDLPISIQYGLKEGFRELPQKVLWKLESDRPIIDLPKNVITRKWFPQYDIIRHPNVKLFITHGGNSGVIEATSAGIPVLGFPIFFDQPRNLELFQHWGSGLFIDHNNFTKEDFVYKIKRILSDPRFKKNAVDLSHRFHDRPLNPKDTVAYWIEYVLRHNGAHHLKSQAVDTKWYQYFSLDLLVIAFVIITSLLYFFYNVISIILK |
| AgUGT343A4 | MFSPKMRHSSFFCSVLCIAACTIVTRAANILVFMPLPLKSHFRGFQPLFEELSHRGHNVTVVSSFPLDRPINNYTDIGPFINKERVRNVMELVNMNFITSAQLKWKLGIQLSETVMSHENMKKFLQSSSNSFDLVMIETFCQEYTVAMGHKFNAPVINLAPAMLWVSVSKWLHVPSTFSYIPDVCLQTAGDMDFVDRLKNTITGLIQSYVENYIYLPKMKEVMNKYITYEGWESRPPLERMLNNVSLTLVNSNYAIGVARPYLQGVVEVGGMHLKTPKSLPENLQTILDAADEGVIFFSFGTVVNLNDLPKEKLKIFLNVVQKLKQKVILKWVPKDNVKLSKNIMTGSWFPQNDILAHPNVRLFITHGGLHSIEETVNNAIPIVGVPFFADQYLNIKIVEQQGYGKLVNFFEMTEESFENAVKEVLSNARFKEMAMVQSQVFKDQPMKPLDRAVYWVEYVLRNGGAEHLKSDSLELNDLQYFLLDVSVIILVLTGLIIWSCYLIVAKCISKKLNTA |
| AgUGT343B2 | MKTYFVVLASASRLLLSLSLISTVLSPPTTLAANILAFMPMPLKSHFSGFQPMFEELARRGHNVTVVSAFPLKDRRVPNYTDVDVMPSRGVPDFDVMHLINSNFMISVTNRWFFANLLSNQLEQPNLKDFVRSDDNSFDLVLIESFLQEYTVALGHKFSAPVVNLSPSMVWVSASKWLHLPAIFSYVPDCCIGITDDMSFVDRLKNTIVGLMEMVVEDYLYIPMMKTKMSKHFAYTGWQSRPTLEQMLNNVSLTLMNAHHAVGVCRPYLPGVIEVGGMHIKEPKPLPKDLQDYIDSASHGVIFFSFGSIINLSNLPKEKLNSFLNVISRLKQKVIMKWVPDKSIKLPHNVKVGSWLPQNDILAHSNVKLFITHGGLHSIEEAVYYGKPVIGIPFFADQRSNMKVVEKNGYGKLITYNELTEESFGNAVEEVITNPTFKDKSMIQSQVYRDQPMKPLDRAVYWIEYVIRNDGAKYLKSDSIGLNTAQYFLFDITLFLFLLTVIIAWLVYCGTVKISSKCITN |
| AgUGT344A14 | MTSHYGVLMIAFGICLGICQVIVLPPVGAANILAVQTLAAKSHWNVMRAMLRALTDRGHTVTVFTPFLDGNRDGYTEVDVSGDLKVRVGLNVSRYLGMQTVPMFVAYMVNSTRTNCGSIFKDRRMREIFDSKSRIFDVVVAEALWLDCVSYAANVLQIPIIYVIPSPIVTHWERSYFGHFPNPAAVSNVMFWRSVPKTFADRLANTLHTVYGSWWLWREGRRHRQNKPLPSDAVDLVKPSLTFFNTHFITEPPRPLSPDTVQIGGIHLAPPEPIPRDILEFIDDAPHGVIYFTLGSVVLMSSLPENVVSVFRECFSQIPQKVLWKYEGDMKDKPKNVMTRKWFPQRDILCMFRNI |
| AgUGT344B4 | MYLPLLLILTFTFTGSWVTTPAKGARILAVETIGGKSHWNFMSAILRALVDNGHNVTVFTPFLDGNRENYTEFDTSLGSEKILDGQIEELMNKFGDPIKIIGQMSTMSRMLCNVVYENSKMKEILANPRSDDFDIVIIEPILSDCVSYLGAKLNLPLIYVMPIPTMGIMERYFTGDMSNPAVVSFNLAHFGIPKTFVQRAKNCAYLVYCTAILKFDEFRNRLTEPQEYDLYAPIPPSLIFVNRHFTIEPASPIQSNVVEIGGIHLKAPRKLPKDILEFIEQSPNGVVYFTFGSTVKMASLPEHIKKAFIESLAQIPHRVLIKYEDELEPMPRNVMTKKWLPQRDILVHPKVKLFISHGGISGLYEAIDGGVPVLGFGLFGDQPKNIDNLVNAGMAISMDIWSVTKENFLKNVLELLNNKKYTENAKTASRIFKDRSILPTNSVVYWTEYVLRHKGAPHLKSYALNLSWYQYYLLDLITLILSFIIVVFFVTYKMFKSISTYFSNYSRNNKSKSE |
| AgUGT345A2 | MRSGAIRSVVAVGSLLWSIGCLSTIAGHRILACEPSPGYSHWTVMSAVLESLLAAGHEVVCTAVHPATDRLAAHPNYTHVDMSSSLAGGLLQTARDVKYAHVMRIFRSNAFMVGQATARAQRVCEYISDMPEMQGILNGRGGEREFDAVVMESLHSECMSALPDRLGLPAVYVVPSPTVNWMPVATGAPDHPSYLGALLADRPTPATFGHRLANALVYAHTALVRWYNDGGRDRRWPEHRHTMLFVNTHHSIEPARPVGPNVLEIGGIHLNRPLEPLPGDLSDVLDKSNEFGVIVFTFGSLVAMDTLPANVLKAFKIVFSQLPQTIIWKYENDHMPDKPENVMLYKWLPQRAILQHPNVKLFISHGGMSGIYETVSAGVPVLGMPLFYDQPRNIQNLVDLGMALSMEINNLTRTTLYEAINRLIKDQSFSENAKRVSSLFRDRPMTPSESVVYWVEYLIRHGTEANIRPLSADSSWTSHFMLDICVALATALLILWFLKRAIAHAVFNKSIPSAVK |
| AgUGT348A2 | MKSVSFAAVVFCVLAQQIRSSEILVIFPTTAQSHYRVVRPLIHGLLDRGHRVLAITNFPDTAERANLSHIDISGLKPHSKFTTGRLGLAKMMSHITGNANAYATVLGHPPVVELLRSGRKFDLVIAEFFTSTPIFAPIAAVVDAPIVGFCPMIQFPWINDLMGMDTVASYMPTLISDSGDRMSFVQRVGNAVKSAIINVGFNRLNSRAIRDVVERHYDGLRTESAVEAMANLTMIMTNNYRSVFLPYPSLPGIVEVGGIHVVDEKPVSQDLNDFINDADHGVILFSLGSVVSESSMGTDKLYNILDAFSRLKQRVIMKFDDEKHKKRNTDECKSGQMVSTTRSSSASKGIIIYNTCWYYERNRDDTLRKTNSSHTDVWRSNCQYPFVGRKTSGCHYKIRTFEE |
| AgUGT349A2 | MKCVGLLLVALVGALSVAEGANILGVFPINGRSHWVVYESVMKALAARGHNVTVITSFPQKTPVANYTDVDVSATFPPAMNTVGIDLVLKYLASVFANQWFIADHQMNICRKHQKLPQVQALLHSDIKFDAVFTEIFGADCDVGFAYHFKAPLLSIMSSSHLPWSYDRVGGPDNPSYIPTIVTRAAGKMNFKERMINTFYYIYFKLAWKYHSEWPANELLKENFGPDVPHINEIIYNTSMVFVNGHFSLDGPRPLVPNMVEIGGIHVKPPRPLPKDILKFIDDSPNGVMFFTFGSLIRISTLPPSVLQMFKEVFAKLPIRVLWKYEAEMKDKPDNVYISNWMPQRDILSHPKVRMFMTHGGLLGVLEAVHSSVPIIGIPFFFDQPRNILKLVEQGSGIILDYETLTKDVLYDAIMKIINNDSYAINAKKLSKRFKDRPLNATETAVYWTEYVIRHKGAKHLRTAAVGMPWWKYYLVDVIGFIALIIFSVLYLIYFVLKTIYKKLFKKTEPKKKEKKN |
| AgUGT350A2 | MKFLFFTVLLLLYVGHCMSANILAFLPTFARSHYGGFQPLLKELAVRGHNVTVLSHFPLKNPPPNYHHIDVSIKERQDNNFSMLSIAPYLKPLIIPIGTLFFASQFTLETLNNIKVKEFIHSDGYQFDVVIFENFQHECFVSMSHKFGAHAIQLFPATPTAFPSQWYSQPFNPSYIPDPNSGYKDHMTLFERTINFLVTCLQFFLFPIFYMPKQNEIMLKHFNYTGSESRPSLEEMMKNISLTLINTHFTLGTPRPLVPSFIEVAGMHLKPSSKLPKDLQELMDNSPDGVVYFSFGSVVKGSHLPMYQVEMFLRQLSQIKQKVLWKWESDNLPELPPNVVVRKWFPQVDILGHPNCVLFITHGGIHSVEEAVYYGVPMLAISIFGDQLYNSIMMESRGAAIRLKYTELTEKRFGYSLQEILSNTSYKENAIKLCKIFHDQPIKPLDKAVYWIEYVIRHNGAHHLKTAGNKLNWFQFLSIDVMFVLIITIFFFFLFSFYAIKFIYKFVTEFRKYNIETDDDKKDN |
| AgUGT350B2 | MNSLVLIIVILITVNNGLTSNILAFLPSKARSHYGAFEPLFKELAEKGHNMTVLSPFPMKNPPPSYHHIQVEDEDLVMGFNPFDVARHFKPILVPFRSWFIWPKFTETILNKPSIQKLIHSEGLHFDLVLFENFYHECFVTLGHKFNAPVVQLFPSIPNAGVAQWHHNPYIGSYIPDINTGFCDNMSFIERLTNTVLSFIHTALGSFYYLPKQRDLMNKYFNYTGWETRPSIENMLNNISLTLINTHFSVGTPRPLVPSYVDVAGMHLKPASSLPEDLQDIMNNASDGVVYFSFGSVLKLTQLPKNEFEIFIRQLGKIKQKVLFKWESDNKIDFPPNIIARKWFPQVDILGHPNCILFITHGGIHSTEEAIYFGVPMLAISVFGDQLHNSLVMQNRGAAIQIKYSEFTEDVLEIALNKILNEKSFKQKATELSQIFHDQPLNSLNKAIYWIEYVIRYNGAHHLKTAAGQLTCFTDYPRNSLIHG |
| AgUGT351A3 | MKYITILVILLNFYVHAIKSSNILVFVPSPWKSHIIAFQPLFLELANRGHNVTVISKFTVKDPPPTYTQLVPSYNFDIDGRSDYLFRERSMYYSFIEDPMTRSTILVDTTLMFMSDPVIQKFIKYDQSSFDLVMIESFFQECAVALGHKYGAPVISIVPVTPWISVSRWANNPADLSYIKDFMLDGGKSLTFWERLANSFIGFYSLFVEPITYLPKLENMMNTYFQYPGYENRPTMAEMLKNISLSLIDSDITIFSSRPYVPSFIEVPGIHFRPKKKMDKFYTTCGILRI |
| AgUGT351A4 | MKFISILVFVAIHCALEIKSSNILVFVPSPWKSHVVSFEPLFLELAHRGHNVTVVSSFPMKNPPSNYTQIVPKYELDLRPITDSIMQNRNKYLFFLEEIYIRNIFGIYITKTYVSAPEIQKFIKEDQTKFDLVIIESFFQDCTVAMGHKYGAPVISIIPVAPYVSISRHAANPSDFSYIKDFKLNAGKSIDFQYRLLNTLFGLYSLFIEPITYIPLLEEIMDNHFQYPGYENRPKMTEMLENISLSFIDSDVSILSPRPYVPNFIEIPGIHIQPTKEMKKTLQNFMDTAQAGVVYFNFGTLLDTSRLPKPTLEVFVNVLGRLEQKVLFKWTNNDTQGFPDNFYVDSWFPQLNILRHPNCKLFITHGGIHGIMETIDAGVPFIGFPFFGDQFQNLKISQENGFGLINDIHTLNEDSFERDVKLILTDMRFTENAKKMSTIFHDRPMSALDTAVYWVEYVIRHKGAHHLRSTAVKLTWYQYLLLDVILFFIVISLLLIYIFYFITKCIIRSILKLFIKQKTD |
| SlUGT40R3 | MALAICLFFLLLSSSCEAYKALVVFGMPATSHSNLGRGVVRNLLKDGHEVTFITPIPIKDPPPNLHQIDVSSNFELLPLDLMKIERFLGPNSMPALPRFFVKMMMMNLVSKTMEHENVQKLLNDTIAHFDVVIVEWMFTSLSAGYATIFDCPLIWLIPVEVNSMTIGLVDAVPHPAYSTDPLSSYLPPFSFLERATEIWTRLQESVLGFLYYESKDAANYERIVVPQVQKRGRQAPPLSEVQYNASLVLGNSHVSMGLPLSLPQNYKPVGGYHIEEEVKPLPEDLEKIMMNSKNGVIYFSMGSNLKSKDWPEEIKRDLLKLFGELKQTVLWKFEEELPNVPKNVHILKWAPQPSILAHPKCVLFITHGGLLSTTETIHFGVPTIAIPVFGDQFINVKKSVARGFTLQVDLSYKLAADLKVAIEEMLSNPKYRQRVKELSYIYHDRPVKPGAELRHWVQHVVNTRGASHLRSPALQVPLYQRLYLDLVAFLSVAFIVLYMLIKKLYSRVRSKKIVNNKKRN |
| SlUGT46A6 | MRTLAILLVAIFAVNVQSARILGLFPHTGKSHQMVFDPLLRTLAERGHDVTVVSFFPIKNPPANYTIVSLEGLAAQGVETIDLSYFDSQNKLLNTLGIEKVIKQILDFQPLADMATGICSNIVDFVPLSNAMKKSYDVILVENFNSDCMLGLMHVHGLKAPYISLSSSAMMQWSADRIGVNDNPSYVPLVSSEFTSQMTFLQRLENTILNVYYKTWFRYAIQMKEKAIIEKRFGRRIPDLQEIAKNVSMMLVNTFHSLNGVRPLLPGVVEVGGMHLDHSRKPIPHYIERFLNESEHGVVLFSWGSLIKTASIPKYKEEIIVNALSKLKQRVIWKYENSNEEGTLTGNILKVKWIPQYELLQHEKVIAFIAHGGLLGMTEAISAGKPMLIVPFYGDQMVNGAAATTIGLGKAISYADMSEKSLLEGLQSVLSPEMRMSARRASKIWQDRIADPLDTAVYWVERVIRWGHQDPLHSTSKDMGFIEYNLLDVAAVILLSFVFLILVLRIVLNQILRLFGAGTSKKEKLH |
| DmeUgt35b | MLALRIGFLLLTLPASMQAARILAIFPFPGPSQYINVVPYLKELANRGHQVTSVNAFPQKKPVVNFRDVFIPDVFNNYKELINELSGPMNLWQENNFINKFFVSVTRCVLTNKEVTETLLAPGKDHFDLIIVEALRSDAYYGFAAHFNAPIIGISTFGTDWNIDALVGNESPLSYTPLATGGLTDRMTFLERLSNFVDTTVAWLNYRFVHMSEQEKMYAKYFPEASKRVQLTDLNRNFSLVLLNQHFSLSFPRPYVPNMIEVGGLHISHKPAPLPKDLEEFIQGSGEHGVIYFSLGSNVLSKDLPADRKELILKTFGSLPQRVLWKFEDDKLPGKPSNVFISKWFPQPDILAHPKVKLFITHGGLLSTIESIHHGKPVLGLPFFYDQFLNVRRPTQAGFGLGLDHTTMTQQEFKETIEILLKEPRFAQIARQMSERYRDQPMSPLDTAIWWTEYVLRHKGAYHMRVAGQDLGFFAYHSLDVIGVLLGGALLLVAIIVGVLGKLTDFGNAKKKLKSK |
